# Diclofenac Disrupts the Circadian Clock and through Complex Cross-Talks Aggravates Immune-Mediated Liver Injury—A Repeated Dose Study in Minipigs for 28 Days

**DOI:** 10.3390/ijms24021445

**Published:** 2023-01-11

**Authors:** Saravanakumar Selvaraj, Jung-Hwa Oh, Seokjoo Yoon, Jürgen Borlak

**Affiliations:** 1 Centre for Pharmacology and Toxicology, Hannover Medical School, Carl-Neuberg-Str. 1, 30625 Hannover, Germany; 2 Department of Predictive Toxicology, Korea Institute of Toxicology, Daejeon 34114, Republic of Korea

**Keywords:** diclofenac, drug-induced liver injury, hepatitis, liver clock, liver pathology, genomics, histopathology, immunohistochemistry, transcriptional networks, cellular metabolism

## Abstract

Diclofenac effectively reduces pain and inflammation; however, its use is associated with hepato- and nephrotoxicity. To delineate mechanisms of injury, we investigated a clinically relevant (3 mg/kg) and high-dose (15 mg/kg) in minipigs for 4 weeks. Initially, serum biochemistries and blood-smears indicated an inflammatory response but returned to normal after 4 weeks of treatment. Notwithstanding, histopathology revealed drug-induced hepatitis, marked glycogen depletion, necrosis and steatosis. Strikingly, the genomic study revealed diclofenac to desynchronize the liver clock with manifest inductions of its components CLOCK, NPAS2 and BMAL1. The > 4-fold induced CRY1 expression underscored an activated core-loop, and the dose dependent > 60% reduction in PER2mRNA repressed the negative feedback loop; however, it exacerbated hepatotoxicity. Bioinformatics enabled the construction of gene-regulatory networks, and we linked the disruption of the liver-clock to impaired glycogenesis, lipid metabolism and the control of immune responses, as shown by the 3-, 6- and 8-fold induced expression of pro-inflammatory CXCL2, lysozyme and ß-defensin. Additionally, diclofenac treatment caused adrenocortical hypertrophy and thymic atrophy, and we evidenced induced glucocorticoid receptor (GR) activity by immunohistochemistry. Given that REV-ERB connects the circadian clock with hepatic GR, its > 80% repression alleviated immune responses as manifested by repressed expressions of CXCL9(90%), CCL8(60%) and RSAD2(70%). Together, we propose a circuitry, whereby diclofenac desynchronizes the liver clock in the control of the hepatic metabolism and immune response.

## 1. Introduction

Diclofenac is a nonsteroidal anti-inflammatory drug (NSAID) and commonly used to treat pain and inflammation in conditions of osteoarthritis and musculoskeletal disorders [1,2]. Its anti-inflammatory, analgesic and anti-pyretic effects are caused by inhibition of cyclooxygenase 1 and 2. This impairs the arachidonic acid metabolism and the production of prostaglandins. Diclofenac inhibits the production of leukotrienes and suppresses thromboxane-prostanoid receptor signaling. Additionally, its analgesic activities are attributed to modulation of the nitric oxide–cGMP nociceptive pathway, inhibition of NMDA-receptor-mediated hyperalgesia and the blocking of substance P [3,4,5].

Despite its proven benefits, diclofenac medication is associated with adverse drug reactions (ADRs), especially cardiovascular, gastrointestinal, hepatic and in the kidneys [5,6,7,8]. Commonly reported ADRs are serum aminotransferase elevations among arthritis patients [9,10,11], and a population-based study from Iceland defined diclofenac as the second most common drug-induced liver injury (DILI) causing agent among outdoor patients [12]. Consequently, the European Medical Agency restricted the use of diclofenac, particularly in patients with heart or circulatory disorders.

The mechanism by which diclofenac induces liver injury is only partly understood. Mitochondrial toxicity and the production of reactive metabolites, e.g., benzoquinone-imine intermediates, are thought to cause hepatotoxicity while diclofenac acyl glucuronides damage bile duct epithelium. In fact, diclofenac’s metabolism is complex; it involves hydroxylated and conjugated metabolites [13]. Given its extensive metabolism, diclofenac causes oxidative stress that may result in critically depleted intracellular glutathione stores [14,15] to induce cytotoxicity and mitochondrial permeability transitions [3,6,16]. In addition to direct effects, reactive metabolites produce protein adducts which function as neo-antigens and are sensed by antigen-presenting cells to trigger B and T cell responses [17]. Moreover, allelic variants of the genes coding for CYP2C8, UGT2B7 and ABCC2 sensitize individuals to diclofenac hepatotoxicity [18].

Recently, we reported diclofenac’s potential to cause immune-mediated and allergic hepatitis in mice [19] and dogs [20]. We observed pro-inflammatory cytokine and chemokine release by injured cells and the migration and infiltration of immune cells to sites of injury. Specifically, increased expression of tumor necrosis factor (TNF)-α, interferon (IFN)γ, interleukins (IL-1, IL-6, IL-7, IL-17, IL-18) and chemokine ligands (CXCL1, CXCL2, CXCL8, CXCL12, CXCL13, CXCR4, CXCR7 and CCL2) aggravated liver toxicity [21]. Additionally, liver hypersensitivity reactions involved oxidative stress, macrophage polarization, mastocytosis and complement-activation-related pseudoallergy (CARPA), as well as an erroneous programming of the innate and adaptive immune system [20]. Additionally, NSAIDs have the potential to cause drug-induced steatosis to result in the altered expression of genes coding for lipogenesis, lipid transport, lipid droplet growth, ER stress and fatty acid oxidation [20].

Our immunogenomic and molecular pathology studies in mice and dogs informed on important species differences in immune-mediated hepatitis. Given the growing role of minipigs in translational immune safety studies [22,23,24], we investigated diclofenac’s potential to cause organ toxicity at clinically relevant and above doses after repeated daily dosing for 28 days. We performed whole genome scans of minipig liver and kidney to delineate reasons for organ pathologies, and gene expression profiling studies alerted us to immune, stress and inflammatory responses, while serum biochemistries and molecular pathology studies pointed to a novel mechanism whereby diclofenac modulates the circadian rhythm and immunity of the hepatic and neuroendocrine system. Indeed, in a recent review, Mukherji et al. highlighted the importance of the circadian clock in liver function and fatty liver disease [25], and our findings imply a complex interplay between diclofenac, the liver clock and the hypothalamic–pituitary–adrenal axis with stress-induced adrenocorticopic hormone (ACTH) release augmenting adrenal glucocorticoid synthesis to modulate the immune response.

## 2. Results

### 2.1. Treatment Related Clinical Signs

Depicted in supplementary Figure S1 are body weight (panel A) and food consumption (panel B) over 28 days, and we recorded individual organ weights at the end of the study (panel C and D). We observed significant reductions in body weight gain for one animal for the low- and high-dose treatments, and these animals showed decreased food consumption (supplementary Figure S1, panel B). Based on adjusted organ-to-body weight ratios (panels D2 and E2), we observed statistically significant increases in adrenal and prostate weights at the high-dose regimen. Conversely, the low-dose treatment caused a reduction in thymus, but a significant increase in kidney weight. Notably, the increase in adrenal and prostate organ weights was clearly dose related, and reached statistical significance at the high-dose regimen. However, for thymus and kidney, a clear dose-related change was not observed, and, because of significant inter-individual variabilities, the organ weight changes became significant only at the lower dose.

### 2.2. Serum Biochemistry

Supplementary Figure S2 summarizes serum biochemistry findings. Diclofenac-related abnormalities included a significant but mild increase in AST activities at day 6 of treatment and reductions in albumin (hypoalbuminemia), total protein (hypoproteinemia), BUN (day 6 of treatment; strict dose dependent), ALT, bilirubin, glucose and serum creatinine at day 14 of treatment. Experimental and clinical evidence was suggestive of diclofenac to increase insulin release from beta cells by inhibiting ATP-sensitive K-channels, and this provides a rationale for the observed hypoglycemia [26,27]. 

### 2.3. Hematology

We performed blood smears on days 6, 8, 14 and 28 of diclofenac treatment (supplementary Figure S3) and observed a dose- and time-dependent increase in WBC and neutrophil count from day 8 onwards. Similarly, diclofenac treatment caused a dose-dependent increase in monocyte and lymphocyte counts on days 8 and 14, respectively.

### 2.4. Serum Electrolyte Analysis

Supplementary Figure S4 depicts significant reductions in serum Ca^2+^, Cl, Na and potassium after low-dose diclofenac treatment for 14 and 28 days. The electrolyte imbalances signify kidney injury following drug treatment, whereas the observed hypokalemia likely stems from an adrenal gland disorder, as detailed below. 

### 2.5. Histopathology

We performed a range of histological stains to examine liver morphology in response to diclofenac treatment. In addition, we investigated the regulation of components of the liver clock and markers of inflammation by immunohistochemistry. 

**Figure 1:**
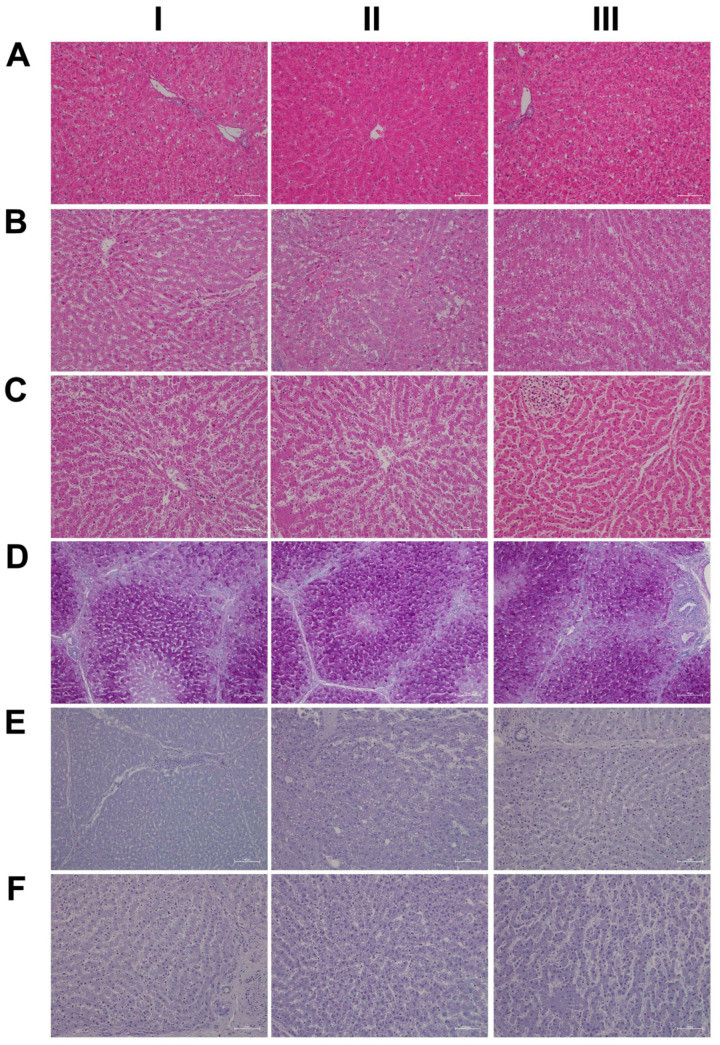
Liver HE and PAS staining of control and diclofenac-treated animals after daily dosing for 28 days. **Panel AI–III:** HE staining of three individual control animals with normal morphology of the liver. **Panel BI–III:** HE staining of three low-dose-treated animals highlighting degenerative cell changes and vacuolation of hepatocytes due to drug-induced steatosis. Note the eosinophilic hepatocytes and the sinusoidal dilatation (BI), the hydropic swelling of hepatocytes within an inflamed liver lobule (BII–III) and the hepatocyte regenerative activity (BI–III). **Panel CI–III:** HE staining of three high-dose-treated animals. The spectrum of morphological changes included a range of degenerative cell changes including shrunken hepatocytes, vacuolation (CI–II) and inflammatory cell infiltrates (CI). Depicted in the upper left quadrant of panel CIII is a fresh granuloma. A full description of the morphological changes is given in the main text. **Panel DI–III:** PAS staining of three individual control animals with abundant hepatic glycogen expression. Perivenous hepatocytes store less glycogen, which implies zonation of glycogen storage within the liver lobule. **Panel EI–III:** PAS staining of three low-dose-treated animals. Diclofenac treatment caused almost complete glycogen depletion. Additional findings included portal inflammation (EI), hepatic vacuolation (EII) and marked Kupffer cell infiltration (EIII). **Panel FI–III:** PAS staining of three high-dose-treated animals. Diclofenac treatment caused almost complete glycogen depletion. Other findings include portal inflammation with a partially destructed portal field (FI, lower right quadrant) and sinusoidal dilatation (FII–III).

Shown in Figure 1 panels A–C are hematoxylin and eosin-stained liver sections of the control, low- and high-dose treated animals, and panels AI–III are liver sections of three individual control animals. The hepatocytes are normal-sized and arranged in a trabecular-like pattern, and the nuclei are regular with small nucleoli. Intermingled are a few binucleated hepatocytes (AIII), and the sinusoids are narrow. The hepatocytes are rich in glycogen, as denoted by the softly granular appearance of the cytoplasm (see also below, PAS stain), and resident macrophages are in a quiescent state. The biliary epithelium is intact with no sign of morphological abnormalities. Panel B depicts liver sections of three low-dose treated animals. The hepatocytes are shrunken, highly vacuolated (BI–III), irregularly shaped, and the cytoplasm of hepatocytes is slightly basophilic; notwithstanding, there are patches of smaller-sized eosinophilic finely granulated hepatocytes with still dense, often doubled nucleated hepatocytes, which indicates fresh regenerates. The basophilic hepatocytes show the stigmas of recurrent regeneration due to an obviously high cellular turnover. We observed disseminated apoptotic cells with remnants of pyknotic nuclei. Notwithstanding, hydropic and lytic cell changes dominate, especially in zones of injury with increased numbers of vacuolated cells. Additional morphological changes include dilated sinusoids; its mottled appearance signifies differences between hepatic lobules in the inflammatory response to diclofenac treatment. The parenchymal injuries occupy zones 2 and 3 (BI–III); however, zone 1 is characterized by hypertrophy of the trabeculae. Shown in panel CI–III are liver sections of three high-dose treated animals. Note the dose-related vacuolar degeneration of the shrunken hepatocytes. Their shrinkage indicates a marked preexisting hydropic swelling associated with lytic cell changes and dispersed cell detritus in the widened sinusoids. The resident macrophages are activated, and leucocytes are marginating in the sinus (CII). Focal inflammatory infiltrates are present, either florid around a central vein (CI) or declining with granulomatous reaction (CIII). At high-dose, diclofenac treatment caused portal inflammation. Shown in panel CII is a partially destructed portal field with inflammatory infiltrates that extend to the rim of the portal field and the liver parenchyma (interface). The morphological appearance mimics features of an autoimmune-like hepatitis and, overall, we observe a dose-related increase in drug-induced inflammation and liver cell degeneration. Figure 1D–F depicts the PAS stain. The liver sections of control animals are rich in glycogen, as evidenced by the dark magenta staining of hepatocytes (panel D). Clearly visible is the demarcation of liver lobules and the pronounced periportal zonation of glycogen storage. Conversely, there is less glycogen storage in zone 3 hepatocytes and around the central vein (DI–III). Diclofenac treatment caused severe glycogen depletion (panels E and F) at low- and high-doses, and is therefore dose independent. This hallmarks a metabolic disorder and may be linked to increased insulin release induced by diclofenac treatment. In fact, NSAIDs have been reported to increase insulin release from ß-cells through the modulation of K-channels [26,27]. Shown in panel E1 are portal field inflammatory infiltrates arodizing the limiting plate (combined with some brown pigment). Once again, hepatocytes are shrunken, vacuolated and the sinusoids are dilated.

**Figure 2:**
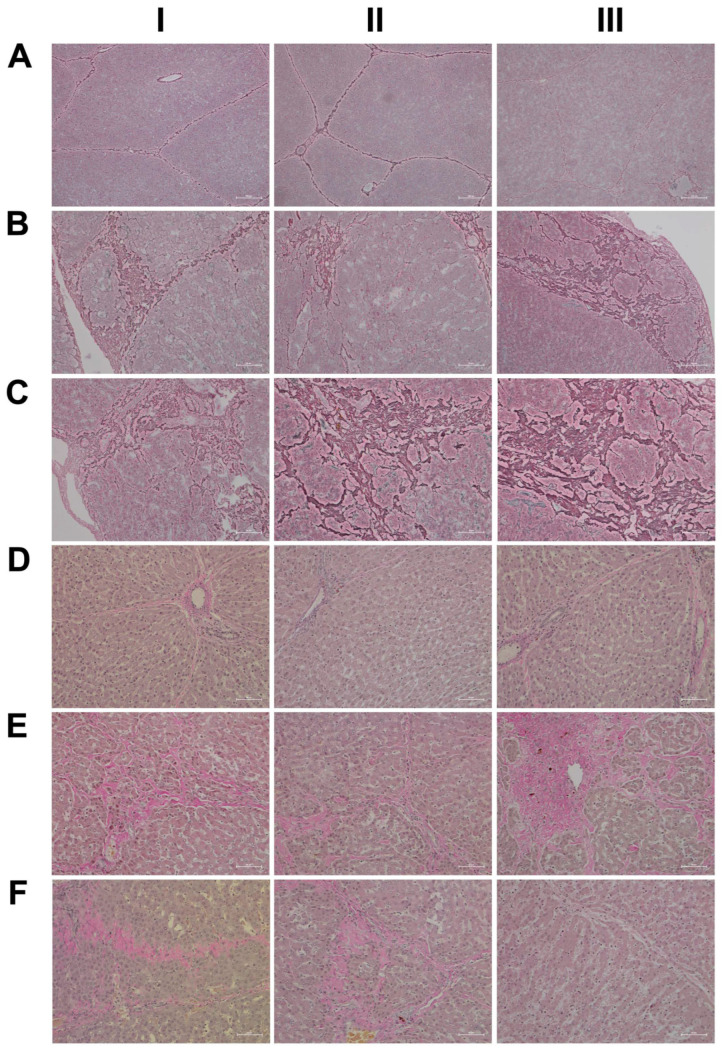
Silver and Elastica van Giesen stain of control and diclofenac-treated animals after daily dosing for 28 days. **Panel AI–III:** Silver staining of three individual control animals with normal morphology of the liver and no sign of enhanced extracellular matrix deposition. **Panel BI–III:** Silver staining of three low-dose-treated animals. Diclofenac treatment caused enhanced deposition of collagen and reticulin fibers around damaged liver lobules. **Panel CI–III:** Silver staining of three high-dose-treated animals. Diclofenac treatment caused defective wound repair with enhanced deposition of collagen and reticulin fibers around damaged liver lobules. **Panel DI–III:** EvG staining of three individual control animals with normal morphology of the liver and no sign of enhanced elastic and collagen fiber formation. Note the red staining of the collagen fibers of connective tissue around the blood vessel in a portal triad is normal (DI, III). **Panel EI–III:** EvG staining of three low-dose-treated animals. Diclofenac treatment caused enhanced collagen fiber deposition within harmed hepatocytes (EI) and at the rim of a liver lobule (EII). Once again, evidence for defective wound repair has been obtained, and the enhanced deposition of collagen fibers replaces damaged zone 3 hepatocytes around a central vein (EIII). **Panel FI–III:** EvG staining of three high-dose-treated animals. Diclofenac treatment caused enhanced collagen fiber deposition.

Given the importance of the reticular fibers in wound healing, we assessed the collagen fibers in liver sections of the control and diclofenac-treated animals (Figure 2A–C); shown in panel AI–III are three individual control animals. The silver stain highlights the reticular fibers of the interlobular connective tissue and the fibers around blood vessels. Following low-dose diclofenac treatment for 28 days, the fibers surrounding hepatocytes were stained more intensely (BI), and the reticulin stain hallmarks subacute hepatic necrosis with enhanced deposition of collagen fibers following cell death of hepatocytes (BI–III).

We regard the enhanced deposition of reticular fibers as defective wound healing, and, especially in areas of severe necrosis, the reticulin framework appeared bulky and convoluted (BIII). At high-doses, a similar picture emerges, and although a dose-related increase in the silver stain of collagen fibers is visible, the convolutes often have a preferred association with portal fields (BII upper right corner, CI–II). Moreover, we employed the EvG stain to assess connective tissue and its associated extracellular matrix (ECM) proteins (elastin, collagen, etc.) in response to diclofenac treatment (panel D–F). As shown for three individual control animals, elastin is a minor component of connective tissue in the liver (DI–III), and only fibers surrounding blood vessels and within portal triads stain-positive. Unlike the controls, diclofenac treatment at low- and high-doses caused an increased deposition of collagenous matrix (red fibers) in the liver parenchyma. Intermingled are micronodular hepatocellular regenerates (EI–II; FI–II), and this hallmarks a healing response to recurrent inflammatory infiltrations, mainly in the vicinity of portal fields, and are the second feature in addition to the zonal injuries of diclofenac-induced hepatitis. Focally, a broad fibrosis extends to the central vein and includes the initial deposition of elastin fibers (black fibers; EIII). Although collagen fibers can be degraded and the associated fibrotic scar might resolve over time, the combined irregular nodular regenerates indicate not only a preceding necrosis of liver cells, but also damage of the stromal framework. Therefore, a permanent disorder of the microcirculation could be a sequel. An addition of elastic fibers carries the risk of permanent scarring as elastin is highly resistant to digestion by proteases. The accumulation of elastic fibers in diclofenac-treated animals is not dose related. Together, we consider an enhanced ECM deposition as defective wound repair and early signs of fibrosis.

**Figure 3:**
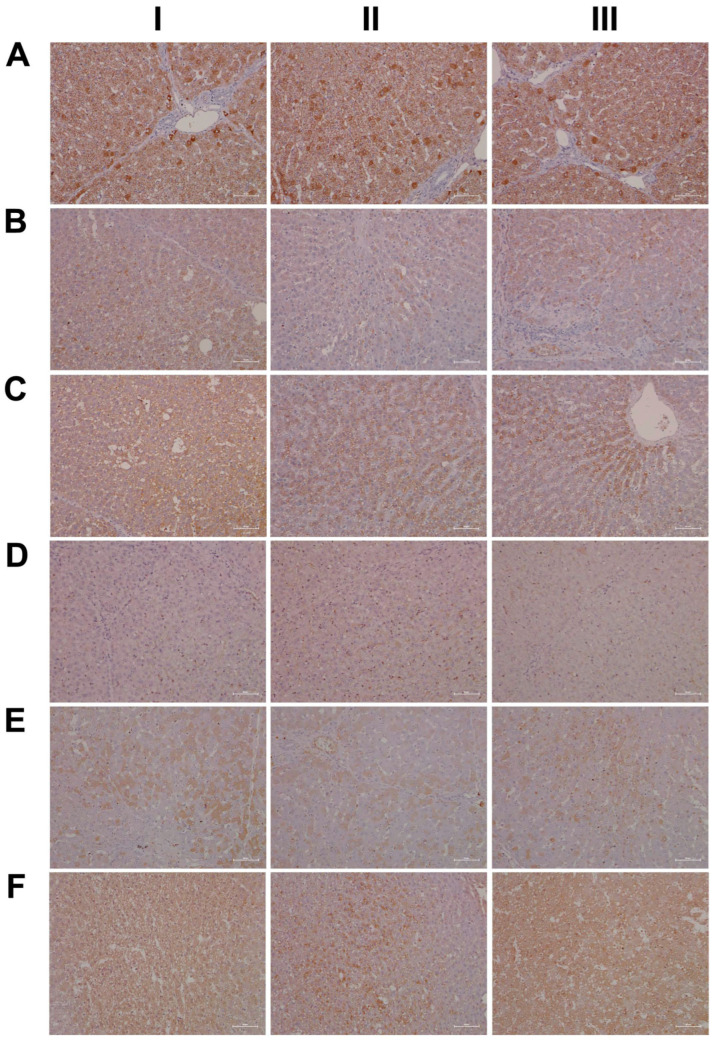
Immunohistochemistry of carbamoyl-phosphate synthetase 1 (CPS1) and ELOVL fatty acid elongase 2 (ELOVL2) in liver sections of control and diclofenac-treated animals after daily dosing for 28 days. CPS is a key enzyme in the urea cycle and catalyzes the rate-limiting step in the detoxification of ammonia and ELOVL2 functions in lipid metabolism and PUFA elongation, thereby reducing the production of inflammatory lipids. **Panel AI–III**: CPS1 staining of three individual control animals. All hepatocytes express CPS1; however, some express the protein more abundantly. **Panel BI–III**: CPS1 staining of three low-dose-treated animals. Diclofenac treatment caused marked reduction in CPS1 expression. **Panel CI–III**: CPS1 staining of three high-dose-treated animals. Diclofenac treatment caused marked reduction in CPS1 expression. **Panel DI–III**: Minimal ELOVL2 cytosolic staining of three individual control animals. **Panel EI–III:** ELOVL2 staining of three low-dose-treated animals. Diclofenac treatment caused induction of ELOVL2 with a mosaic-like expression pattern among regenerating hepatocytes. **Panel FI–III:** ELOVL2 staining of three high-dose-treated animals. Diclofenac treatment caused marked expression of the protein.

For its central role in nitrogen metabolism and ammonia detoxification, we assessed the expression of carbamoyl phosphate synthetase 1 (CPS1) by immunohistochemistry. Importantly, CPS1 ko mice die from hyperammonemia within 24 h after birth [28], and this enzyme catalyzes the conversion of ammonia to carbamoyl phosphate, i.e., the initial step in the urea cycle. Drug-induced urea cycle dysfunction leads to liver injury and mitochondrial toxicity, and the morphological changes in the liver of patients diagnosed with urea cycle disorders were recently summarized [29]. We employed the HepPar1 monoclonal antibody to assess the expression of CPS1 [30]. Shown in Figure 3 are liver sections of control and diclofenac-treated animals. Panel AI–III depicts three individual control animals, and we observed abundant staining of CPS1 with some hepatocytes displaying marked cytosolic expression of the enzyme. Conversely, diclofenac treatment at low- and high-doses significantly repressed CPS1, while inflamed hepatocytes (BII lower right quadrant) and severely injured hepatocytes (BIII, portal triad lower left quadrant) failed to express the protein. Similarly, the high-dose regimen (CI–III) markedly repressed CPS1 expression. 

Diclofenac treatment caused hepatic steatosis, and the link between aberrant lipid metabolism and hepatic inflammation has been the subject of several reviews and original works [31,32,33,34]. Indeed, a recent study highlighted the role of the macrophage-derived endogenous lipid mediator PCTR1 (protectin conjugates in tissue regeneration 1) in alleviating hepatic inflammation by augmenting fatty acid desaturation (FADS1 and FADS2) and polyunsaturated fatty acid (PUFA) elongation via the enzyme very long-chain fatty acid elongase 2 (ELOVL2) [35]. We found ELOVL2 transcript expression 4-fold induced, and therefore, we investigated its protein expression in the control and diclofenac-treated animals. Depicted in Figure 3 panels DI–III are liver sections of three individual control animals, and most hepatocytes showed a faint cytosolic expression of the enzyme. However, the Kupffer cells abundantly expressed ELOVL2. Following diclofenac treatment, we observed a dose-related increase in ELOVL2 expression. At the lower dose (EI–III), animals presented a mosaic pattern of ELOVL2 expression among regenerating hepatocytes, while at the higher dose (FI–III), the expression of the enzyme was markedly increased. Additionally, shown in panel EI are ELOVL2 positive round cell infiltrates and activated macrophages.

**Figure 4:**
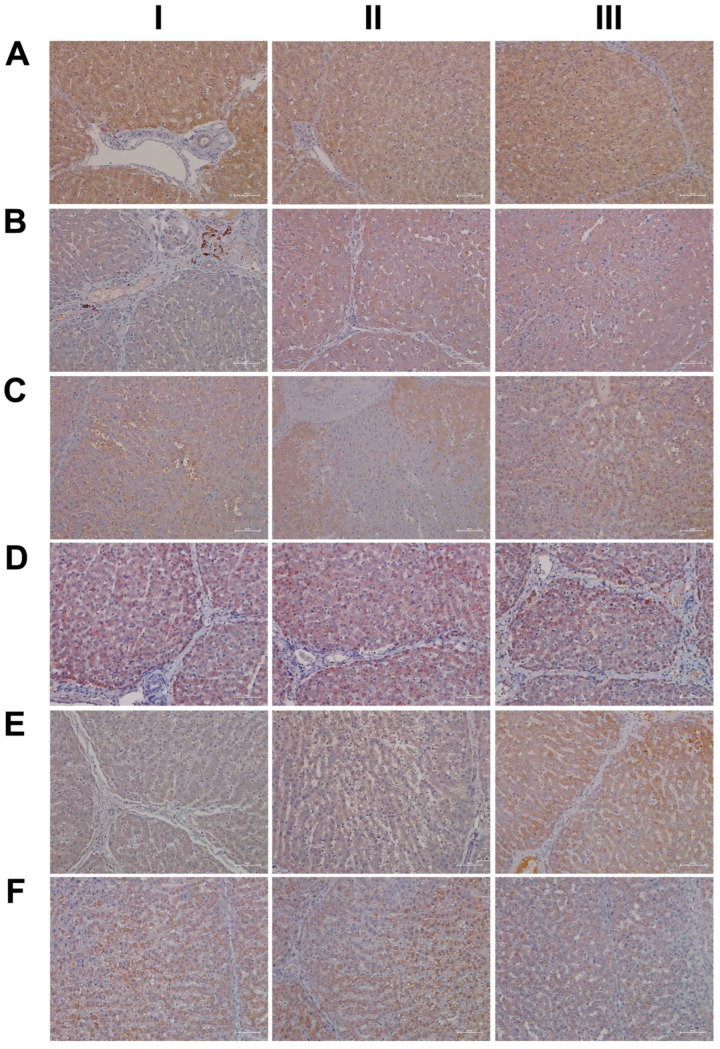
Immunohistochemistry of superoxide dismutase SOD1 and SOD2 in liver sections of control and diclofenac-treated animals after daily dosing for 28 days. Superoxide dismutase 1 and 2 are of critical importance in alleviating oxidative stress and reactive oxygen species. **Panel AI–III**: SOD1 staining of three individual control animals. Hepatocytes abundantly express this cytosolic localized enzyme. **Panel BI–III**: SOD1 staining of three low-dose-treated animals. Diclofenac treatment caused marked reduction in SOD1 expression. Shown in BI is a portal field with SOD1-positive Kupffer cell infiltrates. **Panel CI–III**: SOD1 staining of three high-dose-treated animals. Severely harmed hepatocytes fail to express SOD1. **Panel DI–III**: SOD2 staining of three individual control animals. Hepatocytes abundantly express this mitochondrial localized enzyme. **Panel EI–III**: SOD2 staining of three low-dose-treated animals. Diclofenac treatment caused marked reduction in SOD2 expression. **Panel FI–III**: SOD2 staining of three high-dose-treated animals. Diclofenac treatment caused marked reduction in SOD2 expression, and severely harmed hepatocytes do not express the protein.

A further point of considerable importance is diclofenac’s extensive metabolism in the liver and the production of reactive metabolites [36]. Superoxide dismutase plays a key role in the detoxification of reactive oxygen species by catalyzing the hydrogen peroxide reaction. We assessed the regulation of cytosolic SOD1 and the mitochondrial SOD2 in the control and diclofenac-treated animals (Figure 4A–F). Among controls, cytosolic SOD1 expression is marked in hepatocytes (panel AI–III). Shown in AI is a portal triad in which the biliary epithelium stained positive as well. Conversely, diclofenac low- and high-dose (B–C) treatments caused significant reductions in SOD1, with severely harmed hepatocytes failing to express the protein. For instance, BI depicts a high power field magnification of a portal triad with adjacent micronodular regenerates, indicating insufficient remodeling after focal destructive inflammatory infiltrations. Panel BII is a further example of a mosaic-like pattern of immature and mature cells of zone 1 hepatocytes, and high-dose animals present distorted trabeculae and variable cell size, thus reflecting recurrent regeneration throughout the entire liver lobule. Note the marked SOD1 expression in histiocytes/macrophages, but its minimal expression in the surrounding hepatocytes. At the higher dose and within the entire liver lobule, there are patches of hepatocytes failing to express the protein (CI–III). We also investigated the expression of mitochondrial SOD2; panels D–F are representative images of liver sections from control and diclofenac-treated animals (Figure 4). Unlike controls (DI–III) and as seen with low (EI–III) and high-dose (FI–III) treatments, injured and highly vacuolated hepatocytes express little to moderate SOD2 protein. Interestingly, SOD2 associates with the lipid droplet monolayer of steatotic hepatocytes (FII), and the antibody visualizes the fusion of smaller lipid droplets to larger ones (FII). There are some disseminated (FI, left side, FII, upper center) or cohesive immature regenerates (EIII, right side), which do not express the SOD2 protein.

**Figure 5:**
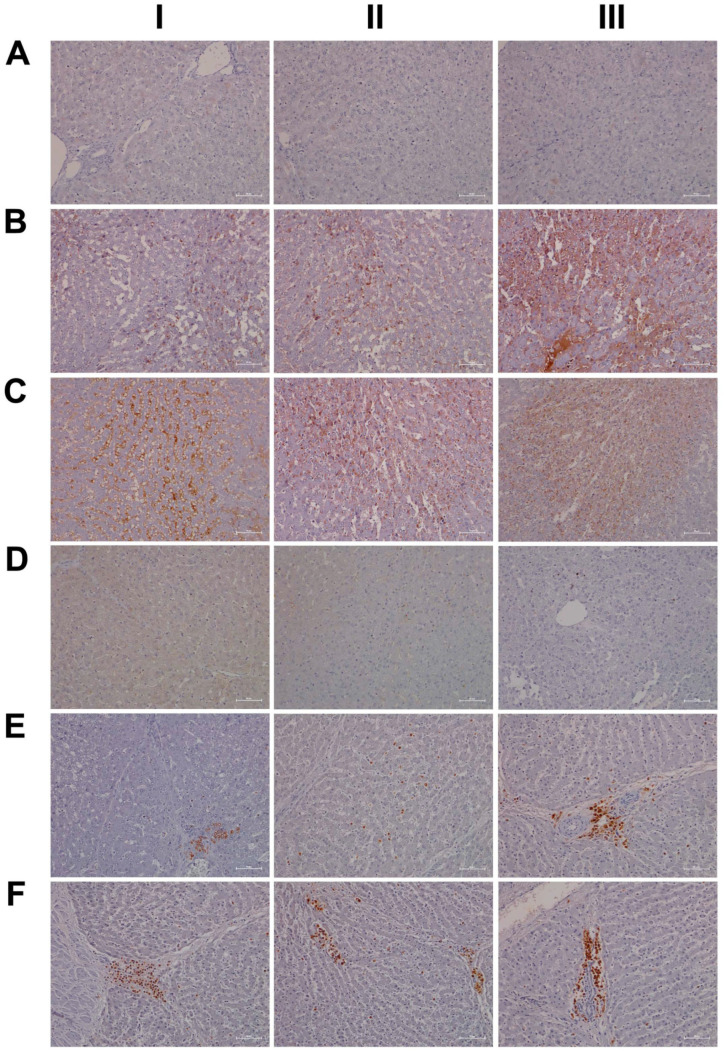
Immunohistochemistry of hypoxia inducible factor 1α (HIF1α) and myeloperoxidase (MPO) in liver sections of control and diclofenac-treated animals after daily dosing for 28 days. HIF1α is a transcription factor and of critical importance in regulating cell metabolism in conditions of hypoxia/ischemia, and MPO is an enzyme that catalyzes the production of diclofenac reactive metabolites which damage liver parenchyma. **Panel AI–III:** Liver sections of three individual control animals with no HIF1α expression. **Panel BI–III:** HIF1α staining of three low-dose-treated animals. Diclofenac treatment caused induced HIF1α expression, especially in activated/inflammatory macrophages (BI–II) and harmed hepatocytes (BIII). **Panel CI–III:** HIF1α staining of three high-dose-treated animals. Note the induced HIF1α expression in sinusoidal endothelium of an inflamed liver lobule (CI). The protein also “dresses” the monolayer of vacuoles/lipid droplets. HIF1α expression is variable with hypoxia damaged/pre-apoptotic hepatocytes expressing the protein abundantly (CII-CIII). **Panel DI–III**: None of the resident macrophages express MPO in the liver sections of three individual control animals. **Panel EI–III**: Depicted are the liver sections of three low-dose-treated animals. Panel EI shows a swarm of MPO-positive neutrophilic granulocytes; E2 exemplifies mixed inflammatory infiltrates of activated macrophages and neutrophils which abundantly express MPO, and EIII highlights infiltrating granulocytes and Kupffer cells in an inflamed portal field (EIII). **Panel FI–III:** Liver sections of three high-dose-treated animals. Depicted is the swarm-like migration of MPO-positive Kupffer cells at the rim of a necrosis (FI). Note the MPO-positive mixed intralobular (FII) and portal inflammatory infiltrates (FIII).

Furthermore, there is evidence for the HIF1α transcription factor to attenuate oxidative stress and its associated liver injury through the up-regulation of oxidative defense genes [37]. Shown in Figure 5 are liver sections of three individual control animals (AI–III) and the expression of HIF1α is minimal. In strong contrast, diclofenac treatment caused moderate to marked HIF1α expression at low (BI–III) and high-doses (CI–III), and HIF1α positive hepatocytes seem to regenerate and are less injured. There is also significant sinusoidal and endothelial staining of HIF1α and the protein “dresses” the lipid droplet monolayer (panel CI). In addition, there is mitochondrial HIF1α expression, as indicated by its seed-like appearance in the cytosol of hepatocytes (panels CII–III). 

In an earlier investigation, we highlighted the critical role of myeloperoxidase (MPO) in the metabolism of diclofenac to reactive metabolites, especially in the production of benzoquinone imine intermediates [20]. In fact, Kupffer cells and also neutrophils are rich in MPO, and this enzyme catalyzes the diclofenac-reactive metabolism to result in oxidative damage of the liver parenchyma. Shown in Figure 5 panels DI–III are liver sections of three individual control animals and most of the Kupffer cells are MPO negative. There are only two slightly MPO-positive macrophages in the upper half of panel DIII, and none of the controls contain neutrophilic infiltrates. In strong contrast, diclofenac treatment caused marked induction of MPO at the low- (EI–III) and high-dose (FI–III) regimens and a remarkable recruitment of numerous neutrophils and some monocytes to zones of injury. We noticed MPO-negative lymphocytes marginating in the dilated sinuses (EII). Therefore, diclofenac treatment elicits an inflammatory response within the lobules (FII) which is dominated by neutrophils, varying numbers of macrophages (EIII, FI–III) and MPO-negative lymphocytes (EIII, FIII) at the lobular periphery. 

Depicted in panel EI is an example of an early histiocytic granuloma at the parenchymal edge of a portal field, which we consider to be at the resorption stage of an otherwise florid foci. 

EIII exemplifies portal inflammation composed of a mixture of neutrophils and macrophages. Moreover, panel EII is suggestive of the transendothelial trafficking of neutrophils within dilated sinusoidal vasculature [38]. These findings imply diclofenac treatment to cause sterile inflammation with damaged hepatocytes sending alarm signals (DAMPs) to promote neutrophil recruitment and their activation. A similar picture emerges at the higher treatment dose and, once again, the results of three individual animals are given. Note the neutrophil swarm migrating into a necrotic zone at the edge of a focal necrosis (F1), as well as the portal inflammation depicted in FIII with intact bile ducts. Panel FII exemplifies lobular inflammation and sinusoidal trafficking of neutrophils.

**Figure 6:**
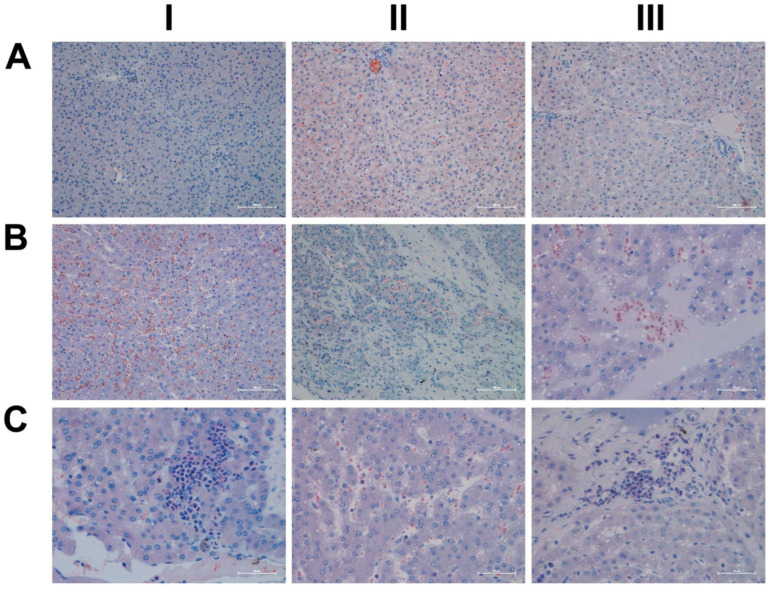
Chloroacetate esterase (CAE) staining for neutrophils in liver sections of control and diclofenac-treated animals after daily dosing for 28 days. **Panel AI–III:** Liver sections of three individual control animals. There are no observable neutrophilic infiltrates. However, diclofenac treatment at the low- and high-doses caused marked infiltration by neutrophils. **Panel BI–III**: Liver sections of three low-dose-treated animals with intense staining of monocytes/macrophages in BI. Note the extended necrosis in BII with its monocytic infiltrates, and the diffusely red stained macrophages in a necrotic lesion (BIII). **Panel CI–III**: Liver sections of three high-dose-treated animals. Shown in panel CI is a swarm of mainly banded neutrophils in an inflamed liver lobule and a mixture of segmented neutrophils and macrophages in the widened sinusoids (CII). Depicted in panel CIII are portal inflammatory infiltrates consisting of banded and segmented neutrophils and macrophages.

We also assessed neutrophils by the chloroacetate esterase stain (CAE, Figure 6); shown in panel AI–III are liver sections of three individual control animals. We did not observe neutrophilic infiltrates in the liver sections of control animals. However, diclofenac treatment at low- and high-doses caused marked infiltration by neutrophils. Depicted in panel BI–III are liver sections of three low-dose-treated animals. Note the intense staining of monocytes/macrophages in BI, the extended necrosis in BII with its monocytic infiltrates and the diffusely red stained macrophages in a necrotic lesion (BIII). Furthermore, panel CI exemplifies a swarm of mainly banded neutrophils in an inflamed liver lobule of a high-dose-treated animal (CI), the mixture of segmented neutrophils and macrophages in the widened sinusoids (CII) and the portal inflammatory infiltrates consisting of banded and segmented neutrophils and macrophages of a high-dose-treated animal (CIII). 

### 2.6. Genomic Responses in Liver and Kidney to Diclofenac Treatment

**Figure 7:**
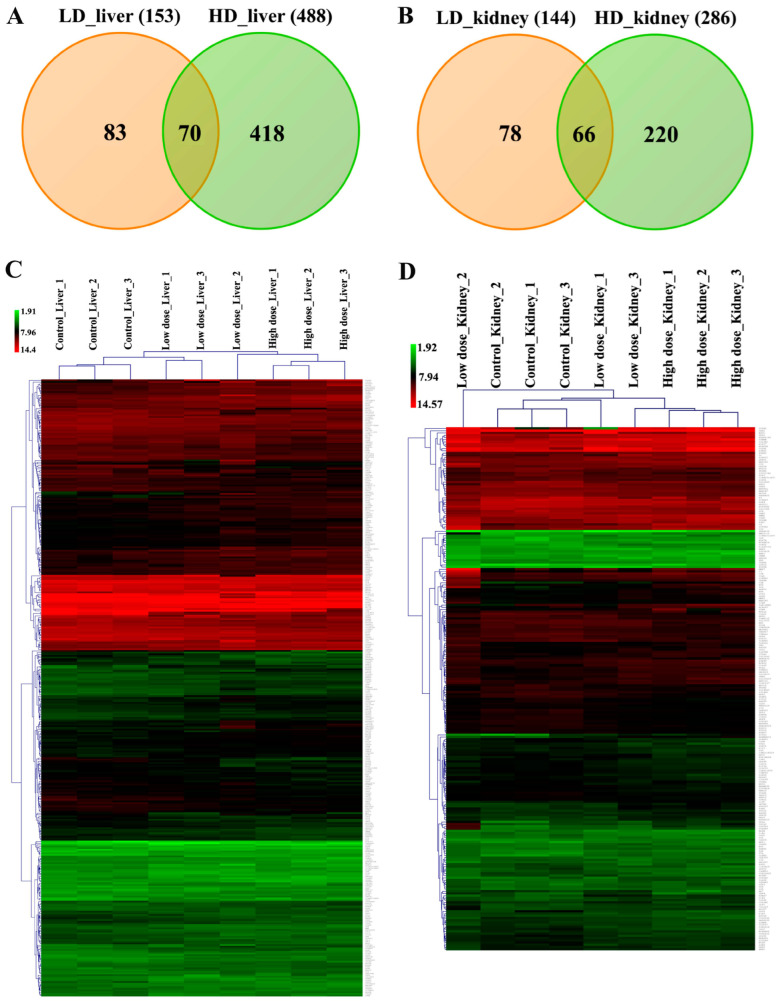
Differentially expressed genes after low- and high-dose diclofenac treatment. **Panel A**: Venn diagram of liver DEGs after low- (3 mg/kg/day) and high-dose (15 mg/kg/day) treatment. A total of 70 genes are regulated in common. **Panel B**: Venn diagram of kidney DEGs and a total of 144 and 286 genes were significantly regulated in low- and high-dose treatments, respectively, of which 66 were commonly regulated. **Panel C:** Shown is the average linkage hierarchical gene clustering of hepatic DEGs with Euclidean distance as default. The signal intensity values of regulated DEGs are depicted in the heat map. The low- and high-dose treatment groups are clearly separated from the controls. **Panel D:** Shown is the average linkage hierarchical gene clustering of kidney-related DEGs with Euclidean distance as default. The signal intensity values of regulated DEGs are depicted in the heatmap. The low- and high-dose treatment groups are clearly segregated from the controls.

Hepatic gene expression profiling of the low- and high-dose treatments defined 153 (71 up- and 82 down-) and 488 (234 up- and 254 down-) genes as significantly changed, of which 70 were commonly regulated (Figure 7A and Table 1). 

Similarly, 144 (59 up- and 85 down-) and 286 (125 up- and 161 down-) DEGs are regulated in the kidney in response to low- and high-dose diclofenac treatment and 66 DEGs are in common between the two regimens (Figure 7B and Table 2). 

We constructed heatmaps by applying the average linkage hierarchical clustering algorithm with Euclidian distance, and we showed the different treatment groups to be clearly segregated. The dendogram display clusters of genes regulated in common to imply dose-related genomic responses in the liver and kidney (Figure 7C,D). Table 1 and Table 2 provide information for organ-specific DEGs, and the data were categorized based on enriched biological processes.

### 2.7. Drug Metabolism and Transporters

We evaluated the expression of cytochrome P450 (CYP) monooxygenases and transporters. Specifically, the isoforms CYP3A22, CYP3A29, CYP3A39 and CYP3A46 were up to six-fold repressed, whereas CYP7A1, i.e., the key enzyme for bile acid synthesis, was reduced to 25% of the controls. Furthermore, we noted nearly three-fold changes in the coding of phase II drug metabolism genes, and this included glutathione and glucuronyl transferases (GSTA2, GSTT1, MGST3 and UGT1A6). Similarly, we determined a four-fold induction of GSTA1 and three-fold repressed GSTM3 expression in the kidneys of high-dose treated animals. Moreover, several transporters and key mitochondrial solute carriers were regulated in the liver and kidney (supplementary Table S1), and examples include the > 2-fold induced expression of SLC38A1, i.e., a glutamine transporter that is of critical importance in the detoxification of ammonia and SLC30A10, which selectively transports manganese. Conversely, SLC4A4 was about 60% repressed in the kidney; this transporter plays an essential role in bicarbonate homeostasis and intracellular pH regulation.

### 2.8. Functional Enrichment Analysis

We mapped about 93% of the genes to the human genome and considered orthologues for functional enrichment analysis. We categorized DEGs based on precompiled information available through the Gene Ontology Consortium, KEGG and BioCarta repositories, and by considering the statistical significance of enriched terms. The results imply the circadian clock, immune, inflammatory and stress responses, cell death and lipid metabolic process as being significantly changed in liver and kidney (Table 3 and Table 4). Supplementary Figures S5–S8 summarize the enriched ontology terms by considering biological processes, cellular components and molecular functions of DEGs in the liver and kidney.

Strikingly, diclofenac perturbed the core circadian clock and its associated nuclear receptor signaling pathways, i.e., ARNTL/BMAL1, NPAS2, CRY1, PER2, NR1D2/REV-ERB beta, DBP, DEC2 and RORC in the liver and kidney (Table 5). Importantly, emerging evidence suggests the suprachiasmatic nucleus harbors the master clock, and its primary task is to align metabolic functions in relation to the circadian rhythm. Diclofenac treatment caused stress that resulted in ACTH secretion and, via the hypothalamus-pituitary-adrenal (HPA) neuroendocrine axis, stimulated adrenal glucocorticoid synthesis. Testimony to increased ACTH secretion is the significant adrenal hypertrophy and thymic atrophy caused by excessive glucocorticoids in the systemic circulation (supplementary Figure S1). Furthermore, IHC confirmed increased hepatic glucocorticoid receptor (GR) activity, while the genomic data suggested GR-dependent gene regulation (Table 5). Therefore, we obtained evidence for diclofenac to disrupt the circadian rhythm with glucocorticoids affecting the liver clock to influence hepatic metabolism, immune and inflammatory responses. 

The genomic study also revealed repressed DNA damage and cell cycle arrest genes to support cell cycle progression. Prominent examples are cyclin G2 (CCNG2) and CDK inhibitor 1B (p27, KIP1), which typically augment cell cycle arrest. Similarly, the repression of the centromere protein F (CENPF), the telomeric repeat-binding factor (TERF1) and DNA topoisomerase 2 highlight treatment-related changes in the control of chromosome segregation, telomerase activity and the unwinding of double stranded DNA (supplementary Table S2). Additionally, the repression of the G2 checkpoint kinase WEE1 results in premature cell division and the production of smaller cells, whereas repression of the cell cycle regulator RGC32 dampens the immune response [39]. 

Conversely, diclofenac treatment induced hepatic cyclin-dependent kinase (CDK) inhibitor p21 (CDKN1A) and cyclin B1 kinase inhibitor GADD45G by two-fold to endorse cell cycle arrest. Drug treatment also caused an up to three-fold induced expression of pro-apoptotic signals in the kidney, i.e., CCAAT/enhancer-binding protein beta (C/EBPβ), acute phase protein S100A9 and L-selectin (SELE). In support of cellular defense, the histidine-rich glycoprotein (HRG) and the small heat shock protein family members CRYAB and HSP27 were induced by two-fold in the liver. Moreover, quinone 1 oxidoreductase (NQO1), i.e., an antioxidant defense enzyme, was three-fold induced in the kidney (supplementary Table S2).

Furthermore, the rate-limiting enzyme in bile acid synthesis, i.e., CYP7A1, and the liver receptor homologue 1 (NR5A2) were repressed by four- and two-fold. We observed a similar two-fold repressed expression for acyl-CoA oxidase (ACOX) which degrades C27-bile acid intermediates in peroxisomes, as well as the bile salt transporters ABCA8 and SLCO1B3 with critical roles in bile acid and bilirubin transport. Additionally, members of the gluconeogenesis pathway, e.g., glucose-6-phosphatase (G6PC), phosphoglycerate dehydrogenase (PHGDH), tyrosine aminotransferase (TAT) and PPARGC1A were repressed in expression by two-fold.

Besides, histopathology revealed diclofenac treatment to induce hepatic steatosis, and the genomic study informed the significant regulation of genes coding for lipogenesis, lipid transport, lipid droplet growth, ER stress and fatty acid oxidation (Table 6).

Thus, diclofenac treatment caused major changes in lipid homeostasis, and this included the lipid-droplet-associated PLIN2, ELOVL2, VLDLR and the ER-localized calreticulin (CALR), the calcium transporter ATP2A2, AGPAT9 and the cytoskeletal intermediate filament protein keratin 8 (KRT8) were induced up to five-fold. Conversely, the fatty acid synthase (FASN), mevalonate kinase (MVK) and the cholesterol transporter caveolin-1 (CAV1) were repressed by three-fold.

Among immune responses, the alternate pathway was regulated up to three-fold, as shown by the induced C7 and C9 expression changes in the liver. Moreover, the classical pathways, i.e., C1QA, C1QC, C1R, C1S, C3, C4A, CFB and CFH, were up-regulated by three-fold in the kidney after high-dose diclofenac treatment.

Pathway mapping revealed altered MAPK, interferon-γ and PPAR signaling in the liver. Additionally, members of the PI3K-AKT signaling pathway were regulated by two-fold in the liver and kidney (Table 3 and Table 4 and supplementary Table S2).

**Figure 8:**
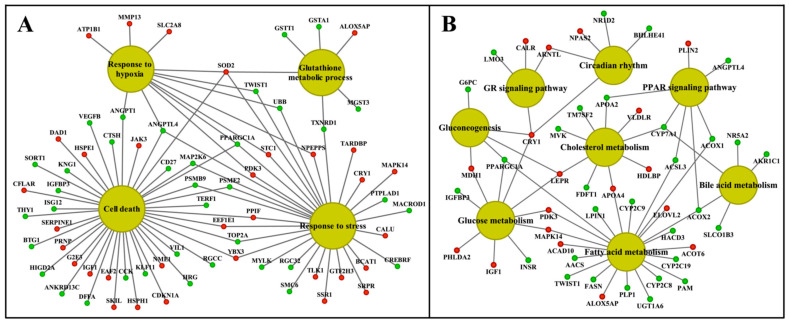
Gene ontology and pathway mapping network of liver-regulated genes in response to high-dose diclofenac treatment. The enriched biological processes and pathways of high-dose-treated animals were computed with the ClueGO and the GeneXplain software and visualized using the Cytoscape software version 3.9. **Panel A**: Stress and cell-death-associated gene network. **Panel B**: A network of genes enriched in circadian rhythm and metabolic processes. The red and green color nodes define induced and repressed transcript expression, respectively.

**Figure 9:**
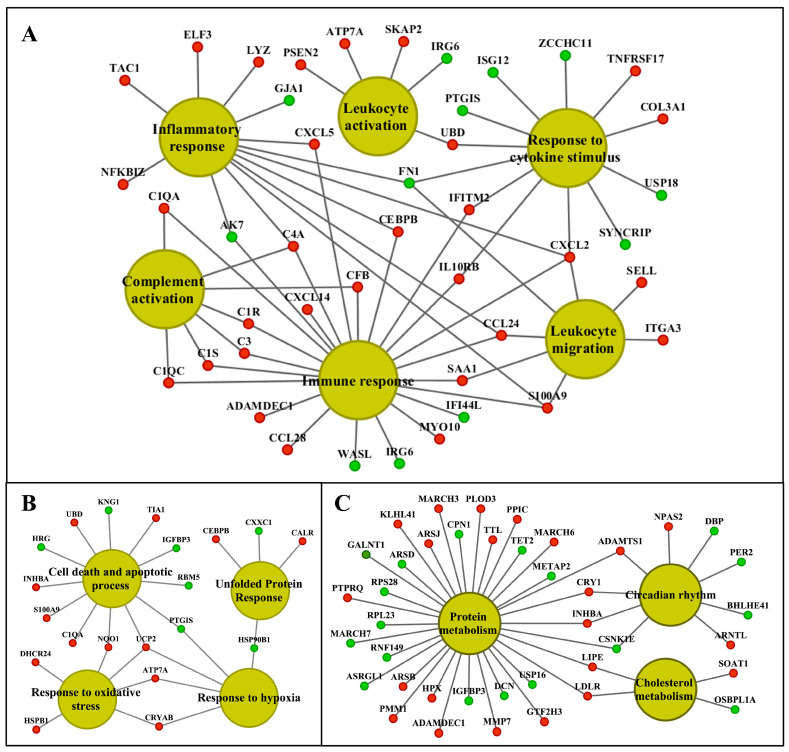
Gene ontology and pathway mapping network of kidney-regulated genes in response to high-dose diclofenac treatment. The visualization of kidney-enriched pathway terms and biological processes of high-dose-treated animals were computed with the ClueGO and the GeneXplain software. **Panel A:** A network of genes involved in immune and inflammatory responses. **Panel B:** Cellular stress and apoptosis-regulated genes and their network. **Panel C**: Genes associated with circadian rhythm and metabolic processes. The red and green color nodes illustrate up- and down-regulated genes, respectively.

We visualized the enriched GO terms with the ClueGO and the GeneXplain software (Figure 8 and Figure 9); the mapping of GO terms for the low-dose treatment group is given in supplementary Figure S9. Specifically, the ClueGO software grouped 125 hepatic DEGs into three distinct pathways, i.e., inflammation, circadian rhythm and metabolism, and we obtained similar results with the GeneXplain software. Here, we grouped 142 DEGs into these pathways. For the kidney, the ClueGO software assigned eighty-nine DEGs to seven major pathways, once again highlighting inflammation, response to cytokine signaling, complement cascades, wound healing and extracellular matrix, circadian rhythm, lipid and glucose metabolism, response to hypoxia, TGFß signaling and regulation of apoptotic processes. GO enrichment with the GeneXplain software produced almost identical results, and 119 DEGs were grouped into the respective pathways. Collectively, the consensus between the two different software demonstrates robustness of the findings, even though the number of DEGs mapped to GO terms differed between them.

### 2.9. Commonly Regulated Genes in Liver and Kidney

Diclofenac treatment caused organ-specific and tissue-independent genomic responses, with 43 genes being regulated in common (Table 7); the top-ranking biological pathways were immune and inflammatory responses and the circadian clock. Among commonly regulated genes in the liver and kidney are the pro-inflammatory CXCL2, LYZ and S100A9, which were up to seven-fold induced in both organs.

### 2.10. Molecular Networks in Liver and Kidney

We searched the STRING database version 10.5 for protein-protein interaction (PPI), and 60% (92 out of 153 genes) and 67% (325 out of 488 genes) of DEGs engaged in 217 and 885 interactions in the liver network after low- and high-dose treatments (supplementary Figure S10A,B). In the case of the kidney, 53% (76 out of 144 genes) and 63% (180 out of 286 genes) of DEGs function in 105 and 412 PPI, respectively (supplementary Figure S11A,B).

### 2.11. Master Regulators and Their Associated Networks

Shown in Table 8 are master regulators and their regulation in the liver and kidney in response to diclofenac treatment. Except for cryptochrome 1 (CRY1) and the lipopolysaccharide-induced TNF factor (LITAF), the majority of master regulators were significantly repressed in expression. Furthermore, we identified CRY1 as a master regulator in low- and high-dose diclofenac treatments. Likewise, kidney genomic responses revealed matrix metallopeptidase 7 (MMP7), complement component 1 (C1QA) and nuclear factor of kappa light polypeptide gene enhancer in B cells inhibitor, zeta (NFKBIZ) as significantly induced, while another four master regulators were repressed in expression.

The computational analysis defined the circadian clock gene CRY1, hypoxia inducible factor 1, alpha subunit (HIF1A) and nuclear receptor subfamily 3, group C, member 1 (NR3C1) as master regulators in the liver and their associated networks involved 27, 39 and 42 DEGs after low-dose diclofenac treatment. Similarly, high-dose diclofenac networks involved 66, 111, 111 and 77 DEGs with CRY1, insulin-like growth factor binding protein 2 (IGFBP2), LITAF and angiopoietin-like 4 (ANGPTL4) as master regulators. 

Additionally, dipeptidyl-peptidase 4 (DPP4), which inactivates glucose-like peptidase 1 and therefore insulin secretion, angiopoietin 1 (ANGPT1) and sirtuin 1 (SIRT1); i.e., NAD-dependent deacetylase are key regulators in the kidney after low-dose diclofenac treatment and encompassed 40, 25 and 38 DEGs in their networks, respectively. The high-dose diclofenac treatment revealed MMP-7, C1QA, NFKBIZ and insulin-like growth factor binding protein 3 (IGFBP3) in the kidney as master regulators, and the associated networks consisted of 52, 49, 56 and 56 of DEGs, respectively.

**Figure 10:**
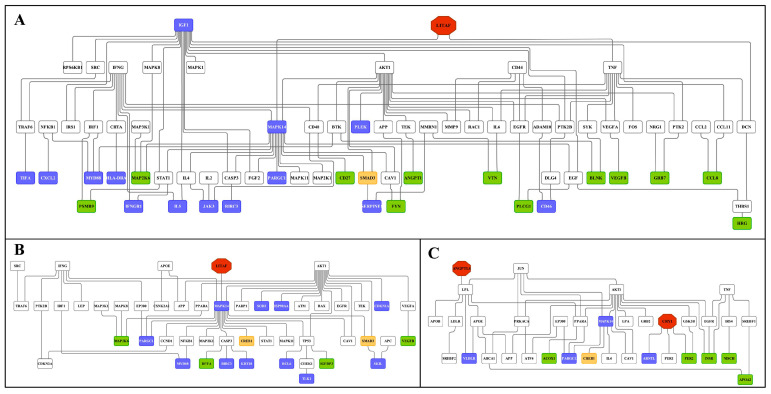
Master regulatory gene networks in the liver of high-dose diclofenac-treated animals. Based on interaction information available in the GeneWays database, master regulatory gene networks were constructed and fused using the GeneXplain platform. The red, violet, green, white and yellow nodes represent genes coding for master regulators, up-, down-regulated DEGs, connecting genes and enriched transcription factors, respectively. **Panel A:** Master regulatory network of immune and inflammatory response genes. **Panel B:** Master regulatory network of cellular stress and apoptosis-regulated genes. **Panel C**: Master regulatory network of genes involved in metabolic processes.

Subsequently we constructed an integrated master regulator network and the fused liver networks comprised 29% (44 out of 153 genes) and 24% (119 out of 488) of DEGs after low- and high-dose diclofenac treatments (Figure 10 and supplementary Figure S12A).

**Figure 11:**
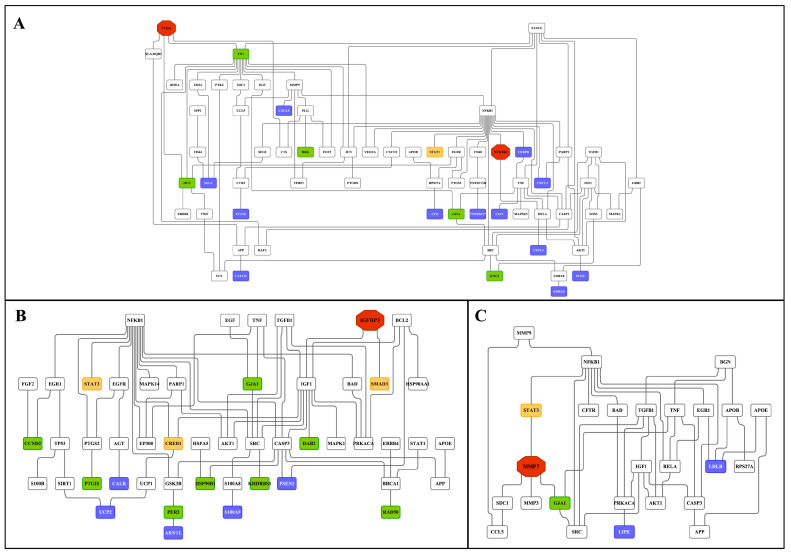
Master regulatory gene networks in the kidney of high-dose diclofenac-treated animals. Based on interaction information available in the GeneWays database, the master regulatory gene networks were constructed and fused using the GeneXplain platform. The red, violet, green, white and yellow nodes represent genes coding for master regulators, up-, down-regulated DEGs, connecting genes and enriched transcription factors, respectively. **Panel A:** Master regulatory network of immune and inflammatory response genes. **Panel B:** Master regulatory network of cellular stress and apoptosis-regulated genes. **Panel C**: Master regulatory network of genes involved in metabolic processes.

Similarly, 28% (41 out of 144) and 20% (58 out of 286) of DEGs were regulated in the kidney in response to low- and high-dose treatments (Figure 11 and supplementary Figure S12B). Intriguingly, we confirmed the previously reported diclofenac liver master regulators for mice and dogs, i.e., CD44, LEPR and THBS1, in the fused networks in minipigs, and the high-dose kidney network also contained CD44, S100A8 and selectin E.

### 2.12. Enriched Transcription Factor Binding Sites for the Liver Clock

**Figure 12:**
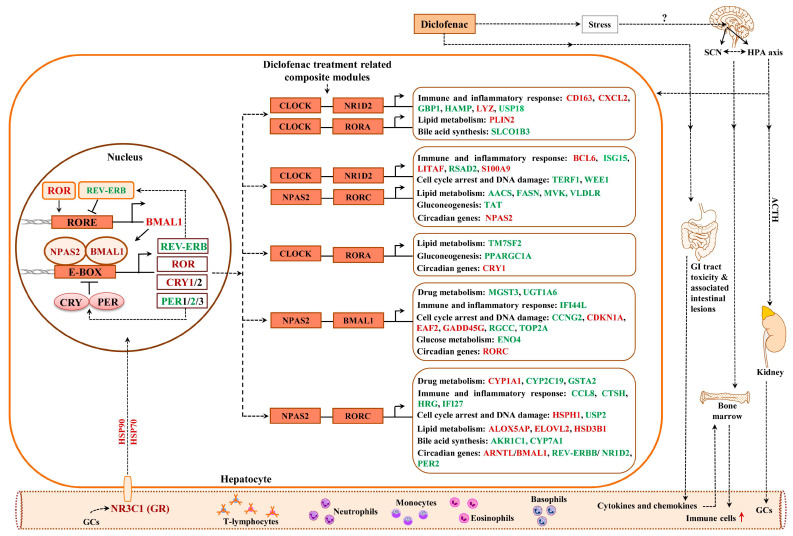
Liver clock regulatory gene network in response to diclofenac treatment. Drug treatment resulted in perturbed liver clock activity. Shown are composite modules of circadian transcriptional regulators at promoters of hepatic DEGs coding for immune response, inflammation, metabolism, cell cycle arrest and DNA damage. Genes marked as red are significantly up-regulated; those given in green are repressed in expression. SCN = suprachiasmatic nucleus, ACTH = adrenocorticotropic hormone, GC = glucocorticoids, HPA axis = hypothalamic pituitary adrenal axis.

Figure 12 depicts the liver clock and its target genes. The core and auxiliary loops of the circadian clock consist of several transcription factors, which interact with E-box and RORE binding sites in the promoters of DEGs. Initially, we considered the number of TFBS in promoters of regulated genes for the core circadian transcription factors, i.e., BMAL1, CLOCK, NPAS2, REV-ERBA, REV-ERBß and RORC, in addition to transcription factors which participate in liver clock oscillation, i.e., GR, DBP, FXR, HNF4A, HNF6, PPARA and DEC2. For instance, the transcription factor DEC2 acts as a transcriptional repressor of orexin, i.e., a neuropeptide of the circadian clock, and, although nearly three-fold repressed, did not influence the expression of orexin. This neuropeptide connects the hypothalamus with the liver via hypocretin signals to influence liver metabolism [40], and an immunoprecipitation study revealed DEC2 to interact with E12 and MyoD1 on the prepro-orexin gene promoter [41].

Together, we observed significant differences in transcriptional responses to BMAL1, CLOCK, REV-ERBA and DEC2 targeted promoters (supplementary Figure S13 panel C) with expression of genes coding for glucose, lipid and bile acid metabolism being influenced by the number of TFBS, i.e., a reduced number of TFBS was significantly linked to repressed expression of target genes. However, with HNF6, an increased number of binding sites was associated with repressed DEGs. Supplementary Figure S14 displays the average number of TFBS obtained for the different PWMs. The computational analysis suggested the number of TFBS to influence the transcriptional responses to BMAL1, CLOCK, REV-ERBA, DEC2 and HNF6 (supplementary Figure S13). We found 77% of DEGs to contain enriched TFBS for components of the liver clock, i.e., BMAL1, CLOCK, NPAS2 and GR, in response to diclofenac treatment.

### 2.13. Composite Modules of the Circadian Clock and Glucocorticoid Receptor 

Given the significant enrichment of TFBS for liver clock components in DEGs (supplementary Table S3) and to increase the specificity of our findings, we considered the co-occupancy of several enriched TFBS at gene-specific promoters. We examined liver-clock-associated transcription factors and considered the number of TFBS acting on E-box motives as heterodimeric complexes.

**Figure 13:**
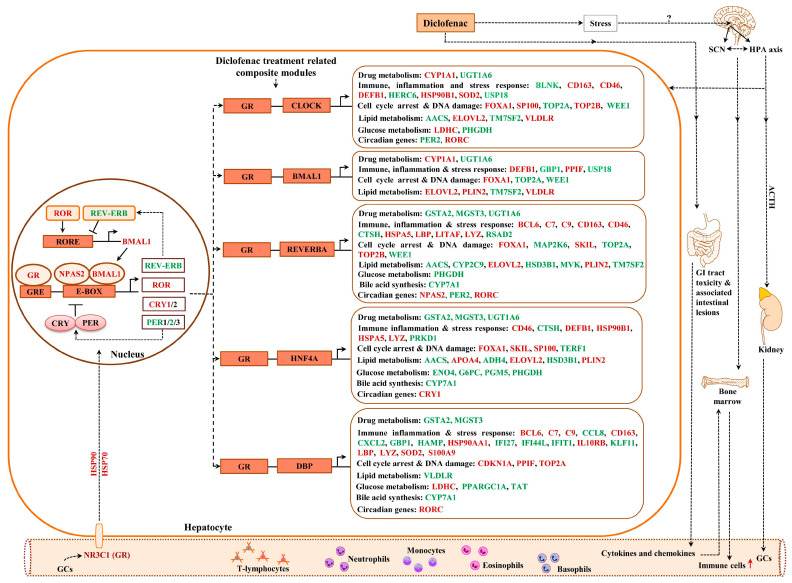
Liver clock and glucocorticoid receptor co-regulated genes in response to diclofenac treatment. Shown are composite modules of circadian transcriptional regulators and the glucocorticoid receptor at the gene-specific promoters of hepatic DEGs coding for immune response, inflammation, metabolism, cell cycle arrest and DNA damage. Genes marked as red are significantly up-regulated; those given in green are repressed in expression.

Shown in Figure 12 and Figure 13 are composite modules of enriched liver clock and glucocorticoid-receptor-regulated genes in response to diclofenac treatment. The composite modules were constructed based on the significant enrichment of CLOCK-NR1D2, NPAS2-BMAL1, CLOCK-RORA and NPAS2-RORC heterodimeric complexes acting on gene-specific promoters of immune, stress, inflammation, hypoxia, acute-phase response, oxidation-reduction and cell-death-coding genes (Figure 12). Strikingly, 82% of DEGs were enriched for the liver clock heterodimeric complexes, notably CLOCK-NRD1D2, CLOCK-RORA, NPAS2-RORC and NPAS2-BMAL1.

We used the same strategy to investigate liver clock and glucocorticoid receptor interactions (Figure 13) and, for 64 % of DEGs, the composite modules consisting of the liver clock and the glucocorticoid receptor were significantly enriched, notably GR-BMAL1, GR-CLOCK and GR-ReverbA (see Z-score, supplementary Table S4).

### 2.14. GR Signaling

**Figure 14:**
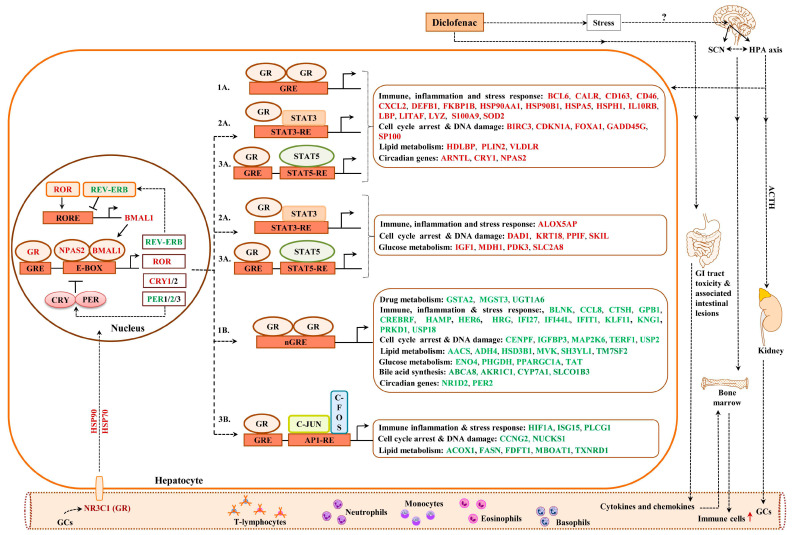
Glucocorticoid receptor targeted genes in response to diclofenac treatment. The glucocorticoid receptor can influence gene expression either through direct or indirect mechanisms. The direct, tethering and/or composite model of GR activity as originally proposed by Oakley and Cidlowski (50) was employed; 1a = direct activation, 1b = direct repression, 2a = tethering model, 3a = indirect or transactivation composite model, 3b = indirect or transrepression composite model.

We have already emphasized the importance of the glucocorticoid receptor signaling pathway and the circadian clock (see above) in genomic responses to diclofenac treatment. Now, we consider glucocorticoid receptor activities independent of the liver clock. Oakley and Cidlowski [42] proposed a model of direct, tethering or composite GR activity that results in activated or repressed transcriptional responses. We applied the proposed rules and, in the case of the direct model, juxtaposed GR binding sites in addition to the coactivators or corepressors that are required (Figure 14).

Based on such rules, all 214 up-regulated DEGs fulfilled such criteria, while, for 231 repressed DEGs, a total of 175 or 76% agreed with the set rule (supplementary Table S5). Similarly, the tethering model of up-regulated DEGs relies on juxtaposing STAT3 binding sites to interact with the GR receptor. Here, we compared a set of 600 randomly chosen non-regulated genes with diclofenac-regulated genes and found 171 or 80% of up-regulated DEGs to fulfill this criterion. Conversely, for repressed DEGs, the tethering model requires interaction of the GR with NFKB binding sites; however, only four DEGs fulfilled such criteria. Finally, we evaluated the composite model that is composed of GR and STAT5 recognition sites. Once again, 207 out of 214 up-regulated DEGs or 97% fulfilled this requirement. In the case of transcriptional repression, the composite model foresees the interaction of GR with AP binding sites, but only 35 genes qualified (supplementary Table S5). We compared the findings of the different models, and, for up-regulated genes, the different models produced almost identical results; i.e., 172 genes or 81% of GR-targeted genes were in common. However, for down-regulated genes, there were no significant differences between the DEGs qualifying for the GR tethering model, and only three DEGs overlapped in the GR direct and GR composite models (supplementary Figure S15). 

### 2.15. Immunohistochemistry Confirms Regulation of Liver Clock Components

A complex relationship exists between the circadian clock and inflammation [43,44,45], and, in the present study, nearly 80% of DEGs contained enriched TFBS for components of the liver clock, i.e., BMAL1, CLOCK, NPAS2 and GR. Indeed, PER1/2 and CRY1/2 are clock target genes and, through a negative feedback loop, both proteins repress their own expression by blocking the activity of the BMAL-CLOCK heterodimeric TF complex on E-box motives of targeted promoters [46]. 

**Figure 15:**
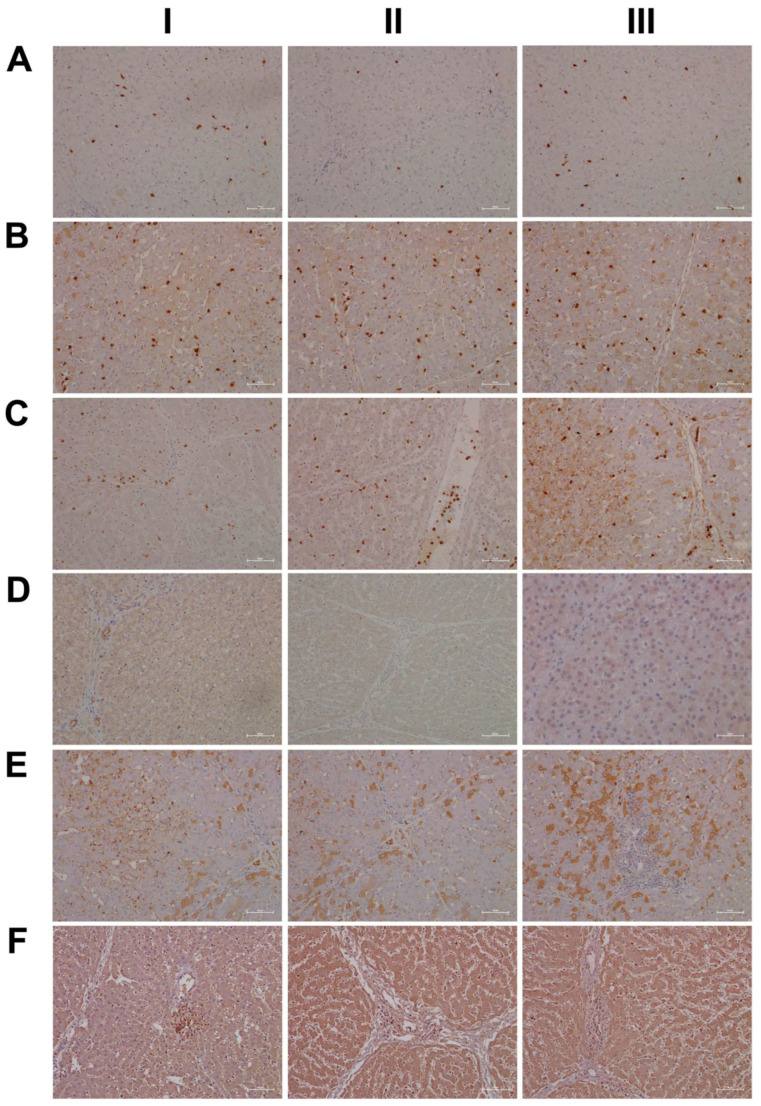
Immunohistochemistry of the liver clock components Per2 and CRY1 in liver sections of control and diclofenac-treated animals after daily dosing for 28 days. A complex relationship exists between the circadian clock and inflammation. **Panel AI–III:** Liver sections of three individual control animals: Disseminated throughout the liver lobule are Per2-positive macrophages. **Panel BI–III:** Diclofenac low-dose treatment caused a marked increase in activated Per2-positive macrophages. Some hepatocytes and sinusoidal endothelium also stained positive. **Panel CI–III**: High-dose diclofenac treatment. Hepatocytes of an inflamed liver lobule abundantly express Per2 (CIII). **Panel DI–III**: Liver sections of three individual control animals. In one control (DI) bile duct epithelium appeared slightly positive; none of the controls expressed the protein (DI–III). **Panel EI–III**: Diclofenac low-dose treatment. Shown in panel EI is the liver section of a low-dose-treated animal with marked CRY1-positive macrophage infiltrates within an inflamed hepatic lobule. Conversely, EIII illustrates marked portal histiocytic infiltrates of monocytes and macrophages; however, none express CRY1, while panel F1 documents CRY-positive histiocytic infiltrates forming a granuloma adjacent to the central vein of an inflamed liver lobule. **Panel FI–III**: High-dose diclofenac treatment. We observed a clear dose-related increase in CRY1 expression.

Shown in Figure 15 (panel AI–III) are representative images of liver sections from three individual control animals, and disseminated throughout the liver lobule are Per2-positive macrophages. Diclofenac low-dose treatment caused a marked increase in activated Per2-positive macrophages and some hepatocytes, as well as sinusoidal endothelium, stained positive as well (panel BI–III). We did not observe dose-related changes in Per2 expression (panel CI–III); notwithstanding hepatocytes of an inflamed liver lobule expressed Per2 abundantly (CIII). It is of considerable importance that, with high-dose-treated animals, Per2 transcription was nearly three-fold repressed, and this suggests an activated negative feedback loop. Furthermore, Per1 attenuated excessive immune responses in an LPS model of liver injury by dampening Kupffer cell recruitment, whereas Per1 deletion caused a remarkable increase in pro-inflammatory macrophages [47].

Then, we considered cryptochrome 1 expression in the control and diclofenac-treated animals (panels D-F) and, in except of one control animal (DI) where the bile duct epithelium appeared slightly positive, none of the controls expressed the protein (DI–III). In strong contrast, we observed a clear dose-related increase in the hepatic expression of CRY1 (E-F). Shown in panel EI is the liver section of a low-dose-treated animal with marked CRY1-positive macrophage infiltrates within an inflamed hepatic lobule. Conversely, EIII illustrates marked portal histiocytic infiltrates of monocytes and macrophages; however, none express CRY1, while panel F1 documents CRY-positive histiocytic infiltrates forming a granuloma adjacent to the central vein of an inflamed liver lobule. Note an earlier study demonstrated the importance of CRY1 in the regulation of pro-inflammatory cytokines, and Cry-deficient macrophages are hypersensitive to immune responses [48]. We found CRY1mRNA > four-fold up-regulated in diclofenac-treated minipigs, and immunohistochemistry evidenced its induced expression, particularly in zones of inflammation. Importantly, CRY1 reduces TNFα- and NFkB-mediated inflammatory responses [49], and the genomic study provided evidence for the repressed expression of interferon gamma response genes and their associated signaling pathway (supplementary Table S2).

Another core protein of the circadian machinery is CLOCK, and this basic helix-loop-helix PAS domain transcription factor functions together with ARNTL (BMAL1) in the control of circadian-regulated genes, such as CRY1, PER1/2/3, REV-Erb, ROR, etc. Interestingly, CLOCK null mice display a normal phenotype, presumably due to the fact that CLOCK can be substituted by the PAS domain protein NPAS2 [50]. Indeed, we found NPAS2 nearly three-fold induced upon diclofenac treatment. Additionally, CLOCK stimulated Histone 3 and 4 acetylation, thereby enabling gene transcription and, through physical interactions with the glucocorticoid receptor (GR), reduced its transcriptional responses, while its interaction with the Rel protein p65 augmented expression of pro-inflammatory molecules [44]. CLOCK is a positive regulator of NFκB-mediated transcription [51], and, given that CLOCK and BMAL1 function together, the dysfunction of these proteins resulted in distinct physiological phenotypes [50]. This included an impaired detoxification of drugs [52]. In fact, the assembly of different bHLH-PAS heterodimeric complexes leads to subtle differences in their interaction with DNA to initiate distinct transcriptional programs in the control of circadian rhythm, immune-, hypoxia- and drug responses [53].

**Figure 16:**
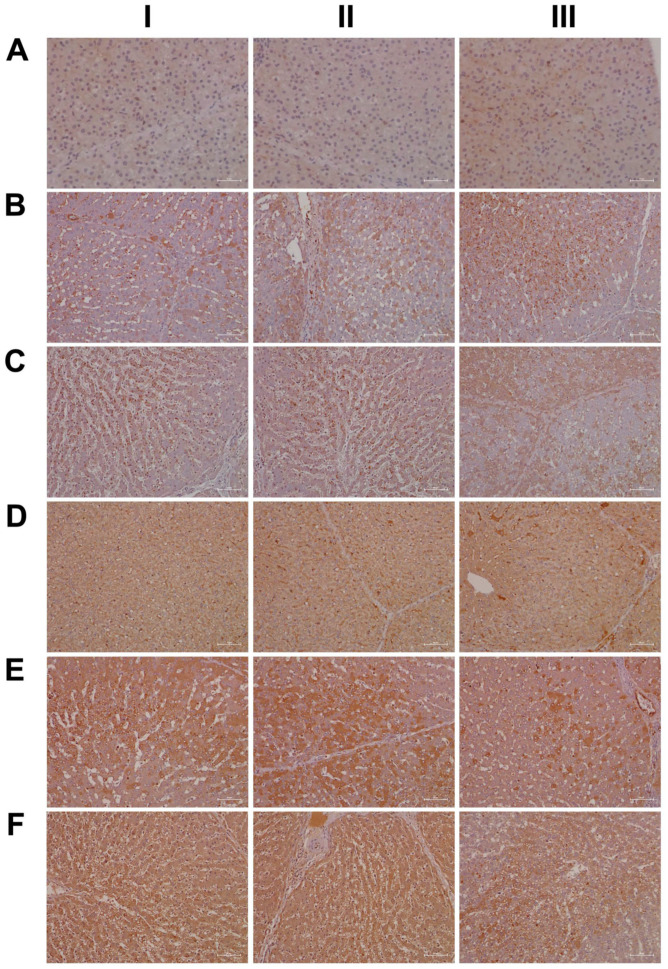
Immunohistochemistry of the liver clock components CLOCK and CYP1A1 in liver sections of control and diclofenac-treated animals after daily dosing for 28 days. CLOCK is a transcription factor and, together with BMAL1, forms a heterodimer and binds to E-Box in the promoter of Per1/2 and Cry1/2 to augment their expression. Furthermore, the aryl hydrocarbon receptor (AhR) senses exposure to foreign chemicals, including drugs, and forms functional complexes with the bHLH PAS domain protein ARNT to control the expression of genes coding for xenobiotic defense, such as CYP1A1 and CYP1B1. **Panel AI–III:** Liver sections of three individual control animals: Except for panel AIII, where a few hepatocytes and very rarely macrophages stained positive, none of the controls expressed the CLOCK protein. **Panel BI–III:** Diclofenac low-dose treatment caused marked cytosolic and nuclear CLOCK expression. Depicted in BIII is an inflamed hepatic lobule with marked CLOCK-positive macrophage and monocytic infiltrates. **Panel CI–III:** High-dose diclofenac treatment. Most macrophages stained positive for CLOCK (CI–II), and its expression did not apparently follow the zonation of hepatocytes (CIII). **Panel DI–III:** Liver sections of three individual controls. Note the slight to moderate cytosolic staining of hepatocytes, even though individual hepatocytes displayed marked expression of this protein (DIII). The sinusoids are demarcated by positive CYP1A1 staining. Very rarely, resident Kupffer cells expressed CYP1A1. **Panel EI–III:** Diclofenac low-dose treatment. Depicted in panel EII–III is a mosaic-like expression pattern of hepatocytes with obvious expression of the CYP1A1 protein and marked CYP1A1 endothelial expression in a portal triad (EIII). Within inflamed lobules, hepatocytes expressed less CYP1A1 (EI&EIII). **Panel FI–III:** High-dose diclofenac treatment. We did not observe dose-related changes in CYP1A1 expression, and inflamed lobules expressed less CYP1A1 (FI–III). Notwithstanding, the number of CYP1A1-positive macrophages significantly increased (FIII).

As shown in Figure 16A, and except for panel AIII where a few hepatocytes and very rarely macrophages stained positive, none of the controls expressed the CLOCK protein. In strong contrast, CLOCK expression was markedly increased following low- (panel B) and high-dose diclofenac treatments. We did not observe dose-related changes in the hepatic expression of CLOCK; however, we obtained clear evidence for its abundant cytosolic expression. Furthermore, we rarely observed nuclear CLOCK staining (BI–III). Depicted in BIII is an inflamed hepatic lobule with marked CLOCK-positive macrophage and monocytic infiltrates. Similarly, at the high diclofenac dose, most macrophages stained positive for CLOCK (CI–II), and apparently its expression did not follow the zonation of hepatocytes (CIII). 

As previously mentioned, the aryl hydrocarbon receptor (AhR) senses exposure to foreign chemicals, including drugs, and forms functional complexes with the bHLH PAS domain protein ARNT to control the expression of genes coding for xenobiotic metabolism, such as CYP1A1 and CYP1B1. Although diclofenac treatment did not induce gene transcription of AhR and/or ARNT itself, we observed a marked induction of CYP1A1. Shown in Figure 16, panel DI–III, are liver sections of three individual control animals. Note the slight to moderate cytosolic staining of hepatocytes, even though individual hepatocytes displayed marked expression of this protein (DIII). The sinusoids were demarcated by the positive CYP1A1 staining, and we infer sinusoidal endothelium to be positive as well. Very rarely, resident Kupffer cells expressed CYP1A1. Shown in panel E and F are low- and high-dose diclofenac-treated animals, and depicted in panel EII–III is a mosaic-like expression pattern of hepatocytes with obvious expression of the CYP1A1 protein. We observed marked CYP1A1 endothelial expression in a portal triad (EIII). Within inflamed lobules, hepatocytes expressed less CYP1A1 (EI&EIII). Remarkably, the number of CYP1A1-positive macrophages increased, and there is evidence for CYP1A1 to enhance the inflammatory response of macrophages [54]. We did not observe dose-related changes in CYP1A1 expression, and, once again, hepatocytes of inflamed lobules expressed less CYP1A1 (FI–III). Notwithstanding, the number of CYP1A1-positive macrophages significantly increased (FIII).

We have already addressed the delicate interplay of glucocorticoids and the circadian clock [55], and cortisol is a key player in the anti-inflammatory response. Nonetheless, cortisol also functions as a stress hormone, and chronic exposure to cortisol facilitates the production of inflammatory cytokines [56]. In fact, the liver is a major site for cholesterol biosynthesis, and cholesterol is the major building block for steroid hormones. Although cortisol is mainly produced in the zona fasciculata of the adrenal cortex, the majority of circulating cortisol stems from cholesterol bound to liver-secreted high-density lipoproteins (HDL) which is delivered to the adrenal gland. In the circulation, cortisol is primarily (80%) transported by the corticosteroid binding globulin (CBG), and this protein is mainly synthesized in the liver. However, only free cortisol binds to the glucocorticoid receptor (GR), which typically accounts for 5% of cortisol in the circulation. Free cortisol diffuses across the cell membrane and binds to the GR, whose expression has been reported for basically all cells [57]. Cortisol release from CBG requires the activity of the neutrophil enolase, which cleaves the so-called reactive center loop of CBG and thereby releases cortisol [57]. Importantly, diclofenac treatment caused a marked increase in neutrophil count from day 8 onwards (see above), and histopathology confirmed neutrophilic infiltrates into regions of harmed hepatocytes (Figure 6). Upon ligand (cortisol) activation, the cytosolic GR receptor complex translocates to the nucleus [42,58]. GR is bound to a multiprotein complex, and research has demonstrated GR to continuously shuttle between the nucleus and the cytoplasm in the presence and absence of its ligand [58,59].

**Figure 17:**
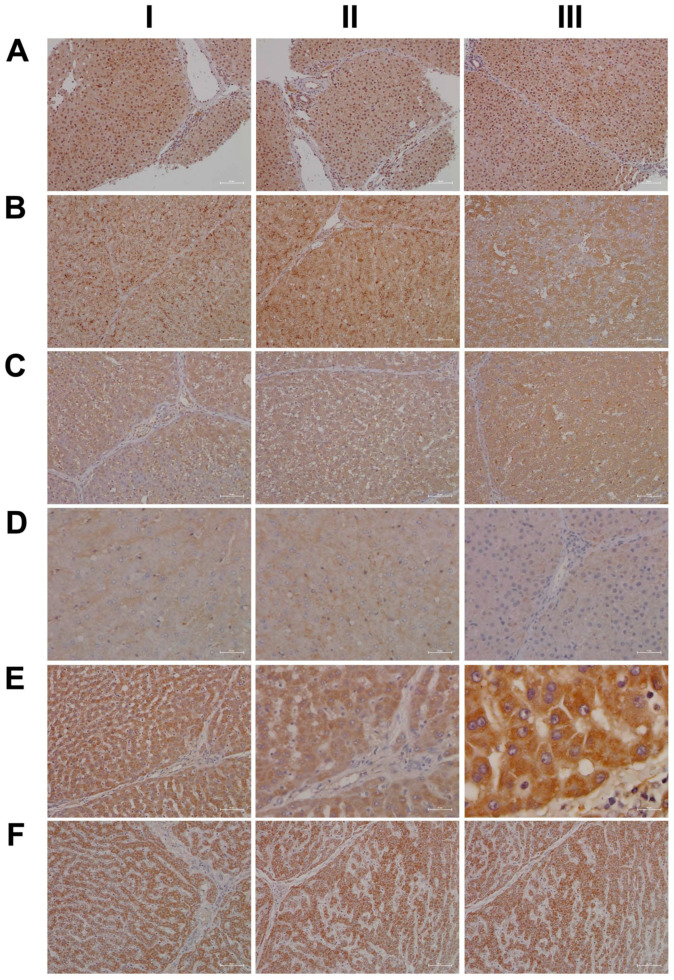
Immunohistochemistry of corticosteroid binding globulin and the glucocorticoid receptor in liver sections of control and diclofenac-treated animals after daily dosing for 28 days. In the circulation, cortisol is primarily transported by the corticosteroid binding globulin (CBG), and this protein is mainly synthesized in the liver. Only free cortisol binds to the glucocorticoid receptor (GR) and diffuses across the cell membrane and binds to the GR. **Panel AI–III:** Liver sections of three individual control animals. We observed slight to moderate cytosolic and nuclear staining of CBG. **Panel BI–III:** Diclofenac low-dose treatment caused marked increases in CBG, with a dust-like appearance in the sinusoids of treated animals. Additionally, BIII exemplifies the mosaic-like CBG expression pattern with harmed hepatocytes less capable of its synthesis. **Panel CI–III**: High-dose diclofenac treatment. Hepatocytic CBG expression was significantly reduced; this results in increased pool size of unbound/free and, therefore, biologically active cortisol. The high-dose regimen harmed hepatocytes severely and some failed to synthesize CBG (CI–II). Note the CBG-positive macrophages (CIII), which suggests CBG directly interacts with the glucocorticoid receptor of sinusoidal macrophages to dampen their pro-inflammatory activity. **Panel DI–III:** Liver sections of three individual control animals. We noted a faint cytosolic and, in part, sinusoidal expression of GR. **Panel EI–III:** Diclofenac low-dose treatment caused marked increases in cytosolic GR expression, which was independent of the dose (panels **E**,**F**). We did not observe nuclear GR staining, as exemplified in an HPV of a low-dose-treated animal (EIII). **Panel FI–III:** High-dose diclofenac treatment. We did not observe dose-related changes in GR expression, which implies the lower dose to elicit a maximum response.

Shown in Figure 17 (AI–III) are liver sections from three individual control animals. We observed slight to moderate cytosolic and nuclear staining of CBG. Based on these findings, it is tempting to speculate that CBG functions in the nuclear trafficking of proteins other than cortisol [57]. Low-dose diclofenac treatment (BI–II) caused marked increases in CBG with a dust-like appearance in the sinusoids of treated animals. Additionally, BIII exemplifies the mosaic-like CBG expression pattern with harmed hepatocytes less capable of its synthesis. At the high diclofenac dose (CI–III), CBG expression is significantly reduced, and this will increase the pool size of unbound/free and, therefore, biologically active cortisol. Only free cortisol binds to the glucocorticoid receptor (GR) and augments its activity. We observed marked GR expression in diclofenac-treated animals, as described below. Nonetheless, the high-dose regimen caused severely harmed hepatocytes, some of which failed to synthesize CBG (CI–II). Moreover, we observed CBG-positive macrophages (CIII), and this suggests CBG directly interacts with the GR of sinusoidal macrophages as to dampen their pro-inflammatory activity.

Based on the CBG findings, we were interested in investigating the regulation of the glucocorticoid receptor; depicted in DI–III are the liver sections of three individual control animals. For the controls, we noted a faint cytosolic and, in part, sinusoidal expression of GR. Strikingly, diclofenac treatment caused marked increases in cytosolic GR expression, which was independent of the dose (panels E–F). However, we did not observe nuclear GR staining as exemplified in an HPV of a low-dose-treated animal (EIII).

**Figure 18:**
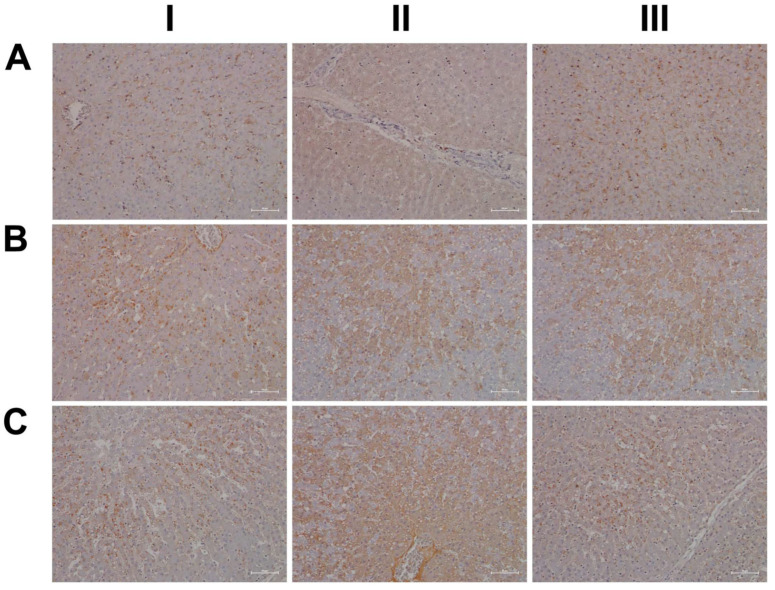
Immunohistochemistry of the corticotropin-releasing hormone receptor in liver sections of control and diclofenac-treated animals after daily dosing for 28 days. For its important role in stress signaling, we investigated hepatic expression of CRF1, which is the receptor of the corticotropin-releasing factor (CRF), and the CRF neuropeptide plays an important role in liver pathology. **Panel AI–III:** Liver sections of three individual control animals. We observed faint sinusoidal and, very rarely, CRF-positive macrophages. **Panel BI–III:** Diclofenac low-dose treatment. CRF1 activation of macrophages promotes their M1 polarization, and we observed marked increases in CRF-positive macrophages in low (BI)-treated animals. Furthermore, vascular endothelial cells stained positive (BI, CII), and we observed cytosolic expression of the receptor among liver cells of inflamed liver lobules (BII–III, CII). However, not all cells expressed the CRF receptor, and we speculate regenerating hepatocytes to express CRF1 more abundantly to support anti-inflammatory and anti-apoptotic reactions. **Panel CI–III:** Diclofenac high-dose treatment. Marked increase in CRF-positive macrophages (CI, CIII) with induced cytosolic expression of the receptor among liver cells of inflamed liver lobules (CII). Sinusoidal endothelial cells stain positive (CII).

Finally, due to its important role in stress signaling, we investigated the hepatic expression of CRF1, which is the receptor of the corticotropin-releasing factor (CRF). Importantly, CRF affects cortisol synthesis via the hypothalamo-hypophyseal portal system, where it stimulates the release of adrenocorticotropic hormone (ACTH). Subsequently, ACTH stimulates cortical cells of the adrenal gland to produce cortisol [60]. Notwithstanding, the CRF neuropeptide also plays an important role in liver pathology [61]. Shown in Figure 18 are liver sections of three control animals (AI–III), and we observed faint sinusoidal and, very rarely, CRF-positive macrophages. Recent evidence has suggested CRF1 activation of macrophages to promote their M1 polarization [62], and we observed marked increases in CRF-positive macrophages in low- (BI) and high-dose (CI, CIII)-treated animals. Furthermore, we found vascular endothelial cells to stain positive following diclofenac treatment (BI, CII) and obtained evidence for a dose unrelated cytosolic expression among liver cells of inflamed liver lobules (BII–III, CII). However, not all cells expressed the CRF receptor, and we speculate regenerating hepatocytes to express CRF1 more abundantly to support anti-inflammatory and anti-apoptotic reactions.

### 2.16. Regulatory Gene Networks

To gain insight into gene regulatory networks that are independent of the liver clock and glucocorticoid receptor, we searched for enriched transcription-factor-binding sites in promoters of DEGs (supplementary Table S3). This revealed 198 and 145 TFBS in promoter sequences of liver- and kidney-regulated genes. Importantly, the myocyte enhancer factor-2 (MEF2) and the AT-rich interactive domain 5A (ARID5A) were significantly enriched binding sites in promoters of low-dose diclofenac-regulated genes (supplementary Figure S16A), whereas glucocorticoid response elements (GRE) and Krüppel-like family transcription factor (KLF6) were significantly enriched in promoters of DEGs after high-dose treatments (supplementary Figure S16B). For the kidney, the composite module of the low-dose treatment group consisted of proto-oncogene 1, transcription factor (ETS1) and zinc finger protein 217 (ZNF217) (supplementary Figure S17A), whereas for the high-dose treatment the composite module consisted of an amino acid response element, ATF4 binding site (AARE), *GATA* binding factors (GATA) and SMAD family transcription factors (supplementary Figure S17B). Altogether, 195 DEGs or 34% are candidates for these independent regulatory gene networks. 

### 2.17. Validation of Transcriptional Responses by RT-qPCR

**Figure 19:**
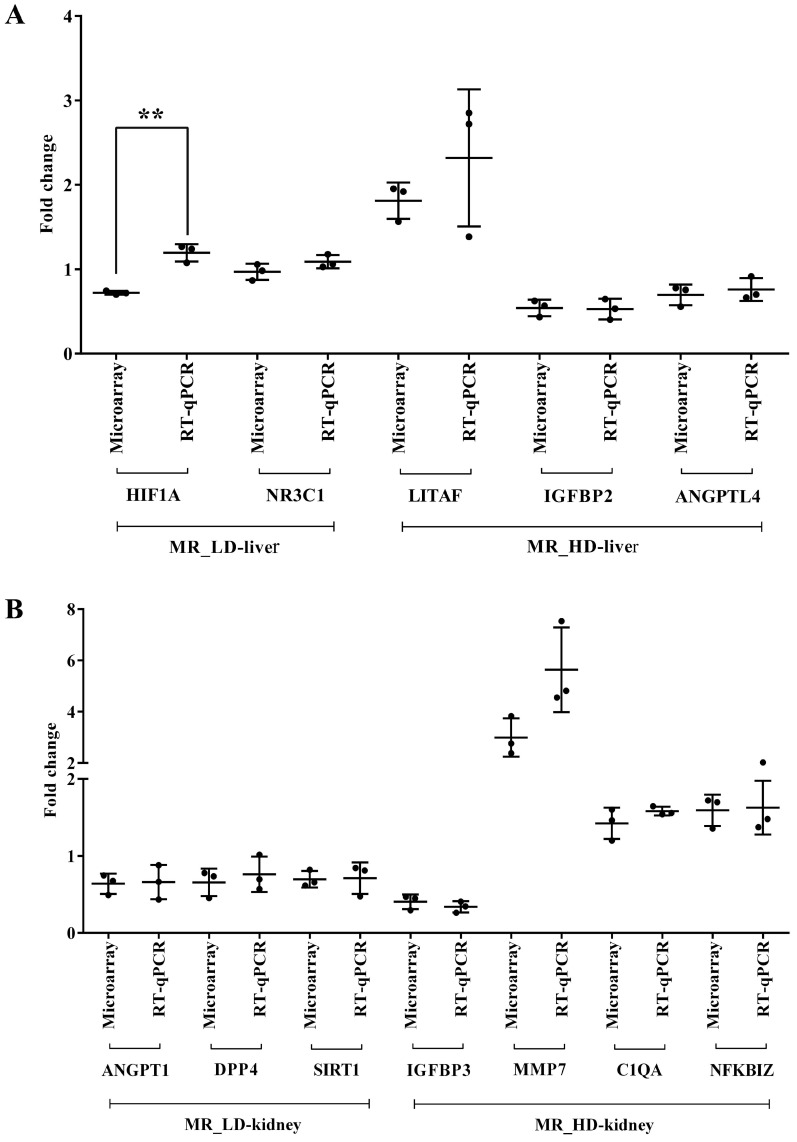
Experimental validation of master regulators by quantitative real-time PCR. **Panel A**: Expression of hepatic master regulator genes after low- and high-dose diclofenac treatment. **Panel B**: Expression of master regulatory genes in kidney after low- and high-dose diclofenac treatment. The y-axis indicates the individual fold changes in treated animals (diclofenac-treated vs. controls). Data are Fold change ± SD. ** *p* < 0.01. MR = master regulator, LD = low-dose, HD = high-dose.

**Figure 20:**
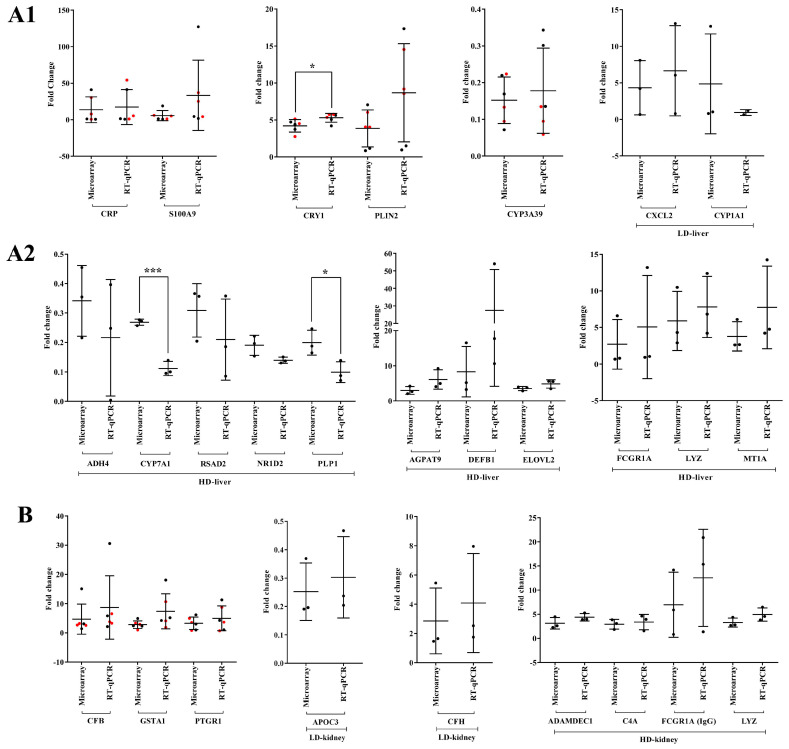
Experimental validation of highly regulated genes in liver and kidney in response to diclofenac treatment. **Panel A1,A2:** Liver-regulated genes in response to low- and high-dose diclofenac treatments. **Panel B:** Highly regulated genes in the kidney. The y-axis indicates the individual fold changes in treated animals (diclofenac-treated vs. controls). Fold changes obtained from high-dose treatments are marked in red color. Data are Fold change ± SD. * *p* < 0.05, *** *p* < 0.001. LD = low-dose, HD = high-dose.

We performed RT-qPCR assays to confirm the microarray data by an independent method and selected qPCR primers, which are identical to the probe sequences immobilized on the microarray. As shown in Figure 19 and Figure 20 we obtained similar results for the two platforms. Notwithstanding, the qPCR assays revealed the hepatic master regulators HIF1A (*p* < 0.05) and LITAF to be consistently higher in expression in low- and high-dose-treated animals (Figure 19A). In the kidney, the qPCR assays defined MMP7 transcripts as more abundantly expressed when compared to the microarray data (Figure 19B). Additionally, we evaluated highly regulated genes in the liver and kidney (Figure 20). Here, qPCR assays tended to suggest more abundant expressions of AGPAT9, DEFB1, FCGR1A, MT1A, PLIN2 and S100A9; however, and with the exception of CRY1 (*p* < 0.05), the data were statistically insignificant, mostly due to one outlier. In the cases of ADH4, CYP1A1, CYP7A1 (*p* < 0.001), NR1D2, PLP1 (*p* < 0.05) and RSAD2, the qPCR assay implied lesser expression (Figure 20A). Finally, we noticed a statistically insignificant increased expression of GSTA1 with kidney RNA extracts (Figure 20B).

## 3. Discussion

Diclofenac is commonly prescribed for the treatment of rheumatoid arthritis; however, its use is associated with a range of ADRs [6]. Its ability to induce idiosyncratic liver injury has been assessed for causality by RUCAM, and it ranks among the top five DILI-causing drugs [63]. The precise mechanisms underlying diclofenac’s potential to cause liver and kidney injury remain uncertain and multifactorial. Clinical and experimental evidence supports the notion of an immune/inflammatory-mediated mechanism of injury, which is surprising given its mode of action as a non-steroidal anti-inflammatory drug [3,8,19,20,21,64].

Here, we report a novel molecular circuitry whereby diclofenac desynchronizes the liver clock in its control of cellular metabolism and immune response and ultimately leads to liver injury. 

### 3.1. Diclofenac Reactive Metabolites

There is strong evidence for CYP monooxygenases and myeloperoxidases in neutrophils and Kupffer cells to catalyze the production of diclofenac-reactive metabolites. Therefore, different routes in the production of reactive metabolites exist. We observed significant repression (up to six-fold) of CYP3A22, CYP3A29, CYP3A39 and CYP3A46; however, induced neutrophil count (supplementary Figure S3D) and activated Kupffer cells (Figure 1, Figure 5 and Figure 6) highlighted the importance of myeloperoxidases (MPO) in the metabolism of diclofenac. Although the identified CYP monooxygenases are orthologues of human CYP3A4 [65,66,67], there are also differences in the regulation of porcine and human CYP3A4, as recently reported for CYP3A22 [68]. The reduced expression of porcine CYP monooxygenases is likely caused by hepatic inflammation in response to diclofenac treatment [69,70] and, given the highly significant increase in activated neutrophils (see above and Figure 6), we propose reactive metabolism to be primarily catalyzed by myeloperoxidase of neutrophils. Indeed, histopathology confirmed activated neutrophils and Kupffer cells in regions of harmed parenchyma, as shown by the MPO and CAE stains (Figure 5 and Figure 6), and we consider diclofenac-reactive metabolites as one of the causative events for liver injury [71].

Additionally, CYP7A1, i.e., the rate-limiting enzyme of bile acid synthesis, and the bile salt transporters ABCA8 and SLCO1B3 were up to four-fold repressed; their reduced expression correlated with elevated levels of hepatic triglycerides [72,73,74]. Importantly, the solute carrier SLCO1B3 transports glycoursodeoxycholate, and this bile acid is used as a therapeutic agent to treat cholestatic liver disease [75]. Moreover, glycoursodeoxycholate infusions prevented cholestatic liver disease induced by taurocholate in rats [76].

Probably, hepatic inflammation will also repress expression of phase II drug metabolizing enzymes, i.e., GSTA2, GSTT1, MGST3 and UGT1A6. However, induced expression of renal GSTA1 (>3 fold) and GSTP1 signifies the induction of defense programs against toxic electrophiles and oxidative stress, and their elevated activities have been reported to be a marker for renal proximal tubular necrosis [77]. Collectively, the results suggest reactive metabolites of diclofenac to play a critical role in hepato- and renal toxicity.

### 3.2. Diclofenac-Induced Expression of Marker Genes of Inflammation

Hepatic expression of ß-defensin (DEFB1), the chemokines CXCL2, CXCL13 and lysozyme (LYZ) were eight-, three-, two- and six-fold induced, whereas CCL8 and CXCL16 was repressed by three- and two-fold, respectively, after diclofenac treatment. Importantly, ß-defensins play a major role in preventing neutrophil apoptosis and function as pro-inflammatory mediators of the immune response [78]. The strong induction of DEFB1 and of lysozyme is part of a complex inflammasome [79] to hepatic injury and sterile inflammation. Moreover, a recent study reported DEFB1 to be induced during cholestasis, with bilirubin and bile acids modulating its expression [80]. In the present study, DEFB1 was > eight-fold induced; however, serum markers of cholestasis were either insignificantly increased or even repressed, and the observed inhibition of CYP7A has major implications on bile acid synthesis and bile pool size. Lastly, histopathology evidenced portal inflammatory infiltrates and extravasation of neutrophils to contribute to lobular inflammation [81].

For its decisive role in attracting polymorphonuclear leukocytes (PMLs) to sites of injury, the four-fold induced expression of macrophage inflammatory protein-2 (MIP-2)/CXCL2 is of significance [19,82,83]. Activated PMLs release diverse cytotoxic factors to trigger oxidative stress and cellular damage [84,85,86], and in the present study, blood smears and histopathology showed neutrophils to be the prominent cell type in diclofenac-induced liver injury (see above). 

There have been case reports of diclofenac-induced immune hemolysis [87] and delayed hypersensitivity reactions [88]. One study investigated antibody responses to diclofenac and some of its metabolites in a cohort of 59 patients who experienced hypersensitivity reactions [89]. There was little evidence for an IgE-mediated response. Therefore, the authors excluded an involvement of prominent metabolites in diclofenac hypersensitivity reactions. 

The role of chemokines in liver disease was the subject of a seminal review [90], and in the present study, CXCL13 was minimally (1.5-fold, *p* < 0.05) induced to possibly stimulate the homing and motility of B cells to sites of injury [91,92]. Moreover, we observed significant expression of the chemokine CXCL16 to about 60% of the controls, and this chemokine plays a role in the recruitment of natural killer (NK) T cells and is crucial in the initiation and progression of hepatic inflammation and fibrosis [93]. We consider its repression an adaptive response to lessen the damage induced by hepatic inflammation. Indeed, it has been shown that CXCL16 deficiency attenuates acetaminophen-induced hepatotoxicity by decreasing hepatic oxidative stress and inflammation in mice [94].

Furthermore, we report CCL8/monocyte chemotactic protein (MCP-2) as repressed to 40% of the controls; this chemokine is capable of binding to CCR2, a receptor that functions in the trafficking of immune cells to sites of inflammation [95]. There are additional reports implicating MCP-2 in liver inflammation [96,97,98]. We therefore consider its down-regulation as an adaptive response to alleviate the harmful effects of inflammation. Interestingly, CCL8 is a biomarker candidate for the diagnosis of graft-versus-host disease [99].

Moreover, the interleukin receptor beta subunit IL10RB was two-fold up-regulated in the liver and kidney, though the alpha subunit was unchanged. Both subunits form a heterotetrameric receptor complex and, upon activation by its ligand IL10, inhibit pro-inflammatory responses [100]. However, IL10 was unchanged, as were most IL10-responsive genes; nonetheless, we observed a minor but statistically significant up-regulation of IL1B, JAK3, MYD88, NFKB1A and NFKB1B (~ 1.5-fold, *p* < 0.05). Together, this led to the notion that the IL10/IL10R signaling pathway was basically inactive.

Additionally, CXCL5 was two-fold up-regulated in the kidney, and this chemokine stimulates Th17-cell-mediated neutrophil migration to injured renal cells [101], whereas the nearly two-fold induced expression of CXCL14 regulates the chemotaxis of natural killer (NK) cells to sites of inflammation [102,103]. Similarly, CCL24/eotaxin-2 was mildly up-regulated to support the trafficking of eosinophils to sites of injury [104,105]. Conversely, the two-fold induced expression of intracellular cytokine receptor TNFRSF17/ B-cell maturation antigen (BMCA) might be regarded as an adaptive response to alleviate excessive neutrophil activity [106]. Furthermore, the two-fold repressed expression of TNFAIP6/TNF-stimulated gene 6 (*TSG-6*) suggests the inhibition of the negative feedback control in the inflammatory response [107].

The activation of complement factors emphasizes hypersensitivity reactions in response to diclofenac treatment. Specifically, we observed activation of components of the classical pathway, with about a two-fold induced expression of C1QA, C1QC, C1S, C1R and of co-factors of the alternative complement pathway, notably CFB and CFI, that were nearly three-fold up-regulated in the kidney after diclofenac treatment. Testimony to an activated classical and alternative pathway is the two-fold induced expression of C3 convertase, a key molecule that cleaves the C3 molecule [108]. The increased expression of anaphylatoxins activates the terminal complement components (C6, C7, C8 and C9) and forms a membrane attack complex (MAC) to cause cell lysis and tubulointerstitial injury in renal cells [109,110]. Notably, the terminal complement components C7 and C9 were two-fold induced in the liver after low-dose diclofenac treatment, and the activation of the classical and alternative complement systems evidences hypersensitivity reactions in response to diclofenac treatment.

### 3.3. Diclofenac Regulates Components of the Circadian Clock 

The present study provided strong evidence for diclofenac to induce alterations in core molecular clock components to directly influence the expression of genes coding for immune, inflammation and metabolic processes [46,111,112,113]. The core clock apparatus consisting of ARNTL/BMAL1, DEC2, NPAS2, NR1D2/ REV-ERB beta, RORC, CRY1, PER2 and DBP were regulated in the liver and kidney (supplementary Table S2). Therefore, diclofenac treatment desynchronized the circadian clock. Specifically, the activator proteins BMAL1 and NPAS2 form a heterodimer to elicit transcriptional responses at Ebox response elements of targeted promoters. A transcriptional feedback loop exists, whereby PER and CRY block its own heterodimerization upon critical expression levels. Furthermore, the nuclear receptors ROR and REV-ERBα/β control BMAL1 expression [46,114,115]. Diclofenac treatment caused a > four-fold induced CRY1 expression to underscore an activated core loop; however, PER2mRNA was dose-dependently repressed by > 60% to suppress the circadian negative feedback loop. Independent research has reported diurnal variation in *Per2* expression with low *Per2* expression protecting mice from liver injury in response to high-dose acetaminophen treatment [116]; however, low *Per2* exacerbated cholestatic liver damage as well as fibrosis [117]. Conversely, Chen et al. reported the hepatoprotective role of *Per2* in mice when treated with CCl4 [118]. Apparently, PER2 suppresses the mitochondrial uncoupling protein UCP2; however, in the present study, UCP2 was unchanged.

Meanwhile, the significant five-fold repression of nuclear receptor NR1D2/Rev-Erbß has major implications for metabolic homeostasis; its depletion represses expression of genes coding for metabolic enzymes and causes marked hepatic steatosis in mice [119]. Moreover, the accessory loop of the master clock regulates BMAL1 expression; its transcriptional up-regulation is dependent on RAR-related orphan receptor activity, whereas binding of NR1D2/Rev-Erbß to the BMAL1 promoter inhibits its transcription. Hepatic BMAL1 and RORC were 3- and 2-fold induced, and the > 80% repressed Rev-Erbß provided strong evidence for the accessory loop to be active. Independent of its role within the molecular clock, RORC plays a key role in lipid/glucose homeostasis, insulin resistance and inflammation [120].

Moreover, a recent study demonstrates BMAL1 to regulate circadian expression of drug-metabolizing enzymes in mice [121]. Essentially, BMAL1 binds to the Hnf4α promoter to stimulate its transcription, whereas the regulation of the CYP monooxygenase 3a11 by *Bmal1* is Dbp- and Hnf4α dependent. *Bmal1* deficiency sensitizes mice to drug toxicities [121]. Although diclofenac treatment of minipigs did not influence HNF4α expression itself, it repressed DBP gene expression dose-dependently by 60%. The role of DBP in the circadian clock has been the subject of independent research [122,123,124,125], and its down-regulation represses the expression of drug-metabolizing enzymes as observed in the present study [126]. 

The importance of the circadian rhythm in the pathogenesis and treatment of fatty liver disease and alcohol-induced liver injury has been the subject of recent reviews [127,128]. Furthermore, the inhibition of Period1 represses CYP2E1-reactive metabolite production in mice following CCL4 treatment [129]. Thus, members of the liver clock may be explored as therapeutic targets to prevent reactive metabolite-induced liver injury. 

Collectively, these results suggest that diclofenac desynchronizes the molecular clock in the liver and kidney with major implications for drug detoxification.

### 3.4. Diclofenac Induces Cell Cycle Arrest and Apoptosis

The response gene to complement 32 (RGC-32) is a cell cycle regulator, and we found its expression two-fold repressed in the liver. Its down-regulation influences cell proliferation and apoptosis [130,131] and impairs macrophage activity [132]; however, it protects the liver from hepatic steatosis by decreasing the expression of lipogenic genes [133]. RGC32 is also a negative regulator of T-lymphocytes; its complete ablation causes CD4+ and CD8+ T cell proliferation [134]. Collectively, we consider its repression as part of a complex network to influence immune responses, monocyte-macrophage differentiation and hepatic metabolism. Furthermore, diclofenac treatment repressed caveolin 1 (CAV1) expression by 50%, and this scaffolding protein is a major regulator of cell signaling, inflammation and liver function [135,136]. Dysfunctional CAV1 leads to hepatic steatosis [137] and an altered mitochondrial metabolism and is associated with cholesterol-mediated mitochondrial dysfunction. CAV1 stimulates apoptosis by increasing cholesterol accumulation in mitochondrial membranes, thereby decreasing the efficiency of the respiratory chain and the intrinsic antioxidant defense [138]. Moreover, the nearly three-fold induced expression of cyclin-dependent kinase (CDK) inhibitor p21 facilitates p53-mediated cell cycle arrest and cell death in response to oxidative stress [139,140]. Furthermore, the nearly 70% repression of cyclin-dependent kinase inhibitor p27 (CDKN1B1) stimulates CDK2 activity in the absence of growth factors and disables the protection of cells from apoptosis [141]. Additionally, the expression of alcohol dehydrogenase 4 (ADH4) was significantly reduced to 30% of controls, and an induced expression of ADH4 has been implicated in ROS-mediated DNA damage in both alcoholic and non-alcoholic steatohepatitis [142,143], while its deficiency impairs the metabolism of retinol to retinoic acid. Likewise, the transcription factor CCAAT/enhancer-binding protein beta (C/EBPβ) was significantly up-regulated in the kidney and has been shown to be associated with ER-stress-mediated cell death via an unfolded protein response [144]. Furthermore, diclofenac caused a three-fold induced expression of S100A8/A9 in the kidney, and this acute phase reactant induces apoptosis and tubular injury in vivo by accumulating macrophages and/or activated neutrophils at sites of inflammation [145,146]. Diclofenac treatment also caused a two-fold induced expression of L-selectin (SELL), and this cell adhesion molecule regulates the trafficking of leukocytes on endothelial cells [147].

In addition, diclofenac treatment stimulated defense response genes in the liver and kidney. Specifically, the 60% repression of the liver-derived plasma protein, histidine-rich glycoprotein (HRG) dampens macrophage activation and inflammation by blocking the differentiation of inflammatory M1 macrophages [148]. Conversely, the two-fold induced expression of the small heat shock protein (sHSP) family member, i.e., αB-crystallin (CRYAB), HSP27 and the three-fold induced expression of NAD(P)H: quinone oxidoreductase 1 (NQO1) in the kidney might be regarded as a cytoprotective response against oxidative stress and other apoptotic stimuli [149,150].

### 3.5. Diclofenac-Induced Hepatic Steatosis

Consistent with our previous studies in mice and dogs, we observed significant up-regulation of ELOVL fatty acid elongase 2 (ELOVL2), perilipin 2 (PLIN2) and very low-density lipoprotein receptor (VLDLR) in the liver of diclofenac-treated minipigs (Table 6). The up-regulation of these genes is associated with hepatic steatosis through increased intracellular triacylglycerides synthesis and lipid droplet accumulation [151,152,153,154]. Similarly, we found the peroxisome proliferator-activated receptor gamma coactivator 1-alpha (PPARGC1/ PGC-1α), i.e., a transcriptional co-activator of PPARG repressed by 50% in the liver. Earlier studies have shown that gene silencing of PGC-1α reduced hepatic steatosis but augmented IL10 expression. Although IL10 itself was unchanged, diclofenac treatment caused a nearly three-fold induction of the interleukin-10 receptor subunit beta. Conversely, one study reported PGC-1α protein levels to be repressed in fatty livers of mice to dampen mitochondrial biogenesis, and PGC-1 ko mice were reported to develop multi-system energy metabolic derangements, muscle dysfunction and hepatic steatosis [155,156]. Together, PGC-1α is a key component of the liver clock in the control of energy metabolism and reinforces the notion that diclofenac desynchronizes the circadian clock and associated metabolic pathways [157]. This transcriptional coactivator also influences the expression of DBP and CCL8, which were significantly repressed by >60%. In this regard, the >50% repressed acetoacetyl-coenzyme A synthetase (AACS) in the liver of diclofenac-treated animals is of considerable importance. This cytosolic enzyme utilizes the ketone acetoacetate that originates from mitochondrial metabolism, e.g., ß-oxidation of fatty acids to produce acetoacetyl-CoA and is a substrate for the rate-limiting enzyme in cholesterol biosynthesis, i.e., HMG-CoA synthease 1 [158]. While HMG-CoA synthase and HMG-CoA reductase were insignificantly induced (up to 2.5-fold), the gene coding for mevalonate kinase was repressed to 40% of the controls (p<0.01). The gene expression changes agreed with the lower serum cholesterol levels determined for diclofenac-treated animals; however, the results did not reach statistical significance. Furthermore, ketone bodies are a source of energy, and the treatment-related statically significant hypoglycemia may be part of this process, i.e., a treatment-related metabolic switch towards lipogenesis. Collectively, a disturbed liver clock will have major implications for glucose homeostasis.

### 3.6. Transcription Factor—And Master Regulatory Gene Networks

The organ-specific genomic responses enabled us to perform computational analysis on gene-specific promoters. This revealed MEF2 and ARID5A as significantly over-represented in promoters of liver-regulated genes, even though their transcription factors were unchanged after low-dose diclofenac treatment. Notably, 67% of DEGs had enriched TFBS for these two TFs, and the codes for immune, inflammation, stress and circadian clock genes, of which CRY1, malic enzyme 3 and DPP4 are prominent examples.

We performed a computational genomic foot print analysis, and this revealed KLF- and GRE-enriched binding sites in promoters of regulated genes. Diclofenac treatment repressed KLF11 expression by 50% and knock down of KLF11 in mice increased hepatic triglyceride levels. Consistent with these findings, we observed treatment-related hepatic steatosis (supplementary Figure S1C) and this TF regulates hepatic lipid metabolism [159]. Another study linked KLF11 to hepatic glucose metabolism and regulation of PGC-1α [160]. As discussed above, we observed repressed PGC-1α expression and blood glucose levels were consistently lower in high-dose diclofenac-treated animals. Together, the data strongly suggest a regulatory circuitry of hepatic lipid and glucose metabolism that involves KLF11.

Additionally, the gene coding for the glucocorticoid receptor NR3C1 was marginally but significantly induced after low-dose diclofenac treatment. However, immunohistochemistry evidenced significantly induced hepatic NR3C1 activity (Figure 17E,F). As shown in Table 5, 64 DEGs are target genes of NR3C1, and 59 are regulated after high-dose diclofenac treatment. Furthermore, diclofenac treatment caused adrenocortical hypertrophy and increased glucocorticoid production. The thymic atrophy results from cytolysis and is typical for experimental animals at elevated glucocorticoid/cortisol blood levels [161]. We regard repression of the cortisol-binding protein by 40% as adaptive responses to elevated glucocorticoid levels in the systemic circulation. Together, the liver clock and NR3C1 drive master regulators and liver metabolic function. Given that REV-ERB connects the circadian clock with hepatic GR action, its >80% repression alleviates stress responses, as shown by the highly significant repression of the chemoattractants CXCL9 (90%), CCL8 (60%) and RSAD2 (70%).

For the liver, the gene regulatory network analysis revealed the following key regulators, i.e., CRY1, HIF1A, NR3C1, IGFBP2, LITAF and ANGPTL4 after low- and high-dose diclofenac treatment. Importantly, CRY1 is a key transcriptional repressor of the NPAS2-BMAL1 components of the circadian clock and was significantly up-regulated (four-fold) in response to diclofenac treatment. Overexpression of CRY1 attenuates hepatic gluconeogenesis by inhibiting the glucagon and glucocorticoid receptor signaling and decreases blood glucose levels by increasing insulin sensitivity [162,163]. Consistent with this notion, we showed diclofenac-treated minipigs to steadily present lower blood glucose levels (supplementary Figure S2). Furthermore, HIF1A is a key transcription factor in regulating cellular responses to hypoxia and the activation of innate and adaptive immune responses during tissue inflammation [164,165]. In the present study, HIF1A was significantly repressed at the transcript level (Table 8), but not at the protein level (Figure 5). Seemingly, HIF1A ko mice are protected from lipid accumulation and inflammation in response to LPS- and alcohol-induced liver damage [166,167]. 

We have already highlighted the importance of the glucocorticoid receptor (NR3C1) in immune and inflammatory responses and genes regulated by NR3C1, as well as the pro-inflammatory transcription factors, i.e., NFkB and STAT (see supplementary Table S4 and [168,169,170]. These cis-regulatory factors were significantly enriched in DEGs after repeated diclofenac treatment, and the results confirm our earlier findings with mice and dogs. Therefore, our study highlights the high conservation of regulatory gene networks across different species [19,20]. Moreover, we observed the up-regulation of the LPS-induced pro-inflammatory cytokine and fibrogenic activator LITAF in the liver. The protein is highly expressed in monocytes/macrophages, but also in tissue, such as liver, and binds to the CTCCC responsive element within the *TNF*-α promoter [171,172]. Lipopolysacharide-induced TNF factor (LITAF) connects inflammation to fatty liver disease, and its expression correlates with histological grades of fibrosis [172].

Additionally, diclofenac treatment caused repressed hepatic expression of insulin-like growth factor (IGF) modulator protein IGFBP2 and the PPARγ target gene ANGPTL4. The repression of these genes and their association with hepatic glucose homeostasis, insulin sensitivity and increased serum triglycerides and hepatic steatosis have been the subjects of independent reports [173,174,175,176,177]. Although PPARγ itself was unchanged the peroxisome proliferator-activated receptor-γ coactivator, PGC-1α was significantly repressed, and this protein interacts with many transcription factors and is a key regulator of energy metabolism [178].

## 4. Materials and Methods

### 4.1. Animals

We performed the animal study (dosing of animals, clinical chemistry) at PWG Genetics Pte Ltd. in Singapore and the genomic study at the Korea Institute of Toxicology (KIT) in Daejeon, Republic of Korea. All other work (histopathology, clinical and genomic data analysis and bioinformatics, immunohistochemistry, data interpretation, etc.) was performed at Hannover Medical School, Germany. The CRO in Singapore was accredited by the “Association for Assessment and Accreditation of Laboratory Animal Care”, and the research was performed according to Good Laboratory Practice principles. The study complied with the principles of the OECD guideline for a repeated dose toxicity study in non-rodent species. Ethical approval was obtained according to Singapore and Korean law. In addition, approval to conduct animal studies was obtained from the Institutional Animal Care and Use Committee (IACUC) of the Korea Institute of Toxicology (KIT) in Daejeon, Republic of Korea.

A total of nine specific pathogen free (SPF) male miniature pigs (Sus scrofa) were acclimatized to the animal husbandry prior to drug treatment and were kept in a controlled environment, i.e., temperature (20–30 °C), a humidity of 50–80%, an air circulation of 15 times/hour and a 12-h light/dark cycle at 150–300 lux. We housed the animals in groups of three per pen, and the minipigs received certified food pellets of 300 g/day (T.S Corporation, Incheon, Korea). Water was given ad libitum.

### 4.2. Drug Treatment

Prior to dosing, animals did not have access to food. However, one hour after dosing the animals received 300 g of food pellets, which remained in the feeding trough until the end of the day.

We purchased sodium salt of diclofenac from Sigma-Aldrich, Korea (CAS No: 15307-79-6) and loaded the drug into hard gelatin capsules, size #12 (Torpac Inc., Fairfield, NJ, USA). Controls (N = 3) received one empty capsule per day (= vehicle control), and treatment animals (N = 3/dose) were dosed with either 3 mg/kg (low-dose) or 15 mg/kg diclofenac (high-dose) for 28 days. 

The doses of 3 and 15 mg/kg/day diclofenac were selected based on the results obtained in the 2-week dose-range-finding (DRF) study. A dose of 28 mg/kg/day diclofenac was established as the maximum tolerated dose (MTD), and the low-dose of 3 mg/kg was identical to the high-dose in the dog study [20]. Importantly, the maximum clinical dose (MCD) was 40 mg/kg (2400 mg diclofenac/60 kg body weight); therefore, both doses were clinically relevant.

We recorded body weight and food consumption during the entire study period, and we euthanized the animals by exsanguination under deep barbiturate anesthesia (Thiopental sodium).

### 4.3. Clinical Pathology

The animals were fasted overnight prior to blood collection, and samples were collected via jugular vein puncture at pre-treatment day 1 (prior to start of dosing) and days 8, 14 and 28 for an evaluation of hematology and serum biochemistry readouts. For hematology, the blood samples were collected in EDTA-2K-containing tubes, and the parameters white blood cells (WBC), red blood cells (RBC), hemoglobin (HGB), hematocrit (HCT), mean corpuscular volume (MCV), mean corpuscular hemoglobin (MCH), mean corpuscular hemoglobin concentration (MCHC), platelet (PLT), reticulocyte (RET) and differential leukocyte count were measured on an ADVIA 2120 hematology system (Siemens, Germany). In addition, we obtained blood serum samples using standard operating procedures and determined aspartate aminotransferase (AST), alanine aminotransferase (ALT), alkaline phosphatase (ALP), gamma glutamyl transferase (GGT), total bilirubin (TBIL), glucose (GLU), albumin (ALB), total protein (TP), triglycerides (TG) and cholesterol (CHOL) blood urea nitrogen (BUN) and creatinine (CREA) on a 200FR NEO system (Toshiba Co., Tokyo, Japan). Furthermore, we analyzed the serum electrolytes (Na, K, Ca and Cl) on a 200FR NEO (Toshiba Co.).

### 4.4. RNA Extraction

The liver and kidneys from control and diclofenac-treated animals were surgically removed, and tissue samples were snap-frozen in liquid nitrogen. Prior to RNA extraction, we homogenized the tissues in a TissueLyser (Qiagen, Hilden, Germany) according to the manufacturer’s recommendations. Briefly, we placed the tissues in 2 mL tubes, which contained stainless steel beads and a lysis buffer, and operated the system at 20–30 Hz for 2 min. Subsequently, we isolated total RNA with the RNase mini kit (Qiagen) according to the manufacturer’s recommendations and as previously reported [19,20]. We measured the concentration of total RNA in a NanoDrop spectrophotometer (NanoDrop Technologies, Wilmington, DE, USA) and determined RNA integrity with the 2100 Bioanalyzer (Agilent Technologies, Santa Clara, CA, USA).

### 4.5. Microarray Experiments and Data Analysis

We performed whole genome expression profiling with the Affymetrix porcine GeneChip microarray system according to the manufacturer’s protocols (Affymetrix, Santa Clara, CA, USA). All steps of the cDNA synthesis, the biotin labeling, fragmentation of cRNA, hybridization, staining, washing and scanning on a GeneChip Scanner 3000, were carried out as previously reported [19,20,179].

We performed data normalization with the MAS5 algorithm by comparing the gene expression data from low-(3 mg/kg/day) and high-(15 mg/kg/day) dose-treated animals against controls. Subsequently, we defined differentially expressed genes (DEGs) in normalized datasets by performing hypergeometric tests with the cut-off criteria fold change > 1.5 and *p*-value < 0.05. In order to control the false discovery rate (FDR) at α 0.05, we only considered p-values corrected by the *Benjamini–Hochberg* procedure. We grouped genes satisfying these conditions as up- and down-regulated and constructed heat maps to identify commonly regulated DEGs by applying the average-linkage hierarchical clustering algorithm with Euclidean distance (Multi Experimental Viewer, http://www.tm4.org/mev.html, accessed on 4 January 2023).

### 4.6. RT-qPCR Validation of Microarray Data

We validated the differentially expressed genes by quantitative real-time RT-PCR. We purchased the primers from GenoTech (Daejeon, Korea). Total RNA (2 µg) was reverse-transcribed with SuperScript II (Invitrogen, Carlsbad, CA, USA) using an oligo-dT primer as suggested by the manufacturer. cDNA samples were stored at −20 °C until use. We performed RT-qPCR in a 20 µL reaction volume containing 0.5 µL (10pM) forward and reverse primers, 10 µL of SYBR Green master mix (Applied Biosystems, Waltham, MA, USA), 2 µL of cDNA and 7 µL of nuclease-free water. We amplified the cDNA on a StepOne and StepOnePlus Real-Time PCR System (Applied Biosystems) following the manufacturer’s protocols. We used the 18S ribosomal RNA primers as an internal control, and the primer sequences of all the genes investigated are listed in supplementary Table S6.

### 4.7. Histopathology

We applied standard operating procedures to evaluate the liver morphology of control and diclofenac-treated minipigs. The stains included Hematoxylin and Eosin (H&E), Periodic acid-Schiff reaction (PAS), PAS diastase digestion, Elastica van Gieson and silver stain.

### 4.8. Immunohistochemistry

Livers from control and diclofenac-treated animals were fixed in 4% buffered paraformaldehyde and embedded in a paraffin block using the standard protocols of the laboratory. Then, 1–2 μm thick sections were deparaffinized and rehydrated through a descending alcohol series followed by a 4 min washing step in distilled H2O. Subsequently, we performed antigen retrieval in citrate buffer (pH 6) or Tris-EDTA buffer (pH 9.0) in a water bath at 98 °C for 30 min. We used the ZytoChem-Plus HRP Polymer-Kit of Zytomed Systems (Berlin, Germany) for immunohistochemistry, and the slides were rinsed with distilled H_2_O. After a 5-min incubation step in tris-buffered saline (washing buffer), endogenous peroxidase activity was blocked with 3% peroxidase blocking reagent (Merck, Darmstadt, Germany) for 5 min followed by a second washing step. Thereafter, we applied protein-block serum free reagent for 5 min (ZytoChem-Plus HRP Polymer-Kit, reagent 1) and incubated the sections with primary antibodies for 60 min. We purchased the antibodies from diverse vendors and diluted them with washing buffer, as given in Table 9: 

We incubated the bound primary or bridging antibody with labeled polymer HRP anti-rabbit or anti-mouse secondary antibody (ZytoChem-Plus HRP Polymer-Kit, reagent 2) for 20 min and added reagent 3 of the ZytoChem-Plus HRP Polymer-Kit to finally place the slides in a moist chamber at room temperature, allowing an incubation time of 30 min. After completion of the HRP reaction, we counterstained the sections with Hematoxylin for 5 min, washed the slides under running warm tap water for 10 min and dehydrated the sections in a cabinet at 60 °C for 20 min. The sections were coverslipped and examined under a light microscope (Nikon Ni-E microscope, Japan), and we captured images using Nikon NIS basic research microscopic imaging software version 4.3.

### 4.9. Bioinformatics

#### 4.9.1. Identification of Orthologous Genes

Although several studies have reported the decoding of pig genomes, its annotation is not complete. Therefore, we utilized the g:Orth tool of the g:profiler (http://biit.cs.ut.ee/gprofiler/gorth.cgi, accessed on 4 January 2023); this tool retrieves orthologous genes from diverse organisms (human, mouse, rat) based on their sequence similarity [180].

#### 4.9.2. Enriched Biological Processes and Gene Ontology Mapping

To discern the functions of DEGs, we queried the GeneXplain platform version 6.0 (Wolfenbüttel, Germany) and considered biological processes, cellular components, molecular functions, metabolic pathways and transcription factor binding sites in promoters of regulated genes. We only considered enriched ontology terms with a *p*-value < 0.05 and a FDR corrected significance level of α = 0.05. Enriched gene ontology terms/pathways and their networks were visualized with ClueGO version 2.2.3; this tool was available as a Cytoscape plug-in. ClueGO retrieves information from precompiled data annotated within GO, KEGG and BioCarta databases, and the significance of an enrichment score is calculated based on the hypergeometric distribution of DEGs [181].

#### 4.9.3. Gene/Protein Interaction Network Construction

We constructed interaction networks using STRING software version 10.5 (http://string-db.org, accessed on 4 January 2023). This web-based tool informs on the interaction of genes/proteins by inferring associations based on publicly available and experimentally validated datasets, including high-throughput experimental data, the mining of data from diverse databases, published literature findings and predictions based on genomic context analysis. Meanwhile, a confidence score for each predicted association and constructed network is calculated [182].

#### 4.9.4. Identification of Upstream Master Regulatory Molecules

We identified master regulatory molecules and constructed associated gene networks with the tool GeneWays available within the GeneXplain platform. GeneWays is an open access platform used to automatically extract, analyze, visualize and integrate molecular pathway data from published peer-reviewed articles [183]. We set a default cut-off score of 0.2, FDR at 0.05 and a Z-score of 1.0 with a maximum radius of four steps upstream of input data sets for defining statistically significant master regulators. 

#### 4.9.5. Promoter Sequence Analysis

We queried the Genomatix software suite (Munich, Germany) and the GeneXplain platform for transcription factor binding sites (TFBS) at gene-specific promoters. Specifically, we performed in-silico genomic foot printing of >2000 transcription factors. We focused on DEGs coding for immune and stress response, inflammation, hypoxia, cytokine stimulus, acute-phase, cell death and oxidation reduction processes. We extracted the promoter sequences of DEGs with the Gene2Promoter tool of the Genomatix software, as well as preset functions termed “analyze the regulatory regions” of the GeneXplain platform. We defined the transcriptional start site (TSS) by the TFIIB recognition element, the TATA-box at the 5′-end, an initiator region around the TSS and a downstream promoter element (DPE) at the 3′-end. Subsequently, we interrogated the cis-regulatory binding sites of genomic sequences with a length of −1000 to +100 base pairs relative to TSS by utilizing the MatInspector tool (Genomatix software suite) or the preset function “analyze the regulatory regions” of the GeneXplain platform. We retrieved TFBS from the TRANSFAC 2020.1 library; this repository consists of 7626 predefined positional weight matrices (PWM). In addition, we queried circadian transcriptional regulators and glucocorticoid receptors based on their cognate recognition elements (E-box motives and glucocorticoid receptor binding sites). The overrepresentation of transcription factor binding sites was computed by comparing individual TFBS in promoters of DEG (= Yes-set) with non-regulated genes (= No-set). Enriched TFBS at gene-specific promoter regions were defined based on statistical significance (*p* < 0.05) and the Z-score.

#### 4.9.6. Composite Modules

We used the FrameWorker tool that is available within the Genomatix suite and the F-MATCH algorithm of the GeneXplain platform to infer composite modules. These are defined by the co-occupancy of regulatory TFBS in distinct promoter sequences. Typically, promoter activation involves more than one protein, and composite modules represent the next level of functional characterization. The following parameters of the FrameWorker tool were applied: The quorum constraint, i.e., the minimum number of sequences within the common framework is 80%, the minimum number of TFBSs in a framework is ≥ 2, the variation of distance range is 25 bp, the minimum distance is 5bp and the maximum distance between TFBS is 300 bp. In the case of the F-MATCH algorithm, we applied default functions. In addition, we interrogated DEGs for the co-occupancy of circadian clock transcriptional regulators and glucocorticoid receptor binding sites. As described above, the approach represents a variant of the F-MATCH algorithm for TFBS pairs and quantifies overrepresentation of promoter sequences in the foreground set using the Fisher test [184,185,186]. We applied the default filtering criteria of the GeneXplain platform and determined statistically significant composite modules.

## 5. Conclusions

Our study led us to propose a regulatory loop whereby diclofenac passes the blood-brain barrier to elicit stress responses via the SCN and the hypothalamus- pituitary adrenal gland axis. This results in enhanced adrenocorticotropic hormone (ACTH) secretion, adrenal ACTH dependent glucocorticoid synthesis and elevated glucocorticoids in the systemic circulation. Adrenal hypertrophy signifies persistent HPA axis signaling, and increased glucocorticoids in the circulation result in thymus atrophy. Genomic and immunohistochemistry studies provided strong evidence for diclofenac to desynchronize the liver clock and, together with induced glucocorticoid receptor activity, evokes transcriptional responses in liver metabolism. Notwithstanding, the neutrophil-dependent oxidation of diclofenac to reactive metabolites will also contribute to liver injury and inflammation.

### Study Limitations

We wish to highlight the following limitations to our study. First, given diclofenac’s ability to cause inflammation, we performed the study in males only. Specifically, female pigs are overrepresented for inflammatory cell infiltrates, as stated by the FDA’s Advisory Committee “Cellular, Tissue, and Gene Therapies” on pigs for toxicology studies [187,188]. Second, although extensive immunogenomic and histopathology investigations provided clear evidence for the treatment effects to be persistent, a time resolved assessment of the diclofenac treatment effects on the circadian rhythm would require many more animals. For animal welfare reasons, this was not permissive. Third, despite similarities in the anatomy and physiology of the liver and kidney of porcine and human origin, there are also important differences, especially in the immune system, which require consideration. Therefore, this preclinical model may not be entirely reflective of human diclofenac DILI and acute kidney injury cases. Moreover, because of the lack of suitable tissue biopsies, a direct comparison to human diclofenac DILI and kidney injury cases could not be performed. 

## Figures and Tables

**Table 1:** Commonly regulated hepatic DEGs after low- and high-dose diclofenac treatment. Minipigs were given 3mg/kg or 15 mg/kg daily for 28 days. Whole genome expression profiling was performed and DEGs were calculated based on the criteria fold change > 1.5 and a *p*-value < 0.05. Collectively, 70 genes were regulated in common when low- and high-dose treatment groups were compared.

Gene Symbol	Gene Description	Fold Change (Average) ± SD	
		LD_Liver	HD_Liver
**Immune and inflammatory response**			
ATG12	Autophagy related 12	1.48 ± 0.2	1.53 ± 0.15
BLNK	B-cell linker	−1.57 ± 0.08	−1.74 ± 0.48
CXCL2	C-X-C motif chemokine ligand 2	4.33 ± 1.26	2.56 ± 1.05
GBP1	Guanylate binding protein 1	−1.65 ± 0.65	−2.55 ± 0.54
HSP90AA1	Heat shock protein 90 alpha family class A member 1	1.58 ± 0.2	2.01 ± 0.3
HSPA5	Heat shock protein family A (Hsp70) member 5	1.54 ± 0.27	1.81 ± 0.65
IFIT1	Interferon induced protein with tetratricopeptide repeats 1	−1.75 ± 0.07	−2.03 ± 0.56
IL10RB	Interleukin 10 receptor, beta	1.9 ± 0.06	2.6 ± 0.28
IRG6	Inflammatory response protein 6	−1.82 ± 0.84	−3.24 ± 1.23
LYZ	Lysozyme	2.78 ± 1.93	5.89 ± 1.04
MAPK14	Mitogen-activated protein kinase 14	1.51 ± 0.27	1.53 ± 0.08
MAPK6	Mitogen-activated protein kinase 6	1.52 ± 0.30	2.17 ± 0.5
PRKD1	Protein kinase D1	−2.04 ± 1.13	−2.52 ± 0.42
SPP1	Secreted phosphoprotein 1	−1.53 ± 0.13	−1.58 ± 0.67
THY1	Thy-1 cell surface antigen	−1.68 ± 0.27	−1.54 ± 0.38
**Response to oxidative stress**			
NUDT15	Nudix hydrolase 15	1.53 ± 0.25	1.62 ± 0.11
SOD2	Superoxide dismutase 2, mitochondrial	2.22 ± 0.9	1.91 ± 0.53
**Response to stress**			
PLLP	Plasmolipin	1.62 ± 0.48	1.7 ± 0.41
PTPLAD1	Protein tyrosine phosphatase-like A domain containing 1	−1.78 ± 0.19	−1.87 ± 0.14
RBM3	RNA binding motif (RNP1, RRM) protein 3	−1.51 ± 0.15	−1.93 ± 0.12
SPR	Sepiapterin reductase	−1.61 ± 0.49	−1.67 ± 0.38
STC1	Stanniocalcin 1	1.54 ± 0.3	1.58 ± 0.35
**Cell death and apoptosis**			
DNPH1	2’-deoxynucleoside 5’-phosphate N-hydrolase 1	−1.52 ± 0.48	−1.57 ± 0.15
FYN	FYN Proto-Oncogene, Src Family Tyrosine Kinase	−1.52 ± 0.21	−1.56 ± 0.24
GAS1	Growth Arrest Specific 1	−1.53 ± 0.22	−1.52 ± 0.32
HSPH1	Heat shock protein family H (Hsp110) member 1	1.74 ± 0.4	1.77 ± 0.48
KLF11	Kruppel like factor 11	−1.77 ± 0.64	−1.88 ± 0.38
VIL1	Villin 1	−1.89 ± 0.35	−1.79 ± 0.58
**Circadian rhythm**			
ARNTL/BMAL1	Aryl hydrocarbon receptor nuclear translocator-like	2.07 ± 0.46	2.69 ± 0.1
CRY1	Cryptochrome 1	4.13 ± 1.23	4.28 ± 0.51
DBP	D-box binding PAR bZIP transcription factor	−1.83 ± 0.35	−2.41 ± 0.65
NR1D2/ REVERBB	Nuclear receptor subfamily 1, group D, member 2	−2.79 ± 0.38	−5.25 ± 1.01
**Drug metabolism**			
CYP3A39	Cytochrome P450 3A39	−6.52 ± 2.58	−6.78 ± 1.82
CYP3A46	Cytochrome P450 3A46	−1.95 ± 1.42	−1.9 ± 0.34
NR1I3 (CAR)	Nuclear Receptor Subfamily 1 Group I Member 3	1.42 ± 0.25	1.77 ± 0.1
**Cell cycle**			
CENPF	Centromere protein F	−1.59 ± 0.2	−1.51 ± 0.28
FOXA1	Forkhead box A1	1.6 ± 0.55	1.79 ± 0.37
TOP2A	Topoisomersae II	−1.62 ± 0.49	−1.77 ± 0.33
USP2	Ubiquitin specific peptidase 2	−2.08 ± 2.26	−1.91 ± 0.42
**Lipid metabolism**			
ACSL3	Acyl-CoA synthetase long-chain family member 3	−1.52 ± 0.83	−1.66 ± 0.58
DDHD1	DDHD domain containing 1	1.5 ± 0.38	1.73 ± 0.27
ECHDC3	Enoyl-CoA hydratase domain containing 3	−1.44 ± 0.31	−1.58 ± 0.42
ELOVL2	ELOVL fatty acid elongase 2	2.66 ± 0.9	3.53 ± 0.59
FASN	Fatty Acid Synthase	−1.61 ± 0.22	−2.12 ± 0.54
FNDC3B	Fibronectin type III domain containing 3B	1.42 ± 0.2	2.09 ± 0.33
KRT8	Keratin 8	1.9 ± 0.38	2.6 ± 0.28
KRT18	Keratin 18	1.74 ± 0.62	1.67 ± 0.25
LPPR4	Lipid phosphate phosphatase-related protein type 4	−2.04 ± 0.41	−2.38 ± 0.79
PLIN2	Perilipin 2	3.01 ± 1.39	4.7 ± 1.15
PLP1	Proteolipid protein 1	−2.4 ± 1.2	−5.01 ± 1.01
PPARGC1A	Peroxisome proliferator activated receptor gamma, coactivator 1 alpha	−1.63 ± 0.28	−2.11 ± 0.46
SERPINA6	Serpin peptidase inhibitor, clade A, member 6	−1.6 ± 0.57	−1.54 ± 0.33
SERPINE1	Serpin family E member 1	1.87 ± 0.95	1.58 ± 0.04
ST3GAL1	ST3 beta-galactoside alpha-2,3-sialyltransferase 1	1.53 ± 0.27	1.68 ± 0.27
VLDLR	Very low density lipoprotein receptor	−1.53 ± 0.21	1.98 ± 0.15
**Glucose metabolic process**			
ENO4	Enolase family member 4	−1.9 ± 1.02	−2.40 ± 0.40
**Collagen biosynthesis**			
COL5A3	Collagen type V alpha 3 chain	1.58 ± 0.11	1.74 ± 0.12
**Transmembrane transport**			
ATP6V1H	ATPase H+ transporting V1 subunit H	−1.51 ± 0.26	−1.44 ± 0.24
ATP9A	ATPase phospholipid transporting 9A	2.52 ± 0.24	1.6 ± 0.59
ODC	Ornithine decarboxylase	1.47 ± 0.24	2.13 ± 0.54
P2Y12R	Purinergic receptor P2Y12	−1.71 ± 0.61	−1.61 ± 0.35
SLC11A2	Solute carrier family 11, member 2	1.68 ± 0.33	1.97 ± 0.4
SLC1A1	Solute carrier family 1 member 1	1.53 ± 0.23	1.72 ± 0.31
SLC30A10	Solute carrier family 30, member 10	2.08 ± 0.68	2.21 ± 0.45
TMEM9	Transmembrane protein 9	−1.56 ± 0.12	−1.45 ± 0.35
**Signal transduction**			
ARHGAP1	Rho GTPase activating protein 1	1.62 ± 0.12	1.7 ± 0.17
CLDN11	Claudin 11	−1.72 ± 0.49	−1.94 ± 0.24
**Transcriptional regulation**			
RNPC3	RNA-binding region (RNP1, RRM) containing 3	1.57 ± 0.3	1.59 ± 0.35
ZNF12	Zinc finger protein 12	−1.62 ± 0.34	−1.87 ± 0.41
ZNF280D	Zinc finger protein 280D	1.61 ± 0.21	1.88 ± 0.4

**Table 2:** Commonly regulated genes in kidney after low- and high-dose diclofenac treatment. A total of 66 statistically significant DEGs were regulated in common amongst low- and high-dose diclofenac treatments.

Gene Symbol	Gene Description	Fold Change (Average) ± SD	
		LD_Kidney	HD_Kidney
**Immune and inflammatory response**			
C1QA	Complement C1q A chain	2.33 ± 1.62	1.51 ± 0.2
C1QC	Complement C1q C chain	2.31 ± 1.61	1.64 ± 0.25
CCL28	Chemokine (C-C motif) ligand 28	1.52 ± 0.21	1.58 ± 0.12
CFB	Complement Factor B	6.55 ± 2.45	2.82 ± 0.34
CFH	Complement Factor H	2.86 ± 2.25	1.91 ± 0.38
CXXC1	CXXC finger protein 1	−1.59 ± 0.17	−1.55 ± 0.06
HSP90B1	Heat shock protein 90kDa beta, member 1	−1.6 ± 0.4	−1.64 ± 0.12
IFITM2	Interferon induced transmembrane protein 2	1.66 ± 0.43	1.57 ± 0.15
IL10RB	Interleukin 10 receptor, beta	1.51 ± 0.28	1.93 ± 0.11
IRG6 (RSAD2)	Inflammatory response protein 6	−1.99 ± 0.17	−2.75 ± 0.59
KIF3A	Kinesin family member 3A	−1.56 ± 0.1	−1.63 ± 0.11
KNG1	Kininogen 1	−1.96 ± 1	−2.13 ± 0.14
PTGR1	Prostaglandin reductase 1	3.4 ± 2.61	3.13 ± 1.91
SAA2	Serum amyloid A2	2.12 ± 1.37	1.54 ± 0.13
USP18	Ubiquitin specific peptidase 18	−1.52 ± 0.08	−1.68 ± 0.47
VCAM1	Vascular cell adhesion molecule 1	1.87 ± 1.1	1.57 ± 0.63
**Response to stress**			
AK3L1	Adenylate kinase 3-like 1	−1.58 ± 0.15	−1.79 ± 0.1
CHD2	Chromodomain helicase DNA binding protein 2	−1.53 ± 0.22	−2.13 ± 0.28
COL3A1	Collagen, type III, alpha 1	1.57 ± 0.88	1.51 ± 0.17
GSTA1	Glutathione S-transferase alpha 1	3.23 ± 1.40	4.6 ± 1.27
LRP11	Low density lipoprotein receptor-related protein 11	1.55 ± 0.11	1.56 ± 0.32
RAD50	RAD50 homolog	−1.58 ± 0.03	−1.79 ± 0.33
TCEA1	Transcription elongation factor A (SII), 1	−1.61 ± 0.21	−1.71 ± 0.1
UBXN4	UBX domain protein 4	−1.52 ± 0.31	−1.92 ± 0.24
UPF3B	UPF3 regulator of nonsense transcripts Homolog B	−1.73 ± 0.3	−1.51 ± 0.15
ZCCHC11	Zinc finger, CCHC domain containing 11	−1.56 ± 0.19	−1.55 ± 0.09
**Cell death and apoptosis**			
CRYAB	Crystallin, alpha B	2.02 ± 0.43	2.1 ± 0.63
DAB2	Dab, mitogen-responsive Phosphoprotein, homolog 2	−1.63 ± 0.08	−1.62 ± 0.21
DSG2	Desmoglein 2	1.55 ± 0.3	2.12 ± 0.62
IGFBP3	Insulin-like growth factor binding protein 3	−1.83 ± 0.98	−2.47 ± 0.7
PDPK1	3-phosphoinositide dependent protein kinase 1	−1.55 ± 0.06	−1.74 ± 0.06
PPTC7	PTC7 protein phosphatase homolog	−1.49 ± 0.51	−2.31 ± 0.77
SCAF11	SR-related CTD-associated factor 11	−1.67 ± 0.36	−2.1 ± 0.49
UCP2	Uncoupling protein 2 (mitochondrial, proton carrier)	1.59 ± 0.22	1.78 ± 0.36
**Cell cycle process**			
SEPT7	Septin 7	−1.52 ± 0.21	−1.63 ± 0.21
CDC5L	Cell division cycle 5-like	−1.53 ± 0.12	−1.77 ± 0.18
PRIM2	Primase, DNA, polypeptide 2	−1.64 ± 0.22	−1.55 ± 0.12
**Circadian rhythmic process**			
CCND2	Cyclin D2	−1.56 ± 0.18	−1.7 ± 0.06
CRY1	Cryptochrome circadian clock 1	1.62 ± 0.32	1.82 ± 0.53
NR1D2/REVERBB	Nuclear receptor subfamily 1, group D, member 2	−1.99 ± 0.56	−1.99 ± 0.77
PER2	Period circadian clock 2	−1.56 ± 0.32	−1.85 ± 0.29
**Lipid metabolism**			
FADS6	Fatty acid desaturase domain family, member 6	1.56 ± 0.31	1.87 ± 0.29
KRT8	keratin 8	1.51 ± 0.28	1.93 ± 0.11
LDLR	Low density lipoprotein receptor	1.52 ± 0.2	1.51 ± 0.1
SIRT1	Sirtuin 1	−1.51 ± 0.21	−1.52 ± 0.38
**Protein metabolism**			
ARSJ	Arylsulfatase family, member J	1.53 ± 0.02	1.75 ± 0.29
RBM3	RNA binding motif (RNP1, RRM) protein 3	−1.52 ± 0.27	−2.56 ± 0.55
TET2	Tet methylcytosine dioxygenase 2	−1.67 ± 0.04	−1.83 ± 0.18
TTL	Tubulin tyrosine ligase	1.84 ± 0.19	1.89 ± 0.36
**Collagen biosynthesis**			
COL16A1	Collagen Type XVI alpha 1	1.55 ± 0.35	1.94 ± 0.12
COL21A1	Collagen, type XXI, alpha 1	−1.51 ± 0.37	−2.09 ± 0.41
**Signal transduction**			
ARHGAP29	Rho GTPase activating protein 29	−1.81 ± 0.17	−1.94 ± 0.28
GPNMB	Glycoprotein (transmembrane) nmb	2.48 ± 0.39	2.47 ± 0.85
S100A6	S100 Calcium Binding Protein A6	2.49 ± 1.83	1.74 ± 0.29
**Transmembrane and ion-channel transport**			
FXYD4	FXYD Domain Containing Ion Transport Regulator 4	1.61 ± 0.34	1.62 ± 0.12
GAPVD1	GTPase activating protein and VPS9 domains 1	−1.55 ± 0.05	−1.89 ± 0.3
SLC25A25	Solute carrier family 25 (mitochondrial; phosphate carrier), member 25	1.68 ± 0.27	2.19 ± 0.57
SLC4A4	Solute carrier family 4 (sodium bicarbonate cotransporter), member 4	−2.34 ± 0.16	−2.33 ± 0.42
TMEM87A	Transmembrane protein 87A	−1.54 ± 0.1	−1.53 ± 0.14
**Transcriptional regulation**			
DDX6	DEAD box helicase 6	−1.52 ± 0.21	−1.76 ± 0.12
GTF2IRD2	GTF2I repeat domain containing 2	−1.5 ± 0.41	−1.77 ± 0.29
HNRNPR	Heterogeneous nuclear ribonucleoprotein R	−1.55 ± 0.41	−1.82 ± 0.14
METTL12	Methyltransferase like 12	1.51 ± 0.21	1.61 ± 0.22
NOL8	Nucleolar protein 8	−1.54 ± 0.08	−1.65 ± 0.14
ZNF12	Zinc finger protein 12	−1.81 ± 0.25	−1.94 ± 0.21
**Vascular smooth muscle contraction**			
CALD1	Caldesmon 1	−1.94 ± 0.4	−2.55 ± 0.37

**Table 3:** Gene ontology enrichment of hepatic DEGs after diclofenac treatment. Gene ontologies were analyzed with the GeneXplain and ClueGO database; significantly enriched biological processes were considered at a *p*-value < 0.05. The percentage of genes associated with a given biological term and specific pathway were calculated with the AmiGO 2 database (http://amigo.geneontology.org/amigo/landing, accessed on 4 January 2023) and KEGG repository data entries.

GO ID.	Biological Process	Low-Dose			High-Dose		
		No of Genes (% Genes Associated with Terms)	*p*-Value	Adjusted *p*-Value	No of Genes (% Genes Associated with Terms)	*p*-Value	Adjusted *p*-Value
GO:0006955	Immune response	13 (0.59%)	0.0298	0.00150	42 (1.90%)	9.47 × 10^−4^	0.0153
GO:0006954	Inflammatory response	9 (1.17%)	0.0010	0.04570	13(1.69%)	0.0101	0.0428
GO:0006950	Response to stress	41 (1.06%)	9.30 × 10^−11^	0.02630	91 (2.36%)	4.58 × 10^−4^	0.0129
GO:0033554	Cellular response to stress	17 (0.90%)	1.16 × 10^−7^	0.00000	44 (2.34%)	0.0015	0.0241
GO:0001666	Response to hypoxia	9 (2.98%)	1.51 × 10^−4^	0.00000	14 (4.64%)	0.0094	0.0502
GO:0055114	Oxidation-reduction process	10 (0.91%)	0.0115	0.00110	37 (3.39%)	5.05 × 10^−4^	0.0136
GO:0006979	Response to oxidative stress	4 (1.14%)	1.12 × 10^−4^	0.02510	14 (3.28%)	6.07 × 10^−4^	0.0144
GO:0071345	Cellular response to cytokine stimulus	9 (0.88%)	0.0012	0.00901	25 (2.46%)	7.62 × 10^−5^	0.0033
GO:0019221	Cytokine-mediated signaling pathway	No enrichment			18 (2.50%)	0.0099	0.0496
GO:0060333	IFNG-mediated signaling pathway	No enrichment			7 (7.78%)	0.0325	0.0116
GO:0002694	Regulation of leukocyte activation	7 (1.25%)	0.0030	0.00500	19 (3.40%)	0.0123	0.0497
GO:0050900	Leukocyte migration	4 (0.84%)	0.0015	0.00970	10 (2.09%)	0.0339	0.1196
GO:0000165	MAPK cascade	5 (0.56%)	0.0013	0.00580	10 (1.12%)	0.0466	0.1400
GO:0007623	Circadian rhythm	6 (3.08%)	0.0028	0.04420	12 (6.15%)	0.0021	0.0292
GO:0007049	Cell cycle	6 (0.34%)	4.49 × 10^−4^	0.04190	22 (1.25%)	0.0330	0.1173
GO:0008219	Cell death and apoptosis	20 (0.92%)	0.0085	0.00250	49 (2.25%)	0.0054	0.0440
GO:0097190	Apoptotic signaling pathway	7 (1.20%)	7.45 × 10^−7^	0.02030	14 (2.41%)	0.0365	0.0143
GO:0006952	Defense response	14 (0.85%)	2.93 × 10^−6^	0.00001	46 (2.79%)	1.81 × 10^−4^	0.0053
GO:0001525	Angiogenesis	8 (1.69%)	0.0342	0.00010	14 (2.97%)	0.0058	0.0459
GO:0006805	Xenobiotic metabolic process	4 (3.22%)	0.0011	0.01740	9 (7.26%)	0.0157	0.0438
GO:0006629	Lipid metabolic process	No enrichment			44 (3.12%)	2.23 × 10^−4^	0.0083
GO:0006631	Fatty acid metabolic process	6 (1.66%)	0.0118	0.00460	14 (3.87%)	0.0284	0.1085
GO:0006006	Glucose metabolic process	4 (1.98%)	0.0083	0.02540	9 (4.46%)	2.58 × 10^−4^	0.0069
GO:0034762	Transmembrane transport regulation	12 (2.30%)	0.03964	0.04260	18 (3.45%)	0.0105	0.0717
hsa0415	PI3K-AKT signaling pathway	5 (1.41%)	0.0141	0.03076	11 (3.11%)	0.0056	0.0003
hsa03320	PPAR signaling pathway	No enrichment			7 (9.46%)	0.0099	0.0129

**Table 4:** Gene ontology enrichment of kidney-related DEGs after diclofenac treatment. Gene ontologies were analyzed with the GeneXplain and ClueGO databases; significantly enriched biological processes were considered at a *p*-value < 0.05. The percentage of genes associated with biological terms and pathways were calculated with the AmiGO 2 database (http://amigo.geneontology.org/amigo/landing, accessed on 4 January 2023) and KEGG repository data entries.

GO ID	Biological Process	Low-Dose			High-Dose		
		No of Genes (% Genes Associated with Terms)	*p*-Value	Adjusted *p*-Value	No of Genes (% Genes Associated with Terms)	*p*-Value	Adjusted *p*-Value
GO:0007623	Circadian rhythm	7 (3.60%)	0.0016	0.0439	13 (6.67%)	3.97 × 10^−5^	0.0028
GO:0006955	Immune response	11 (0.54%)	0.0227	0.0902	27 (1.28%)	0.0021	0.0417
GO:0045087	Innate immune response	No enrichment			17 (1.86%)	4.73 × 10^−2^	0.0194
GO:0006954	Inflammatory response	9 (1.17%)	0.0022	0.0261	17 (2.21%)	6.59 × 10^−6^	8.14 × 10^−4^
GO:0006950	Response to stress	35 (0.91%)	3.09 × 10^−6^	3.09 × 10^−4^	69 (1.79%)	5.79 × 10^−8^	2.68 × 10^−5^
GO:0001666	Response to hypoxia	16 (5.30%)	1.47 × 10^−9^	9.49 × 10^−7^	No enrichment		
GO:0055114	Oxidation-reduction process	13 (1.19%)	0.0457	0.1269	22 (2.01%)	0.0322	0.0462
GO:0000302	Response to reactive oxygen species	8 (3.79%)	2.76 × 10^−4^	0.0065	7 (3.32%)	0.0372	0.1728
GO:0006956	Complement activation	5 (2.65%)	9.47 × 10^−5^	0.0032	8 (4.23%)	1.67 × 10^−7^	4.69 × 10^−5^
GO:0071345	Cellular response to cytokine stimulus	8 (0.79%)	1.10 × 10^−4^	0.0034	15 (1.48%)	0.0250	0.0204
GO:0019221	Cytokine-mediated signaling pathway	No enrichment			11 (1.53%)	0.0117	0.0998
GO:0050900	Leukocyte migration	No enrichment			7 (1.46%)	0.0258	0.0446
GO:0032496	Response to lipopolysaccharide	No enrichment			11 (3.45%)	0.0273	0.0478
GO:0008219	Cell death and apoptosis	13 (0.60%)	0.0307	0.0437	27 (1.24%)	0.0135	0.0148
GO:0009611	Response to wounding	No enrichment			17 (2.68%)	1.06 × 10^−4^	0.0054
GO:0006952	Defense response	23 (1.39%)	3.13 × 10^−4^	0.0071	36 (2.18%)	5.73 × 10^−6^	7.59 × 10^−4^
GO:0006631	Fatty acid metabolic process	No enrichment			9 (2.49%)	0.0298	0.0453
hsa0415	PI3K-AKT signaling pathway	5 (1.41%)	0.0096	3.08 × 10^−6^	5 (1.41%)	0.0366	0.0389

**Table 5:** Differentially expressed genes targeted by the glucocorticoid receptor (GR) in the liver. The target genes of GR were retrieved from TransFac, Harmonizome, GeneGlobe and TRRUST databases and compared to the DEGs of the liver. A total of 64 hepatic DEGs are considered bona fide targets of GR and are compiled based on their biological functions. * Differentially expressed genes (Foldchange > ±1.5, *p*-value < 0.05 and FDR < 0.05).

Gene Symbol	Gene Description	Fold Change ± SD			
		LD_Liver	HD_Liver	LD_Kidney	HD_Kidney
**Circadian rhythm**					
ARNTL/BMAL1	Aryl hydrocarbon receptor nuclear translocator like	2.07 ± 0.46 *	2.69 ± 0.1 *	1.48 ± 0.35	1.9 ± 0.24 *
BHLHE41	Basic helix-loop-helix family member e41	−1.52 ± 0.29 *	−2.41 ± 0.32 *	−1.1 ± 0.2	−1.51 ± 0.09
CRY1	Cryptochrome 1	4.13 ± 1.23 *	4.28 ± 0.51 *	1.62 ± 0.32 *	1.82 ± 0.53 *
DBP	D-box binding PAR bZIP transcription factor	−1.83 ± 0.35 *	−2.41 ± 0.65 *	−1.38 ± 0.33	−1.55 ± 0.08 *
NPAS2	Neuronal PAS domain protein 2	1.3 ± 0.2	2.46 ± 0.51 *	1.15 ± 0.21	1.47 ± 0.18
NR1D2/REVERBbeta	Nuclear receptor subfamily 1 group D member 2	−2.79 ± 0.77 *	−5.25 ± 1.01 *	−1.99 ± 0.56 *	−1.99 ± 0.77 *
PER2	Period circadian clock 2	−1.38 ± 0.69	−2.6 ± 1.36 *	−1.56 ± 0.32 *	−1.85 ± 0.29 *
RORC	RAR related orphan receptor C	1.76 ± 0.26 *	1.41 ± 0.08	−1.02 ± 0.11	1.01 ± 0.05
**Immune and inflammatory response**					
BIRC3	Baculoviral IAP repeat containing 3	1.52 ± 0.26 *	1.64 ± 0.23 *	1.03 ± 0.11	1.16 ± 0.09
CD27	CD27 molecule	−1.41 ± 0.15	−1.53 ± 0.15 *	1.23 ± 0.09	1.04 ± 0.03
CSF2RB	Colony stimulating factor 2 receptor beta	1.51 ± 0.92 *	1.51 ± 0.11 *	1.25 ± 0.53	1.07 ± 0.17
DDX58	DExD/H-box helicase 58	−1.06 ± 0.17	−1.57 ± 0.07 *	−1.13 ± 0.19	−1.27 ± 0.16
IL5	Interleukin 5	−1.1 ± 0.25	−1.52 ± 0.15 *	−1.04 ± 0.05	−1.04 ± 0.07
LYZ	Lysozyme	2.78 ± 1.93 *	5.89 ± 1.04 *	5.31 ± 5.17	3.27 ± 0.93 *
MAPKAPK3	Mitogen-activated protein kinase-activated protein kinase 3	1.16 ± 0.23	1.55 ± 0.11 *	1.08 ± 0.16	1.08 ± 0.07
PDE4B	Phosphodiesterase 4B	1.32 ± 0.41	1.59 ± 0.07 *	−1.1 ± 0.08	1.31 ± 0.22
**Drug metabolic process**					
CYP1A1	Cytochrome P450 family 1 subfamily A member 1	4.86 ± 3.71	2.66 ± 2.83	17.05 ± 25.15	1.75 ± 0.72 *
CYP2C19	Cytochrome P450 family 2 subfamily C member 19	−1.59 ± 0.2 *	−1.51 ± 0.28 *	−1.14 ± 0.15	1.04 ± 0.03
CYP2C42 (CYP2C9)	Cytochrome P450 family 2 subfamily C member 9	−1.67 ± 1.11	−2.05 ± 0.27 *	1.01 ± 0.01	1.54 ± 1.01
CYP3A29 (CYP3A4)	Cytochrome P450 family 3 subfamily A member 4	−1.24 ± 0.12	−1.57 ± 0.28 *	−1.27 ± 0.14	−1.62 ± 0.67
**Cellular response to stress**					
ANGPTL4	Angiopoietin like 4	−1.58 ± 0.85 *	−1.54 ± 0.29 *	−1.32 ± 0.13	−1.01 ± 0.04
CALU	Calumenin	1.18 ± 0.19	1.55 ± 0.25 *	1.01 ± 0.15	1.02 ± 0.09
HSP90AA1	Heat shock protein 90 alpha family class A member 1	1.58 ± 0.2 *	2.01 ± 0.3 *	−1.25 ± 0.17	−1.15 ± 0.24
MAOB	Monoamine oxidase B	−1.32 ± 0.3	−1.86 ± 0.22 *	−1.04 ± 0.1	1.05 ± 0.07
MAT1A	Methionine adenosyltransferase 1A	−1.06 ± 0.24	−1.53 ± 0.22 *	−1.14 ± 0.19	−1.04 ± 0.02
**Oxidation-reduction process**					
ALOX5AP	Arachidonate 5-lipoxygenase activating protein	1.33 ± 0.36	1.87 ± 0.31 *	1.8 ± 1.24	1.41 ± 0.26
APOA2	Apolipoprotein A2	−1.31 ± 0.13	−1.57 ± 0.07 *	−1.03 ± 0.1	−1.07 ± 0.05
CREG1	Cellular repressor of E1A stimulated genes 1	−1.11 ± 0.1	−1.57 ± 0.17 *	1.07 ± 0.15	1.05 ± 0.04
DAO	D-amino acid oxidase	1 ± 0.99	−2 ± 0.93 *	−1.09 ± 0.19	−1.36 ± 0.06
GRHPR	Glyoxylate and hydroxypyruvate reductase	−1.26 ± 0.46	−1.61 ± 0.18 *	−1.13 ± 0.05	−1.1 ± 0.08
PAM	Peptidylglycine alpha-amidating monooxygenase	−1.18 ± 0.19	−1.5 ± 0.29 *	1.19 ± 0.16	−1.07 ± 0.19
**Interferon-gamma-mediated signaling pathway**					
B2M	Beta-2-microglobulin	−1.21 ± 0.25	−1.53 ± 0.34 *	−1.03 ± 0.07	−1.03 ± 0.03
MT2A	Metallothionein 2A	1.26 ± 0.38	1.65 ± 0.37 *	1.85 ± 0.79 *	1.85 ± 1.55
SP100	SP100 nuclear antigen	1.08 ± 0.15	1.74 ± 0.57 *	−1.09 ± 0.12	1.31 ± 0.18
**Cell cycle and arrest**					
CDKN1C	Cyclin dependent kinase inhibitor 1C	1.8 ± 0.69 *	2.74 ± 1.11 *	1.43 ± 0.35	1.2 ± 0.08
GADD45G	growth arrest and DNA damage inducible gamma	2.09 ± 1.65 *	1.75 ± 0.29 *	1.29 ± 0.03	1.42 ± 0.71
GAS1	Growth arrest specific 1	1.09 ± 0.22	−1.51 ± 0.32 *	−1.24 ± 0.08	−1.35 ± 0.23
TOP2A	Topoisomerase (DNA) II alpha	−1.62 ± 0.49 *	−1.77 ± 0.33 *	1.79 ± 1.14	−1.02 ± 0.09
**Response to glucocorticoid stimulus and metabolism**					
LMO3	LIM domain only 3	−1.1 ± 0.09	−1.6 ± 0.38 *	−1.18 ± 0.1	−1.22 ± 0.18
NR3C1	Nuclear receptor subfamily 3 group C member 1	1.57 ± 0.08 *	1.22 ± 0.06	−1.15 ± 0.12	−1.21 ± 0.28
SERPINA6	Serpin family A member 6	−1.6 ± 0.57 *	−1.54 ± 0.33 *	−1.15 ± 0.05	−1.13 ± 0.11
STC1	Stanniocalcin 1	1.54 ± 0.3 *	1.58 ± 0.35 *	−1.15 ± 0.16	1.02 ± 0.02
TAT	Tyrosine aminotransferase	1.11 ± 0.35	−2.15 ± 0.45 *	−1.13 ± 0.05	−1.13 ± 0.19
**Lipid metabolism**					
AFP	Alpha fetoprotein	−1.57 ± 0.36 *	−1.33 ± 0.13	1.11 ± 0.29	2.12 ± 1.01 *
LPIN1	Lipin 1	−1.09 ± 0.12	−1.55 ± 0.35 *	−1.19 ± 0.06	−1.08 ± 0.07
**Aminoacid synthesis**					
PHGDH	Phosphoglycerate dehydrogenase	−1.42 ± 0.56	−2.49 ± 1.05 *	−1.03 ± 0.05	−1.31 ± 0.19
**Carbohydrate metabolic process**					
XYLB	Xylulokinase	−1.25 ± 0.38	−1.52 ± 0.35 *	−1.52 ± 0.27 *	−1.60 ± 0.27 *
**Transmembrane transport**					
ABCA8	ATP binding cassette subfamily A member 8	1.05 ± 0.22	−1.55 ± 1.03 *	−1.44 ± 0.34	−1.47 ± 0.31
ATP1A2	ATPase Na+/K+ transporting subunit alpha 2	−1.34 ± 0.54	−1.69 ± 0.26 *	1.22 ± 0.19	1.35 ± 0.18
ATP1B1	ATPase Na+/K+ transporting subunit beta 1	1.21 ± 0.35	1.51 ± 0.37 *	1.02 ± 0.02	1.02 ± 0.03
ATP9A	ATPase phospholipid transporting 9A	2.35 ± 0.35 *	1.6 ± 0.22 *	1.3 ± 0.11	1.3 ± 0.29
**Extracellular matrix organization**					
ANXA2	Annexin A2	−1.42 ± 0.66	−1.78 ± 0.27 *	1.46 ± 0.62	1.09 ± 0.11
FMOD	Fibromodulin	−1.2 ± 0.23	−1.52 ± 0.39 *	−1.07 ± 0.07	−1.18 ± 0.12
ITGA8	Integrin subunit alpha 8	1.24 ± 0.27	1.51 ± 0.03 *	−1 ± 0.12	1.24 ± 0.39
SPP1	Secreted phosphoprotein 1	−1.53 ± 0.13 *	−1.17 ± 0.67	3 ± 3.56	1.38 ± 0.28
**Signal transduction**					
NR1I3 (CAR)	Nuclear receptor subfamily 1 group I member 3	1.51 ± 0.34 *	1.77 ± 0.1 *	1.07 ± 0.09	−1.01 ± 0.08
PLEK	Pleckstrin	1.21 ± 0.69	1.51 ± 0.12 *	1.62 ± 0.66 *	1.56 ± 0.17 *
REEP5	Receptor accessory protein 5	−1 ± 0.29	1.79 ± 0.42 *	1.04 ± 0.14	1.02 ± 0.1
**Protein complex assembly and cell adhesion**					
CLDN14	Claudin 14	−1.34 ± 0.57	−1.69 ± 0.32 *	1.13 ± 0.07	1.44 ± 0.82
CNTN4	Contactin 4	1.09 ± 0.35	−1.95 ± 0.52 *	1.05 ± 0.01	1.37 ± 0.25
MOGS	Mannosyl-oligosaccharide glucosidase	1.25 ± 0.28	1.68 ± 0.28 *	1.25 ± 0.12	1.25 ± 0.26
SERPINI1	Serpin family I member 1	−1.52 ± 0.18 *	−1.01 ± 0.09	−1.22 ± 0.08	−1.02 ± 0.04
**Transcriptional regulation**					
RPL32	Ribosomal protein L32	1.32 ± 0.22	1.56 ± 0.35 *	1.03 ± 0.11	1 ± 0.01
YIPF1	Yip1 domain family member 1	−1.08 ± 0.22	−1.51 ± 0.17 *	−1.03 ± 0.04	−1.27 ± 0.03

**Table 6:** Drug-induced steatosis-regulated genes in the liver and kidney. Based on mechanistically linked and lipid-droplet-associated gene regulations, a total of 65 DEGs were identified after diclofenac treatment and categorized based on their biological processes. * Differentially expressed genes (Foldchange > ±1.5, *p*-value < 0.05 and FDR < 0.05).

Probeset ID	Gene Symbol	Description	Liver		Kidney	
			LD	HD	LD	HD
			Fold Change (Average) ± SD			
**Lipogenesis**						
Ssc.4891.1.A1_at	AGPAT9 (GPAT3)	Glycerol-3-phosphate acyltransferase 3	1.14 ± 0.39	2.99 ± 1.11 *	1.16 ± 0.29	1.08 ± 0.02
Ssc.12241.1.A1_at	ANXA2	Annexin A2	−1.42 ± 0.66	−1.78 ± 0.27 *	1.46 ± 0.62	1.09 ± 0.11
Ssc.3703.1.S1_at	APOA2	Apolipoprotein A2	−1.31 ± 0.13	−1.57 ± 0.07 *	−1.03 ± 0.1	−1.07 ± 0.05
Ssc.14503.1.S1_at	APOA4	Apolipoprotein A4	1.16 ± 4.9	1.56 ± 0.29 *	1.01 ± 0.08	1.07 ± 0.16
Ssc.1039.1.S1_at	APOC3	Apolipoprotein C3	−1.01 ± 0.01	−1.03 ± 0.04	−3.97 ± 1.42 *	1.01 ± 0.11
Ssc.5848.1.S1_at	B4GALT5	Beta-1,4-galactosyltransferase 5	1.98 ± 1.28	1.51 ± 0.35 *	1.58 ± 0.76 *	1.15 ± 0.14
Ssc.2224.1.S1_at	CYP2C19	Cytochrome P450 family 2 subfamily C member 19	−1.59 ± 0.2 *	−1.51 ± 0.28 *	1.11 ± 0.15	−1.04 ± 0.11
Ssc.19298.2.S1_at	DHCR24	24-dehydrocholesterol reductase	−1.01 ± 0.39	1.01 ± 0.12	1.36 ± 0.2	1.56 ± 0.03 *
Ssc.30467.1.A1_at	ELOVL2	ELOVL fatty acid elongase 2	2.66 ± 2.9	3.53 ± 0.59 *	1.04 ± 0.03	−1.18 ± 0.08
Ssc.1595.1.S1_a_at	FADS1	Fatty acid desaturase 1	−1.64 ± 3.48	−1.91 ± 0.79 *	1 ± 0.1	1.55 ± 0.06 *
Ssc.18175.1.A1_at	FASN	Fatty acid synthase	−1.61 ± 0.22 *	−2.12 ± 0.54 *	1.03 ± 0.04	1.14 ± 0.11
Ssc.4417.1.A1_at	GALNT2	Polypeptide N-acetylgalactosaminyltransferase 2	1.55 ± 0.77 *	1.45 ± 0.1	1.01 ± 0.02	−1.14 ± 0.05
Ssc.11103.1.S1_at	MDH1	Malate dehydrogenase 1	1.04 ± 0.06	1.54 ± 0.07 *	1.01 ± 0.03	1.09 ± 0.09
Ssc.3345.2.S1_at	MVK	Mevalonate kinase	−1.53 ± 1.07	−2.36 ± 0.93 *	1.05 ± 0.07	−1.08 ± 0.07
Ssc.9781.1.S1_at	SERPINE1	Serpin family E member 1	1.87 ± 0.95 *	1.58 ± 0.04 *	3.32 ± 3.93	1.41 ± 0.53
Ssc.4552.1.S1_at	VLDLR	Very low density lipoprotein receptor	−1.53 ± 0.21 *	1.98 ± 0.15 *	1.08 ± 0.06	1.08 ± 0.02
**Fatty acid oxidation/mitochondrial stress**						
Ssc.2176.1.A1_at	ACSL3	Acyl-CoA synthetase long-chain family member 3	−1.52 ± 0.83 *	−1.66 ± 0.58 *	1.03 ± 0.08	1.07 ± 0.06
Ssc.18933.1.A1_at	ADH4	Alcohol dehydrogenase 4	−1.72 ± 0.86 *	−2.93 ± 1.27 *	1.01 ± 0.08	−1.06 ± 0.11
Ssc.17183.1.S1_at	ATP5D	ATP synthase subunit delta, mitochondrial-like	1.28 ± 0.06	1.56 ± 0.36 *	1.08 ± 0.13	1.1 ± 0.04
Ssc.26021.1.S1_at	FOXA1	Forkhead box A1	1.6 ± 0.55 *	1.79 ± 0.37 *	1.12 ± 0.17	1.01 ± 0.05
**Lipid transport**						
Ssc.13622.1.S1_at	AQP4	Aquaporin 4	−1.56 ± 0.24 *	−1.14 ± 0.15	1 ± 0.02	1.09 ± 0.17
Ssc.604.1.S1_at	FABP1	Fatty acid binding protein 1, liver	−1.12 ± 0.56	1.01 ± 0.11	1.59 ± 0.18 *	1.32 ± 0.29
Ssc.6605.1.S1_at	HDLBP	High density lipoprotein binding protein	1.35 ± 0.18	1.64 ± 0.28 *	−1.18 ± 0.18	1.03 ± 0.08
Ssc.21926.1.S1_at	LDLR	Low density lipoprotein receptor	−1.11 ± 0.51	−1.13 ± 0.13	1.52 ± 0.2 *	1.59 ± 0.1 *
Ssc.29043.1.S1_at	NR5A2	Nuclear receptor subfamily 5, group A, member 2	−1.3 ± 0.64	−1.57 ± 0.46 *	1.1 ± 0.1	−1.31 ± 0.3
**LD growth/ER stress**						
Ssc.29181.1.A1_s_at	ATP2A2 (SERCA2)	ATPase sarcoplasmic/endoplasmic reticulum Ca^2+^ transporting 2	1.44 ± 0.31	2.04 ± 1.08 *	1.01 ± 0.04	1.11 ± 0.14
Ssc.3106.1.S1_at	CALR	Calreticulin	1.35 ± 0.25	2.42 ± 1.21 *	1.32 ± 0.14	1.79 ± 0.37 *
Ssc.12842.1.S1_at	CAV1	Caveolin 1	−2.05 ± 0.39 *	−1.74 ± 0.38 *	1.2 ± 0.27	−1.2 ± 0.08
Ssc.250.1.S1_at	CBR1	Carbonyl reductase 1	−1.97 ± 0.69 *	−1.6 ± 1.08	−1.49 ± 0.64	−1.09 ± 0.08
Ssc.6784.1.S1_at	LIPE	Lipase E	1.04 ± 0.09	−1.23 ± 0.31	1.14 ± 0.13	1.57 ± 0.29 *
Ssc.6323.1.S1_at	PLIN2	Perilipin 2	3.01 ± 1.39 *	4.7 ± 1.15 *	1.06 ± 0.12	1.32 ± 0.28
Ssc.17391.2.S1_at	RAB35	RAB35, member RAS oncogene family	−1.2 ± 0.38	−1.57 ± 0.38 *	1.08 ± 0.07	−1.26 ± 0.27
Ssc.29036.1.S1_at	TUBA4A	Tubulin, alpha 4a	1.59 ± 0.61 *	1.62 ± 0.43 *	1.2 ± 0.14	1.26 ± 0.03
**Oxidative stress/signalling events**						
Ssc.22673.1.S1_at	AKR1C1	Aldo-keto reductase family 1, member C1	−1.16 ± 0.03	−1.81 ± 0.31 *	−1.3 ± 0.45	−1.7 ± 0.31 *
Ssc.8987.1.A1_at	ARHGAP1	Rho GTPase activating protein 1	1.62 ± 0.12 *	1.7 ± 0.17 *	1.27 ± 0.18	1.18 ± 0.19
Ssc.27539.2.A1_a_at	ASPH	Aspartate beta-hydroxylase	1.13 ± 0.15	1.57 ± 0.14 *	1.08 ± 0.08	1.11 ± 0.06
Ssc.22002.2.A1_at	CXCL13	Chemokine lignad 13	1.11 ± 0.14	1.54 ± 0.16 *	−1.07 ± 0.14	1.26 ± 0.22
Ssc.20578.1.S1_at	CXCL14	Chemokine ligand 14	1.01 ± 0.05	1.24 ± 0.16	1.61 ± 0.68 *	1.58 ± 0.4 *
Ssc.19692.1.S1_at	CXCL2	Chemokine ligand 2	4.33 ± 1.26 *	2.56 ± 1.05 *	4.05 ± 5.08	1.76 ± 0.56 *
Ssc.390.1.A1_at	HIF1A	Hypoxia inducible factor 1 alpha subunit	−1.67 ± 0.04 *	−1.08 ± 0.18	1.16 ± 0.09	1.11 ± 0.11
Ssc.12191.1.A1_at	HSP90AA1	Heat shock protein 90kDa	1.58 ± 0.2 *	1.32 ± 0.3	−1.25 ± 0.17	−1.15 ± 0.24
Ssc.308.1.S1_at	HSP90B1	Heat shock protein 90 beta family member 1	1.26 ± 0.31	1.93 ± 0.8 *	−1.6 ± 0.4 *	−1.64 ± 0.12 *
Ssc.9056.1.A1_at	HSPA5	Heat shock protein family A (Hsp70) member 5	1.66 ± 0.3 *	1.81 ± 0.1 *	1.16 ± 0.21	1.2 ± 0.04
Ssc.23054.1.S1_at	JAK3	Janus kinase 3	1.27 ± 0.41	1.52 ± 0.19 *	1.42 ± 0.49	1.34 ± 0.13
Ssc.18557.1.S1_at	KNG1	Kininogen 1	−1.2 ± 0.04	−1.61 ± 0.15 *	−1.96 ± 1 *	−2.13 ± 0.14 *
Ssc.7297.1.S1_at	MAOB	Monoamine oxidase B	−1.32 ± 0.3	−1.86 ± 0.22 *	−1.04 ± 0.1	1.05 ± 0.07
Ssc.7478.1.A1_at	MARCH5	Membrane associated ring-CH-type finger 5	−1.18 ± 0.07	−1.5 ± 0.29 *	−1.11 ± 0.06	−1.25 ± 0.25
Ssc.10955.1.S1_at	MGST3	Microsomal glutathione S-transferase 3	−1.21 ± 0.25	−1.7 ± 0.36 *	1.21 ± 0.15	1.11 ± 0.09
Ssc.9170.1.A1_at	PRKD1	Protein kinase D1	−2.04 ± 1.13 *	−2.52 ± 0.42 *	1.06 ± 0.1	1.14 ± 0.05
Ssc.830.1.S1_at	PSME2	Proteasome activator subunit 2	−1.14 ± 0.15	−1.51 ± 0.26 *	1.14 ± 0.27	−1.09 ± 0.07
Ssc.14490.1.S1_at	PTGR1	Prostaglandin reductase 1	−1.27 ± 0.68	−1.18 ± 0.22	3.4 ± 2.61 *	3.13 ± 1.91 *
Ssc.3706.1.S2_at	SOD2	Superoxide dismutase 2, mitochondrial	2.22 ± 0.9 *	1.91 ± 0.53 *	1.09 ± 0.14	1.07 ± 0.05
Ssc.11252.1.S1_at	SPR	Sepiapterin reductase	−1.61 ± 0.49 *	−1.67 ± 0.38 *	−1.22 ± 0.05	−1.24 ± 0.02
Ssc.14066.2.S1_at	TAT	Tyrosine aminotransferase	1.11 ± 0.35	−2.15 ± 0.45 *	−1.13 ± 0.05	−1.13 ± 0.19
Ssc.3753.1.S1_at	TFRC (CD71)	Transferrin receptor	1.64 ± 0.69 *	1.34 ± 0.35	1.68 ± 0.31 *	1.44 ± 0.36
Ssc.28413.1.A1_at	UBXN4	UBX domain-protein 4	1.06 ± 0.04	1.12 ± 0.15	−1.52 ± 0.31 *	−1.92 ± 0.24 *
Ssc.16350.1.S1_at	UCP2	Uncoupling protein 2	1.25 ± 0.04	1.17 ± 0.12	1.59 ± 0.22 *	1.78 ± 0.36 *
Ssc.3185.1.S1_at	TXNDC5	Thioredoxin domain-containing protein 5	1.07 ± 0.16	1.53 ± 0.02 *	1.12 ± 0.18	1.28 ± 0.01
**Lipid metabolism marker genes**						
Ssc.16377.1.A1_s_at	GSTA1	Glutathione S-transferase alpha 1	−1.08 ± 0.1	−1.61 ± 1.07	3.23 ± 1.40 *	4.60 ± 1.27 *
Ssc.5991.1.A1_at	KRT18	Keratin 18	1.74 ± 0.62 *	1.67 ± 0.25 *	4.01 ± 5.19	−1.06 ± 0.05
Ssc.5955.1.A1_at	KRT8	keratin 8	1.9 ± 0.38 *	2.6 ± 0.28 *	1.51 ± 0.28 *	1.93 ± 0.11 *
**Glucose metabolism**						
Ssc.8980.1.A1_at	ANGPTL4	Angiopoietin-like 4	−1.58 ± 1.15	−1.54 ± 0.29 *	−1.32 ± 0.13	−1.01 ± 0.04
Ssc.27727.1.S1_at	PGM5	Phosphoglucomutase 5	−1.39 ± 0.33	−1.79 ± 0.11 *	−1.59 ± 0.28 *	−1.48 ± 0.48
Ssc.21431.3.A1_s_at	PHGDH	Phosphoglycerate dehydrogenase	−1.42 ± 0.56	−2.49 ± 1.05 *	−1.03 ± 0.05	−1.31 ± 0.19
Ssc.4717.1.S1_at	PHKB	Phosphorylase b kinase regulatory subunit beta	−1.32 ± 0.34	−1.58 ± 0.16 *	−1.04 ± 0.1	−1.17 ± 0.02

**Table 7:** Commonly regulated genes in the liver and kidney after diclofenac treatment. * Differentially expressed genes (Foldchange > ±1.5, *p*-value < 0.05 and FDR < 0.05).

Gene Symbol	Gene Description	Fold Change (Average) ± SD			
		LD_Liver	HD_Liver	LD_Kidney	HD_Kidney
**Immune and inflammatory response**					
C9	Complement component 9	2.73 ± 0.77 *	1 ± 0.05	−1.81 ± 0.78 *	−1.23 ± 0.32
CD163	CD163 antigen	1.49 ± 0.75	2.04 ± 0.42 *	2.01 ± 1.67 *	1.36 ± 0.45
CXCL2	Chemokine (C-X-C motif) ligand 2	4.33 ± 0.37 *	2.56 ± 1.05 *	4.05 ± 5.08	1.76 ± 0.56 *
GJA1	Gap junction protein, alpha 1	1.63 ± 0.16 *	1.32 ± 0.25	1.86 ± 1.5 *	−1.59 ± 0.19 *
IFI44L	Interferon-induced protein 44-like	−1.18 ± 0.31	−2.03 ± 0.41 *	−1.18 ± 0.08	−1.51 ± 0.31 *
IL10RB	Interleukin 10 receptor, beta	1.9 ± 0.06 *	2.6 ± 0.28 *	1.51 ± 0.28 *	1.93 ± 0.11 *
IRG6 (RSAD2)	Inflammatory response protein 6	−1.82 ± 0.84 *	−3.24 ± 1.23 *	−1.99 ± 0.17 *	−2.75 ± 0.59 *
ISG15	ISG15 ubiquitin-like modifier	−1.2 ± 0.15	1.86 ± 0.11 *	−1.32 ± 0.12	−1.91 ± 0.24 *
KNG1	Kininogen 1	−1.2 ± 0.22	−1.61 ± 0.15 *	−1.96 ± 1 *	−2.13 ± 0.14 *
LYZ	Lysozyme	2.78 ± 1.93 *	5.89 ± 1.04	5.31 ± 5.17	3.27 ± 0.93 *
S100A9	S100 calcium binding protein A9	7.03 ± 5.21	4.13 ± 2.55 *	6.17 ± 8.48	2.58 ± 0.65 *
TFRC	Transferrin receptor (CD71)	1.64 ± 0.69 *	1.34 ± 0.35	1.68 ± 0.31 *	1.44 ± 0.36
TNFAIP6	Tumor necrosis factor, alpha-induced protein 6	−1.58 ± 0.27 *	−1 ± 0.13	−2.09 ± 0.39 *	−1.18 ± 0.28
USP18	Ubiquitin specific peptidase 18	−1.39 ± 0.53	−2.25 ± 0.11 *	−1.52 ± 0.08 *	−1.68 ± 0.47 *
VCAM1	Vascular cell adhesion molecule 1	−1.21 ± 0.07	1.85 ± 1.1 *	1.87 ± 1.1 *	1.57 ± 0.63 *
**Drug metabolism**					
CYB5D2	Cytochrome B5 domain containing 2	−1.07 ± 0.45	1.8 ± 0.29 *	1.15 ± 0.37	1.56 ± 0.24 *
CYP3A29	Cytochrome P450 3A29	−1.24 ± 0.25	−1.57 ± 0.28 *	−1.27 ± 0.14	−1.62 ± 0.67 *
**Response to stress**					
IGFBP2	Insulin-like growth factor binding protein 2	−1.37 ± 0.29	−1.85 ± 0.37 *	1.52 ± 0.28 *	1.26 ± 0.09
RBM3	RNA binding motif (RNP1, RRM) protein 3	−1.51 ± 0.15	−1.93 ± 0.12 *	−1.52 ± 0.27 *	−2.56 ± 0.55 *
**Oxidation-reduction process**					
ACAD10	Acyl-CoA dehydrogenase family, member 10	1.23 ± 0.46	1.93 ± 0.35 *	1.19 ± 0.44	2.14 ± 0.1 *
AKR1C1	Aldo-keto reductase family 1, member C1	−1.81 ± 0.03 *	−1.81 ± 0.31 *	−1.3 ± 0.45	−1.7 ± 0.31 *
PIR	Pirin-like	−1.15 ± 0.77	−1.53 ± 0.34 *	2.1 ± 0.83 *	1.25 ± 0.08
**Response to hypoxia**					
ANGPT1	Angiopoietin 1	−1.15 ± 0.19	−1.51 ± 0.39 *	−1.56 ± 0.37 *	−1.14 ± 0.22
**Cell death and apoptosis**					
CALR	Calreticulin	1.35 ± 0.25	2.42 ± 1.21 *	1.32 ± 0.14	1.79 ± 0.37 *
EVA1A	Protein FAM176A-like	−1.54 ± 3.19	−2.06 ± 0.14 *	−1.44 ± 0.79	−1.82 ± 0.52 *
HRG	Histidine-rich glycoprotein	−1.52 ± 0.38 *	−2.26 ± 0.53 *	−1.16 ± 0.28	−1.75 ± 0.34 *
HSP90B1	Heat shock protein 90kDa beta, member 1	1.34 ± 0.31	1.93 ± 0.80 *	−1.59 ± 0.40 *	−1.64 ± 0.12 *
**Circadian rhythmic process**					
ARNTL/BMAL1	Aryl hydrocarbon receptor nuclear translocator-like	2.07 ± 0.46 *	2.69 ± 0.1 *	1.48 ± 0.35	1.9 ± 0.24 *
BHLHE41	Basic helix-loop-helix family, member e41	−1.52 ± 1.23 *	−2.41 ± 0.32 *	−1.1 ± 0.2	−1.51 ± 0.09 *
CRY1	Cryptochrome 1	4.13 ± 1.23 *	4.28 ± 0.51 *	1.62 ± 0.32 *	1.82 ± 0.53 *
NPAS2	Neuronal PAS domain protein 2	1.3 ± 0.1	2.46 ± 0.51 *	1.15 ± 0.21	1.57 ± 0.18 *
NR1D2/REVERBB	Nuclear receptor subfamily 1, group D, member 2	−2.79 ± 0.38 *	−5.25 ± 1.01 *	−1.99 ± 0.56 *	−1.99 ± 0.77 *
PER2	Period homolog 2	−1.38 ± 0.2	−2.6 ± 1.36 *	−1.56 ± 0.32 *	−1.85 ± 0.29 *
**Lipid metabolism**					
ARSD	Arylsulfatase D	−1.05 ± 0.23	−1.73 ± 0.11 *	−1.12 ± 0.18	−1.73 ± 0.15 *
KRT8	keratin 8	1.9 ± 0.38 *	2.6 ± 0.28 *	1.51 ± 0.28 *	1.93 ± 0.11 *
PLEK	Pleckstrin	1.21 ± 0.69	1.5 ± 0.12 *	1.62 ± 0.66 *	1.56 ± 0.17 *
**Protein metabolism**					
ANKRD28	Ankyrin repeat domain 28	1.09 ± 0.14	1.51 ± 0.12 *	−1.13 ± 0.23	−1.52 ± 0.07 *
ASRGL1	L-asparaginase-like	−1.19 ± 0.29	−1.53 ± 0.07 *	−1.38 ± 0.08	−1.63 ± 0.14 *
GTF2H3	General transcription factor IIH, polypeptide 3	1.07 ± 0.19	1.82 ± 0.05 *	−1.07 ± 0.07	1.59 ± 0.15 *
IGFBP3	Insulin-like growth factor binding protein 3	−1.41 ± 0.04	−1.65 ± 0.13 *	−1.83 ± 0.98	−2.47 ± 0.7 *
**Carbohydrate metabolism**					
XYLB	Xylulose kinase-like	−1.25 ± 0.3	−1.52 ± 0.35 *	−1.52 ± 0.27 *	−1.60 ± 0.27 *
**Signal transduction**					
GPNMB	Glycoprotein (transmembrane) nmb	−1.1 ± 0.19	−2.01 ± 0.35 *	2.48 ± 0.39 *	2.47 ± 0.85 *
RND3	Rho family GTPase 3	1.32 ± 0.15	1.80 ± 0.02 *	1.27 ± 0.15	1.55 ± 0.24 *

**Table 8:** Master regulatory genes in the liver and kidney after low- and high-dose diclofenac treatments. The key master molecules were identified and their regulatory networks were constructed using the GeneXplain software. Given is a summary of master regulatory genes and the associated networks with the number of total interacting genes and DEGs, network score, Z-score and average fold change. The filtering criteria Z- Score > 1 and Score > 0.2 were set to select statistically significant master regulators.

Master Regulatory Genes	No of Genes		Score	FDR	Z-Score	Fold Change (Average) ± SD
	Total No. of Genes in the Network	Statistically Significant DEGs				
**DCF_low dose_liver**						
Cryptochrome 1 (CRY1)	51	27	0.27785	0.02	2.8496	4.13 ± 1.23
Hypoxia inducible factor 1, alpha subunit (HIF1A)	76	39	0.47052	0.011	1.90028	−1.67 ± 0.04
Nuclear receptor subfamily 3, group C, member 1 (NR3C1)	90	42	0.58276	0.047	1.48431	1.57 ± 0.08
**DCF_high dose_liver**						
Cryptochrome 1 (CRY1)	113	66	0.23593	0.024	3.41268	4.28 ± 0.51
Insulin-like growth factor binding protein 2 (IGFBP2)	196	111	0.36927	0.016	3.01892	−1.85 ± 0.37
Lipopolysaccharide-induced TNF factor (LITAF)	199	111	0.40046	0.027	2.20201	1.81 ± 0.22
Angiopoietin-like 4 (ANGPTL4)	130	77	0.22103	0.028	2.10328	−1.54 ± 0.29
**DCF_low dose_kidney**						
Dipeptidyl-peptidase 4 (DPP4)	83	40	0.50686	0.005	2.69108	−1.52 ± 0.51
Angiopoietin 1 (ANGPT1)	43	25	0.25006	0.011	2.86388	−1.56 ± 0.37
Sirtuin 1 (SIRT1)	77	38	0.57072	0.021	1.51382	−1.51 ± 0.21
**DCF_high dose_kidney**						
Matrix metallopeptidase 7 (MMP7)	103	52	0.44009	0.007	3.18595	2.99 ± 0.75
Complement component 1, q subcomponent, alpha polypeptide (C1QA)	102	49	0.47405	0.002	3.33235	1.51 ± 0.2
Nuclear factor of kappa light polypeptide gene enhancer in B cells inhibitor, zeta (NFKBIZ)	114	56	0.37981	0.02	2.35022	1.59 ± 0.2
Insulin-like growth factor binding protein 3 (IGFBP3)	113	56	0.48865	0.029	1.92918	−2.47 ± 0.7

**Table 9:** Antibodies used for immunohistochemistry.

Antibody	Vendor	Cat no.	Lot Number	Dilution	Antigen Retrieval
Cortisol Binding Globulin (CBG)	Abcam (Cambridge, UK)	ab107368	GR74750-20	1:100	pH 6
CPS1 / Hepar-1	Santa Cruz Biotechnology, (Heidelberg, Germany)	sc-58693	C1213	1:7500	pH 6
CRF1	Abcam	ab150561	GR139717-15	1:250	pH 9
CYP1A1	Abcam	ab3568		1:200	pH 6
Cryptochrome I (CRY1)	Abcam	ab3518	GR307241-6	1:300	pH 6
ELOVL2	Abcam	ab111162	GR48113-1	1:100	pH 6
Glucocorticoid Receptor (GR)	Abcam	ab233165	GR3222243-1	1:100	pH 9
HIF1a	Abcam	ab463	GR252860	1:40	pH 6
KAT-13D (CLOCK)	Abcam	ab65033	GR88646-1	1:50	pH 6
MPO	Dako (Hamburg, Germany)	REFA0398	20001076	1:500	pH 6
Per2	Abcam	ab200388	GR221389-8	1:300	pH 6
SOD-1	Santa Cruz	sc-11407	B0415	1:500	pH 6
SOD-2	Santa Cruz	sc-30080	J0713	1:250	pH 6

## Data Availability

The data presented in this study are available within the article or its supplementary material.

## Supplementary Materials

The following supporting information can be downloaded at: https://www.mdpi.com/article/10.3390/ijms24021445/s1.

## Abbreviations

**AACS**	acetoacetyl-coenzyme A synthetase
**ACTH**	adrenocorticopic hormone
**ADH4**	alcohol dehydrogenase 4
**ADR**	adverse drug reaction
**AhR**	aryl hydrocarbon receptor
**ALB**	albumin
**ALP**	alkaline phosphatase
**ALT**	alanine aminotransferase
**ANGPTL4**	angiopoietin-like 4
**ARID5A**	AT rich interactive domain 5A
**AST**	aspartate aminotransferase
**BMCA**	B-cell maturation antigen
**BUN**	blood urea nitrogen
**C/EBPβ**	CCAAT/enhancer-binding protein beta
**CAE**	chloroacetate esterase
**CARPA**	complement activation related pseudoallergy
**CAV1**	caveolin-1
**CBG**	corticosteroid binding globulin
**CCL**	C-C motif chemokine ligand
**CDK**	cyclin-dependent kinase
**CBG**	corticosteroid binding globulin
**cGMP**	cyclic guanosine monophosphate
**CHOL**	cholesterol
**C/EBPB**	CCAAT/enhancer-binding protein-homologous protein beta
**CPS**	carbamoyl phosphate synthetase
**CREA**	creatinine
**CRF**	corticotropin-releasing factor
**CRY1**	cryptochrome 1
**CXCL**	C-X-C motif chemokine ligand
**CYP**	cytochrome P450
**CPS1**	carbamoyl-phosphate synthetase 1
**DEFB1**	ß-defensin
**DEGs**	differentially expressed genes
**DILI**	drug induced liver injury
**DPP4**	dipeptidyl-peptidase 4
**ECM**	extracellular matrix
**EDTA-2K**	dipotassium ethylenediaminetetraacetic acid (anticoagulant)
**ELOVL2**	ELOVL fatty acid elongase 2
**ER stress**	endoplasmic reticulum stress
**EvG**	Elastika van Gieson (stain)
**FDR**	false discovery rate
**GGT**	gamma glutamyl transferase
**GLU**	glucose
**GO**	gene ontology
**GR**	glucocorticoid receptor
**GRE**	glucocorticoid response elements
**H&E**	Hematoxylin and Eosin
**HCT**	hematocrit
**HepPar1**	Hepatocyte Paraffin 1 a hepatocyte specific antigen antibody
**HGB**	hemoglobin
**HIF1A**	hypoxia inducible factor 1 alpha subunit
**HPA**	axis- hypothalamus-pituitary-adrenal neuroendocrine axis
**HRG**	histidine rich glycoprotein
**IFN**	interferon
**IGF**	insulin-like growth factor
**IGFBP**	insulin-like growth factor binding protein
**KLF**	krüppel-like family *transcription factor*
**LITAF**	lipopolysaccharide-induced tumor necrosis factor-alpha factor
**LPS**	lipopolysaccharide
**MAC**	membrane attack complex
**MCH**	mean corpuscular hemoglobin
**MCHC**	mean corpuscular hemoglobin concentration
**MCP**	monocyte chemotactic protein
**MCV**	mean corpuscular volume
**MEF2**	myocyte enhancer factor-2
**MeV**	Multi Experimental Viewer
**MIP**	macrophage inflammatory protein
**MMP7**	matrix metallopeptidase 7
**MPO**	myeloperoxidase
**NFKBIZ**	nuclear factor of kappa light polypeptide gene enhancer in B cells inhibitor zeta
**NK**	natural killer cells
**NMDA**	N-methyl-D-aspartate
**NSAID**	nonsteroidal anti-inflammatory drug
**PAS**	Periodic acid-Schiff reaction
**PER2**	period circadian regulator 2
**PLIN2**	perilipin 2
**PLT**	platelet
**PML**	polymorphonuclear leukocytes
**PPAR**	peroxisome proliferator-activated receptor
**PPI**	protein-protein interaction
**PWM**	positional weight matrices
**RBC**	red blood cells
**RET**	reticulocyte
**RGC-32**	response gene to complement 32
**ROS**	reactive oxygen species
**RT-PCR**	reverse transcriptase-polymerase chain reaction
**SELL**	L-selectin
**SIRT1**	sirtuin 1
**SOD**	superoxide dismutase 1
**SPF**	specific pathogen free
**TBIL**	total bilirubin
**TFBS**	transcription factor binding site
**TG**	triglycerides
**TNF**	tumor necrosis factor
**TP**	total protein
**TSS**	transcription start sites
**VLDLR**	very low-density lipoprotein receptor
**WBC**	white blood cells
