# Supplementary material for: Diclofenac Disrupts the Circadian Clock and through Complex Cross-Talks Aggravates Immune-Mediated Liver Injury—A Repeated Dose Study in Minipigs for 28 Days
Source: Int J Mol Sci. 2023 Jan 11;24(2):1445. doi: 10.3390/ijms24021445 (PMC9863319; doi:10.3390/ijms24021445)
Supplement: Supplementary file 1 [file ijms-24-01445-s001.zip › Supplementary Materials.pdf]

SUPPLEMENTARY MATERIALS

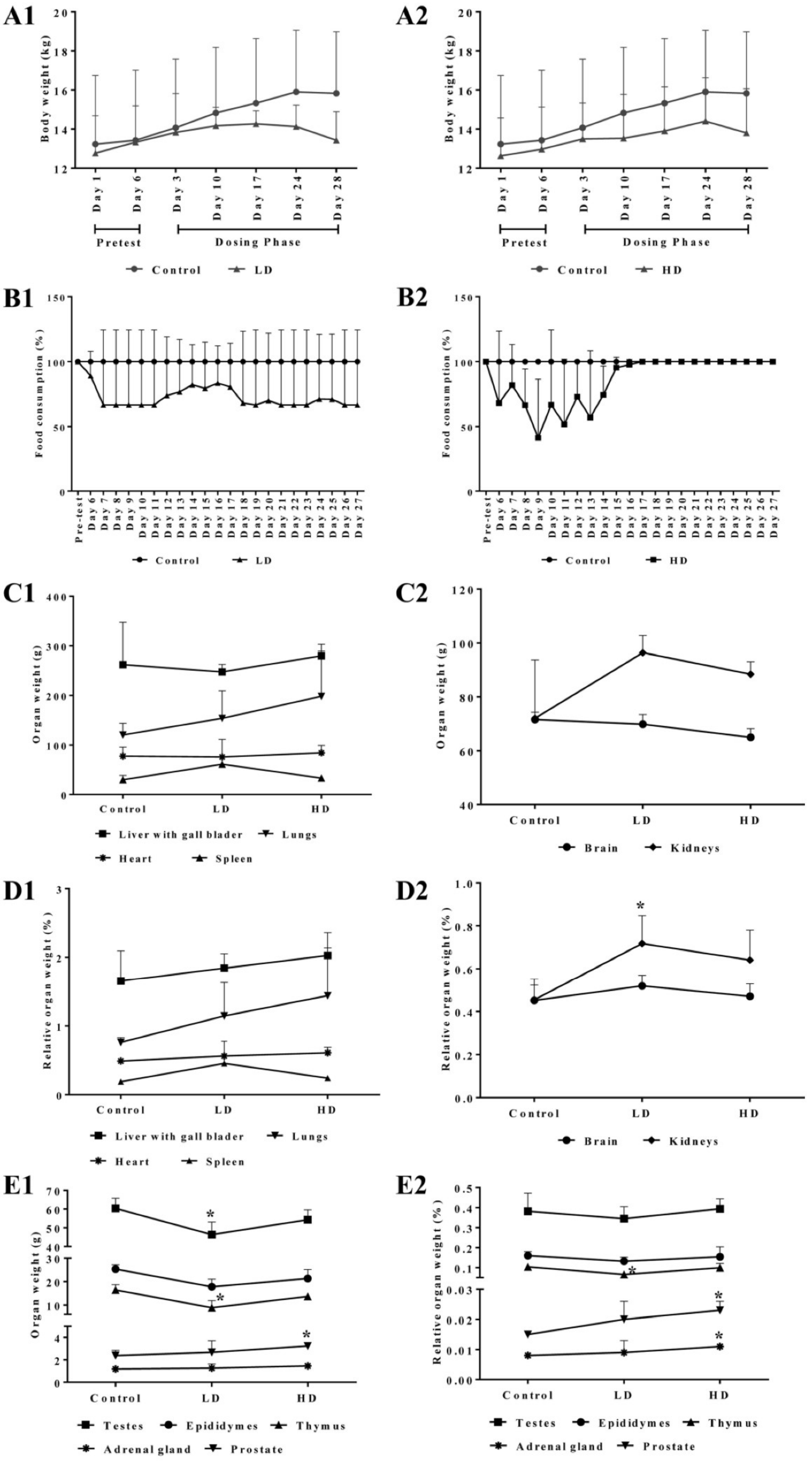
A1
A2
Body weight (kg)
Day 1
Day 6
Day 3
Day 10
Day 17
Day 24
Day 28
Pretest
Dosing Phase
Control
LD
HD
B1
B2
Food consumption (%)
Pre-test
Control
LD
HD
C1
C2
Organ weight (g)
Liver with gall blader
Lungs
Heart
Spleen
Brain
Kidneys
D1
D2
Relative organ weight (%)
E1
E2
Testes
Epididymes
Thymus
Adrenal gland
Prostate

Supplementary Figure S1: **Body weight and food consumption after repeated diclofenac treatment for 28 days**. **Panel A:** Body weight at low-dose (A1) and high-dose treatment (A2). **Panel B:** Food consumption at low-dose (B1) and high-dose treatment (B2). **Panel C and D**: Selective organ weights. **Panel E1:** Organ weights. **Panel E2:** Body weight adjusted organ weights. LD = low-dose, HD = high-dose, *p<0.05.

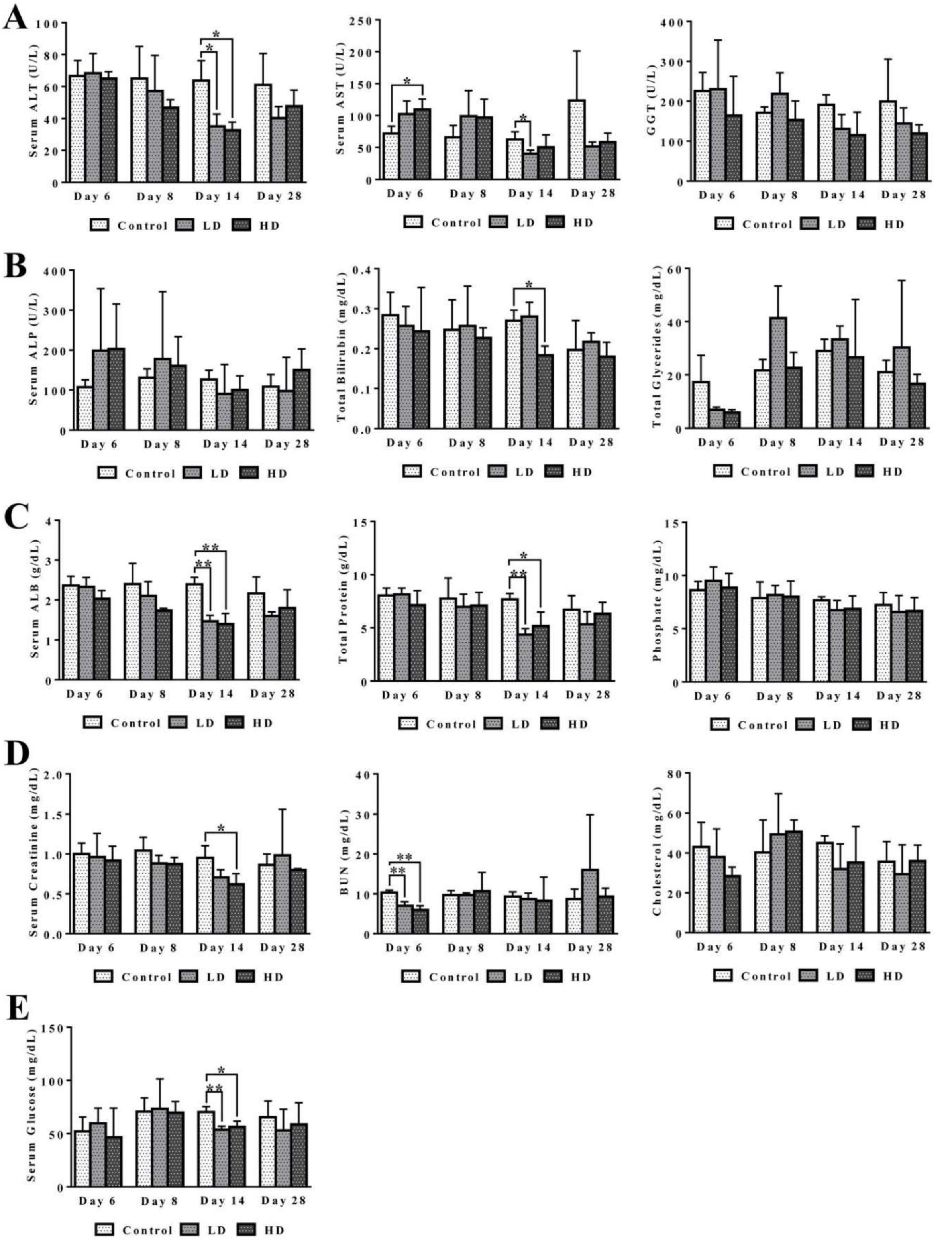

Supplementary Figure S2: Time resolved serum biochemistries after repeated diclofenac treatment for 28 days. Panel A1-A3: ALT, AST and γGT. **Panel B1-B3:** ALP, TBIL, TG. **Panel C1-C3:** ALB, total protein and phosphate. **Panel D1-D3:** CREA, BUN and cholesterol. **Panel E:** Serum glucose. LD = low-dose, HD = high-dose, *$p<0.05$, **$p<0.01$, ***$p<0.001$.

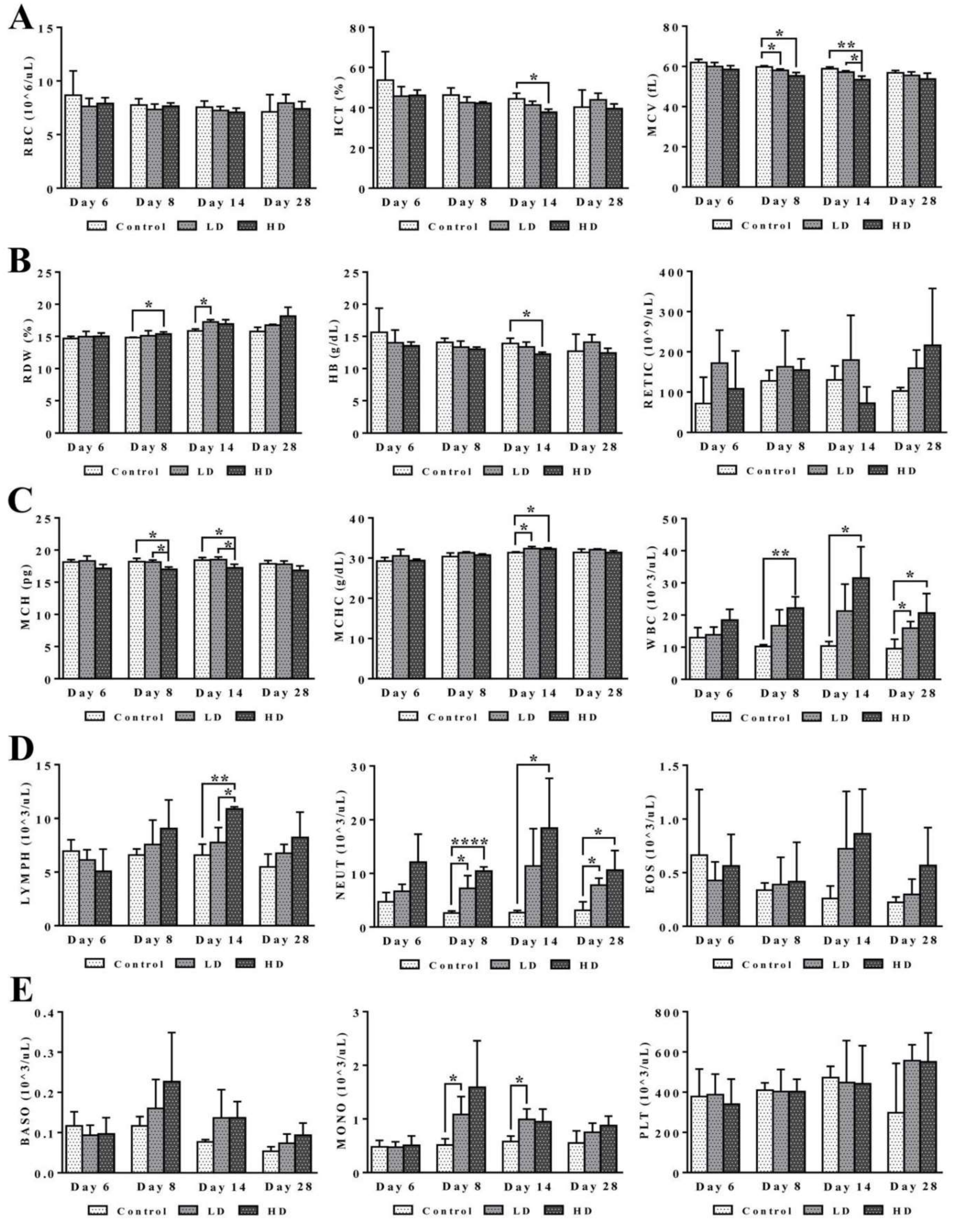

Supplementary Figure S3: Time resolved blood smears after repeated diclofenac treatment for 28 days. **Panel A1-A3:** RBC, HCT and MCV. **Panel B1-B3:** RDW, HB and RET. **Panel C1-C3:** MCH, MCHC and WBC. **Panel D1-D3:** Lymphocytes, neutrophils and

eosinophils **Panel E1-E3:** Basophils, monocytes and platelets. LD = low-dose, HD = high-dose, *p<0.05, **p<0.01, ***p<0.001, ****p<0.0001.

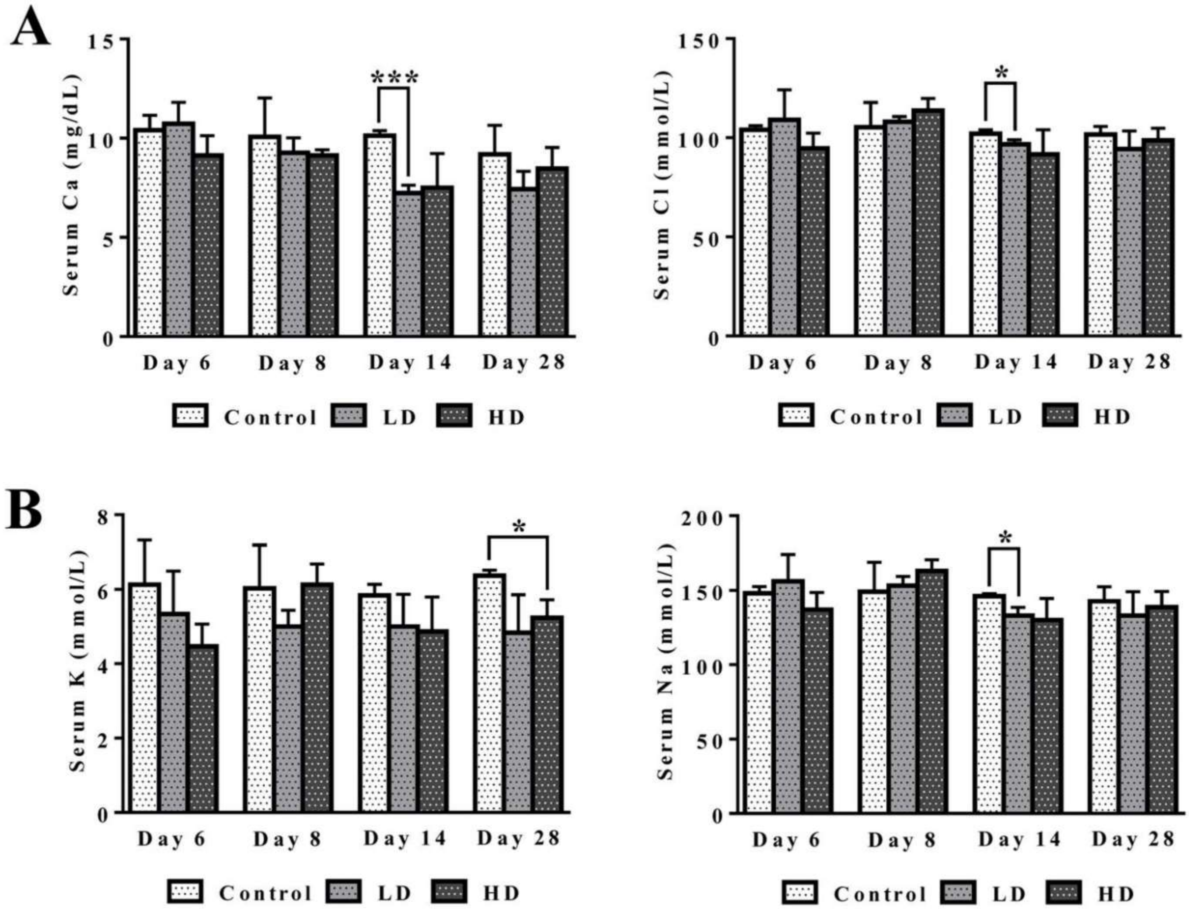

Supplementary Figure S4: **Time resolved serum electrolytes after repeated diclofenac treatment for 28 days**. **Panel A:** Serum calcium and chloride. **Panel B:** Serum potassium and sodium. LD = low-dose, HD = high-dose, *p<0.05, ***p<0.001.

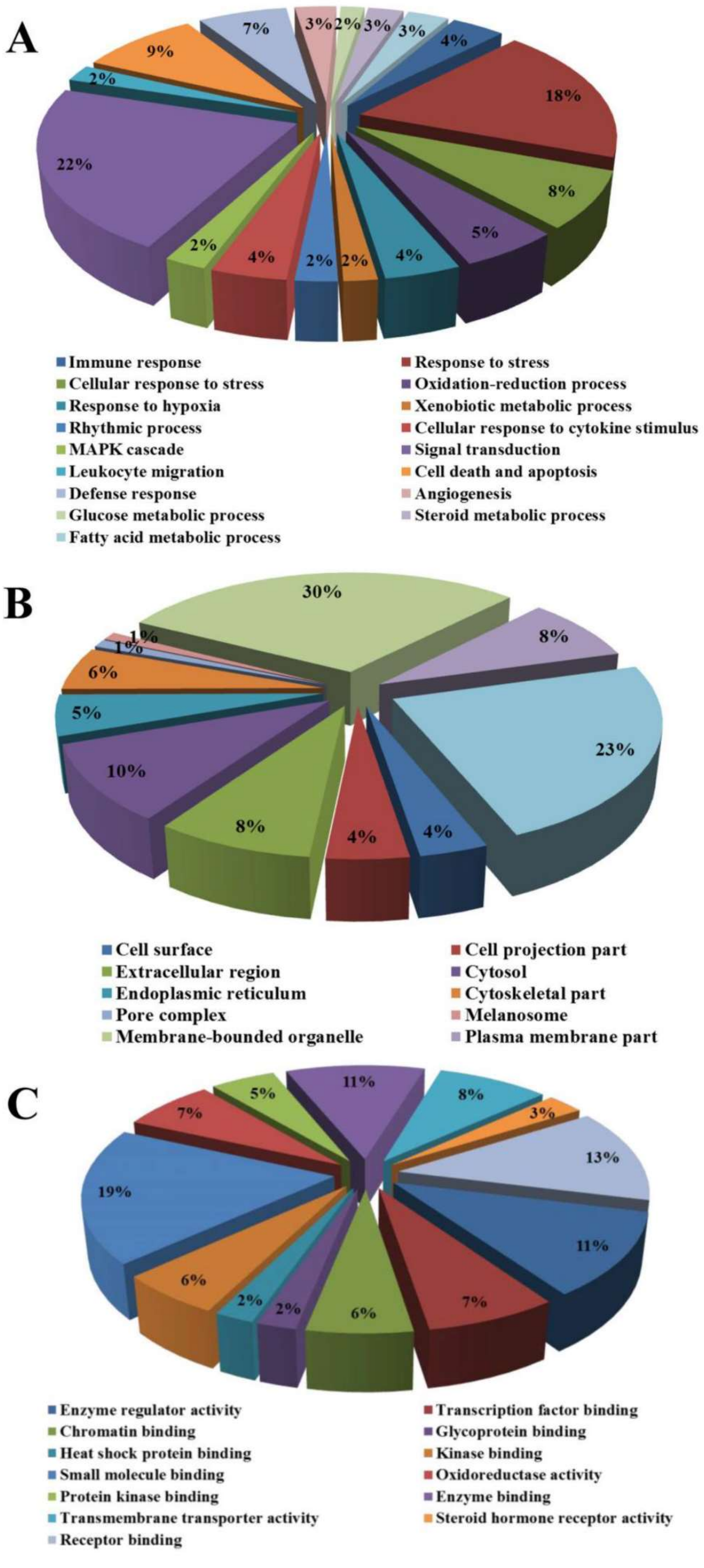
A
7%
3%
2%
3%
3%
4%
9%
2%
18%
22%
8%
5%
2%
4%
2%
2%
4%
Immune response
Response to stress
Cellular response to stress
Oxidation-reduction process
Response to hypoxia
Xenobiotic metabolic process
Rhythmic process
Cellular response to cytokine stimulus
MAPK cascade
Signal transduction
Leukocyte migration
Cell death and apoptosis
Defense response
Angiogenesis
Glucose metabolic process
Steroid metabolic process
Fatty acid metabolic process
B
30%
1%
1%
8%
6%
5%
23%
10%
8%
4%
4%
Cell surface
Cell projection part
Extracellular region
Cytosol
Endoplasmic reticulum
Cytoskeletal part
Pore complex
Melanosome
Membrane-bounded organelle
Plasma membrane part
C
11%
5%
8%
7%
3%
13%
19%
11%
6%
2%
2%
6%
7%
Enzyme regulator activity
Transcription factor binding
Chromatin binding
Glycoprotein binding
Heat shock protein binding
Kinase binding
Small molecule binding
Oxidoreductase activity
Protein kinase binding
Enzyme binding
Transmembrane transporter activity
Steroid hormone receptor activity
Receptor binding

Supplementary Figure S5: GO terms regulated in liver after low-dose diclofenac treatment. The gene ontology properties of DEGs were analyzed using the GeneXplain platform and a *p*-value threshold that was set to <0.05. The pie charts depict the distribution of A) key biological processes, B) cellular components and C) molecular functions of low-dose diclofenac treatment.

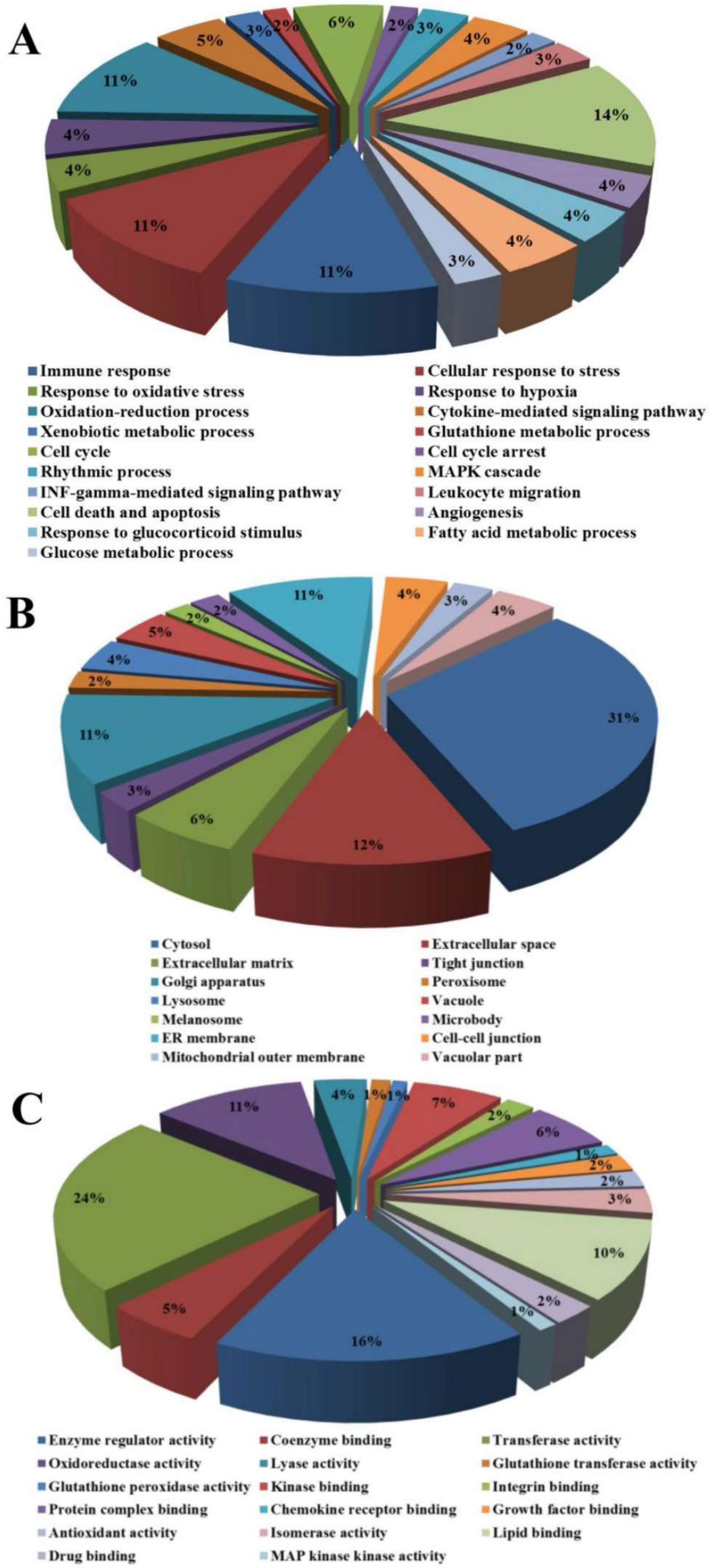
A
Immune response
Cellular response to stress
Response to oxidative stress
Response to hypoxia
Oxidation-reduction process
Cytokine-mediated signaling pathway
Xenobiotic metabolic process
Glutathione metabolic process
Cell cycle
Cell cycle arrest
Rhythmic process
MAPK cascade
INF-gamma-mediated signaling pathway
Leukocyte migration
Cell death and apoptosis
Angiogenesis
Response to glucocorticoid stimulus
Fatty acid metabolic process
Glucose metabolic process
B
Cytosol
Extracellular space
Extracellular matrix
Tight junction
Golgi apparatus
Peroxisome
Lysosome
Vacuole
Melanosome
Microbody
ER membrane
Cell-cell junction
Mitochondrial outer membrane
Vacuolar part
C
Enzyme regulator activity
Coenzyme binding
Transferase activity
Oxidoreductase activity
Lyase activity
Glutathione transferase activity
Glutathione peroxidase activity
Kinase binding
Integrin binding
Protein complex binding
Chemokine receptor binding
Growth factor binding
Antioxidant activity
Isomerase activity
Lipid binding
Drug binding
MAP kinase kinase activity

Supplementary Figure S6: GO terms regulated in liver after high-dose diclofenac treatment. The gene ontology properties of DEGs were analyzed using GeneXplain platform and the *p*-value threshold was set as <0.05. The pie charts depict the distribution of A) key biological processes, B) cellular components and C) molecular functions of high-dose diclofenac treatment.

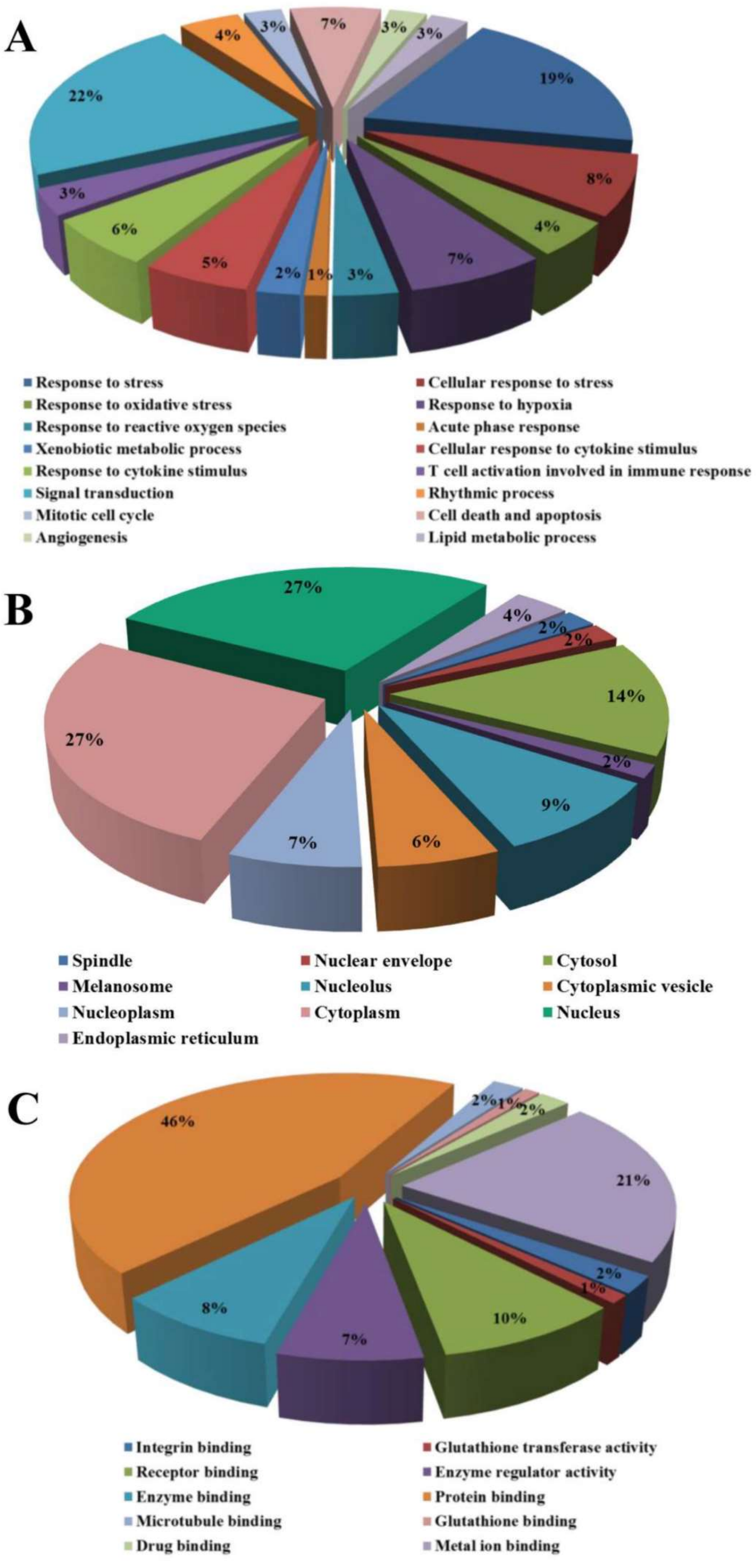
A
22%
4%
3%
7%
3%
3%
19%
8%
4%
7%
3%
1%
2%
5%
6%
3%
Response to stress
Cellular response to stress
Response to oxidative stress
Response to hypoxia
Response to reactive oxygen species
Acute phase response
Xenobiotic metabolic process
Cellular response to cytokine stimulus
Response to cytokine stimulus
T cell activation involved in immune response
Signal transduction
Rhythmic process
Mitotic cell cycle
Cell death and apoptosis
Angiogenesis
Lipid metabolic process
B
27%
4%
2%
2%
14%
27%
2%
9%
7%
6%
Spindle
Nuclear envelope
Cytosol
Melanosome
Nucleolus
Cytoplasmic vesicle
Nucleoplasm
Cytoplasm
Nucleus
Endoplasmic reticulum
C
46%
2%
1%
2%
21%
2%
1%
8%
7%
10%
Integrin binding
Glutathione transferase activity
Receptor binding
Enzyme regulator activity
Enzyme binding
Protein binding
Microtubule binding
Glutathione binding
Drug binding
Metal ion binding

Supplementary Figure S7: GO terms regulated in kidney after low-dose diclofenac treatment. The gene ontology properties of DEGs were analyzed using the GeneXplain platform and a *p*-value threshold that was set to <0.05. The pie charts depict the distribution of significant A) biological processes, B) cellular components and C) molecular functions of low-dose diclofenac treatment.

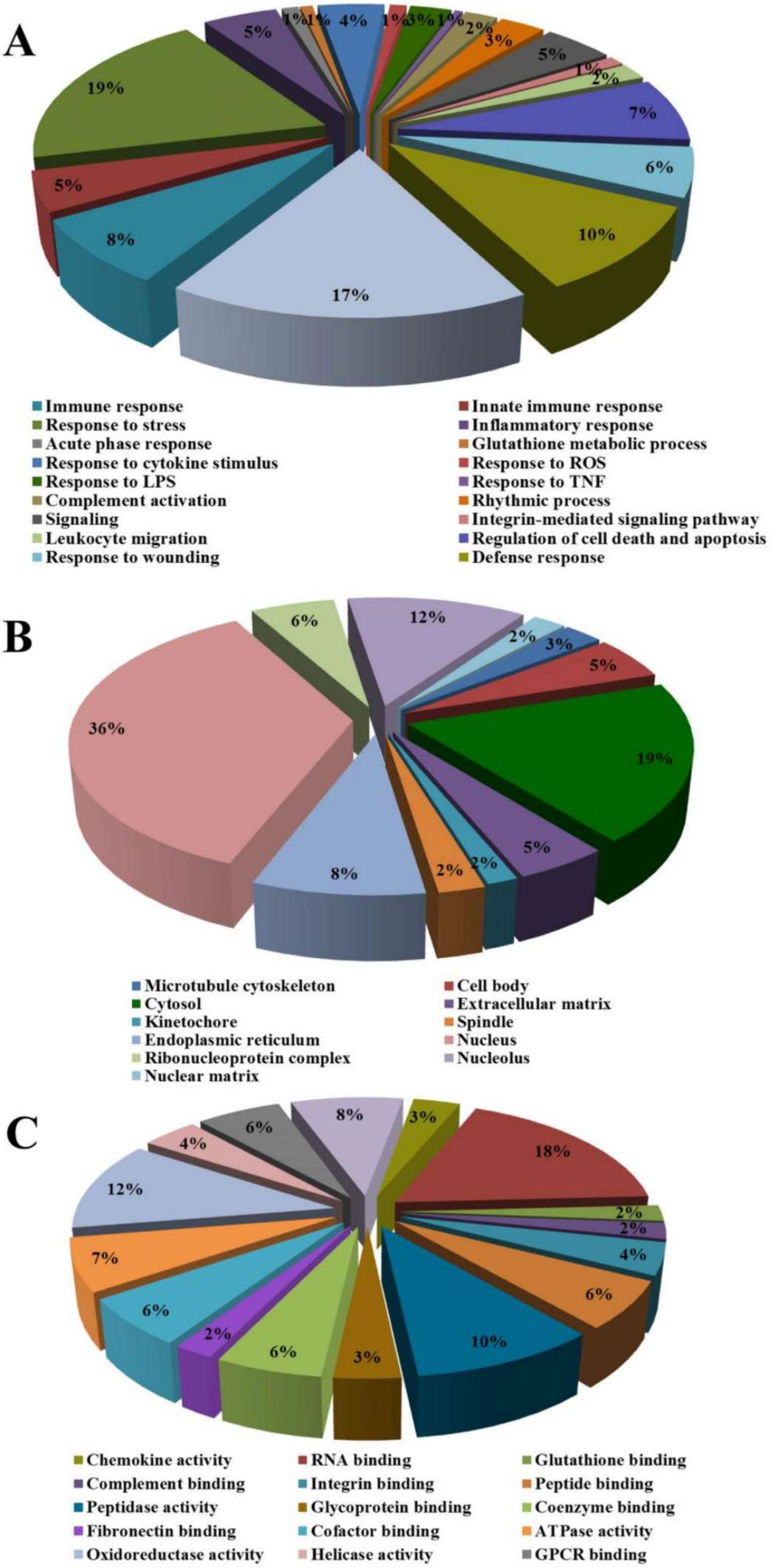
A
1%
1%
4%
1%
3%
1%
2%
3%
5%
5%
1%
2%
19%
7%
6%
5%
8%
10%
17%
Immune response
Innate immune response
Response to stress
Inflammatory response
Acute phase response
Glutathione metabolic process
Response to cytokine stimulus
Response to ROS
Response to LPS
Response to TNF
Complement activation
Rhythmic process
Signaling
Integrin-mediated signaling pathway
Leukocyte migration
Regulation of cell death and apoptosis
Response to wounding
Defense response
B
6%
12%
2%
3%
5%
36%
19%
5%
8%
2%
2%
Microtubule cytoskeleton
Cell body
Cytosol
Extracellular matrix
Kinetochore
Spindle
Endoplasmic reticulum
Nucleus
Ribonucleoprotein complex
Nucleolus
Nuclear matrix
C
8%
3%
6%
4%
18%
12%
2%
2%
7%
4%
6%
6%
2%
6%
3%
10%
Chemokine activity
RNA binding
Glutathione binding
Complement binding
Integrin binding
Peptide binding
Peptidase activity
Glycoprotein binding
Coenzyme binding
Fibronectin binding
Cofactor binding
ATPase activity
Oxidoreductase activity
Helicase activity
GPCR binding
Protein complex binding

Supplementary Figure S8: GO terms regulated in kidney after high-dose diclofenac treatment. The gene ontology properties of DEGs were analyzed using the GeneXplain platform and the *p*-value threshold was set to <0.05. The pie charts depict the distribution of significant A) biological processes, B) cellular components and C) molecular functions of high-dose diclofenac treatment.

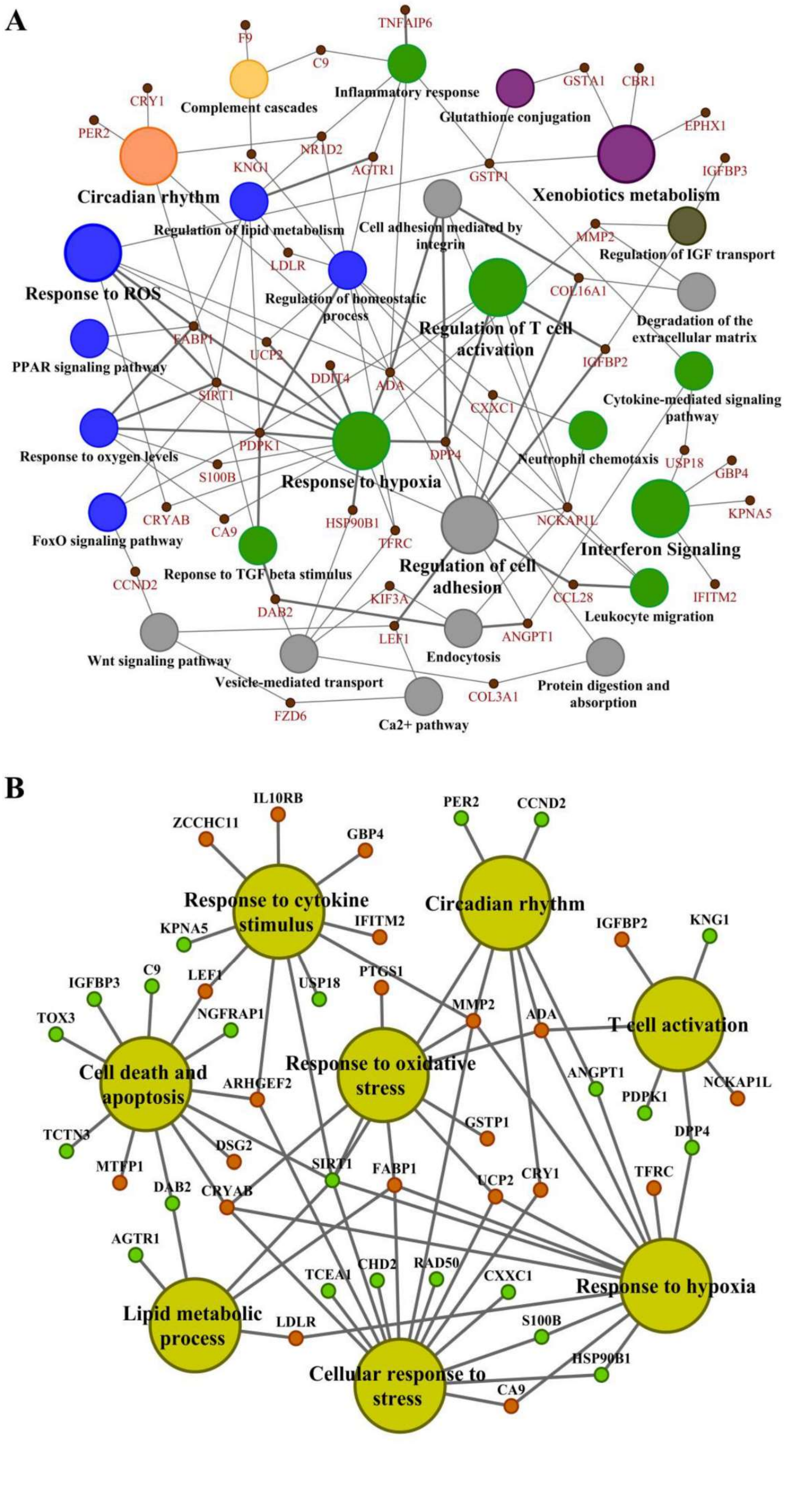
A
TNFAIP6
F9
C9
Inflammatory response
Complement cascades
CRY1
GSTA1
CBR1
Glutathione conjugation
PER2
EPHX1
NR1D2
KNG1
AGTR1
GSTP1
IGFBP3
Circadian rhythm
Xenobiotics metabolism
Regulation of lipid metabolism
Cell adhesion mediated by integrin
MMP2
Regulation of IGF transport
LDLR
Response to ROS
COL16A1
Regulation of homeostatic process
Degradation of the extracellular matrix
Regulation of T cell activation
FABP1
PPAR signaling pathway
UCP2
IGFBP2
DDIT4
ADA
Cytokine-mediated signaling pathway
SIRT1
CXXC1
PDPK1
DPP4
Response to oxygen levels
Neutrophil chemotaxis
USP18
S100B
Response to hypoxia
GBP4
CRYAB
HSP90B1
NCKAP1L
KPNA5
CA9
FoxO signaling pathway
TFRC
Interferon Signaling
Reponse to TGF beta stimulus
Regulation of cell adhesion
CCND2
KIF3A
CCL28
IFITM2
DAB2
Leukocyte migration
ANGPT1
LEF1
Endocytosis
Wnt signaling pathway
Protein digestion and absorption
Vesicle-mediated transport
COL3A1
FZD6
Ca2+ pathway
B
IL10RB
ZCCHC11
GBP4
PER2
CCND2
Response to cytokine stimulus
Circadian rhythm
KPNA5
IFITM2
IGFBP2
KNG1
IGFBP3
C9
LEF1
PTGS1
USP18
TOX3
NGFRAP1
MMP2
ADA
T cell activation
Cell death and apoptosis
Response to oxidative stress
ARHGEF2
ANGPT1
NCKAP1L
PDPK1
TCTN3
GSTP1
DPP4
DSG2
MTFP1
SIRT1
FABP1
UCP2
CRY1
TFRC
DAB2
CRYAB
AGTR1
TCEA1
CHD2
RAD50
CXXC1
Response to hypoxia
Lipid metabolic process
LDLR
S100B
HSP90B1
Cellular response to stress
CA9

Supplementary Figure S9: **Gene ontology and pathway mapping network of DEGs in kidney in response to low-dose diclofenac treatment**. A visualization of kidney enriched pathway terms and biological processes computed with the ClueGO and the GeneXplain software of low-dose treated animals. **Panel A:** Network constructed by the plug-in ClueGO of the Cytoscape software. **Panel B:** The enriched biological processes and pathways were annotated by the GeneXplain platform and visualized using the Cytoscape software version 3.4. The red and green colored nodes illustrate up- and down regulated genes, respectively.

A

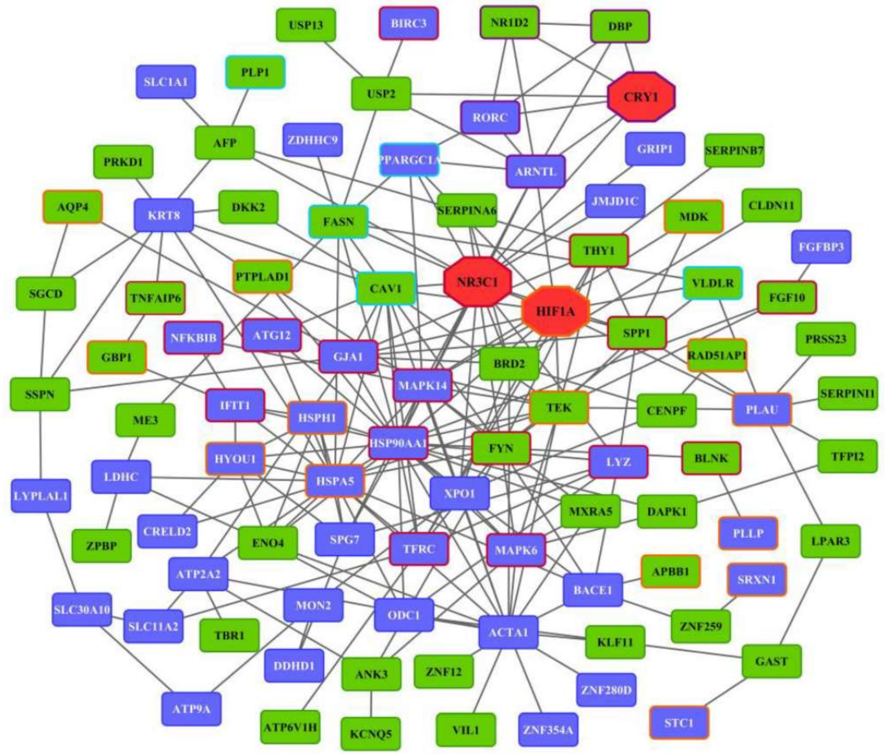
USP13
BIRC3
NR1D2
DBP
SLC1A1
PLP1
USP2
CRY1
RORC
AFP
ZDHHC9
PPARGC1A
ARNTL
GRIP1
SERPINB7
PRKD1
JMJD1C
AQP4
KRT8
DKK2
FASN
SERPINA6
MDK
CLDN11
THY1
FGFBP3
SGCD
TNFAIP6
PTPLAD1
CAV1
NR3C1
HIF1A
VLDLR
FGF10
NFKBIB
ATG12
SPP1
PRSS23
GBP1
GJA1
RAD51AP1
SSPN
MAPK14
BRD2
SERPINI1
ME3
IFIT1
HSPH1
TEK
CENPF
PLAU
HSP90AA1
FYN
TFPI2
HYOU1
LYZ
BLNK
LYPLAL1
LDHC
HSPA5
XPO1
MXRA5
DAPK1
ZPBP
CRELD2
ENO4
SPG7
TFRC
MAPK6
PLLP
LPAR3
ATP2A2
APBB1
SRXN1
BACE1
SLC30A10
SLC11A2
MON2
ODC1
ACTA1
ZNF259
TBR1
KLF11
GAST
DDHD1
ANK3
ZNF12
ZNF280D
ATP9A
ATP6V1H
KCNQ5
VIL1
ZNF354A
STC1

B

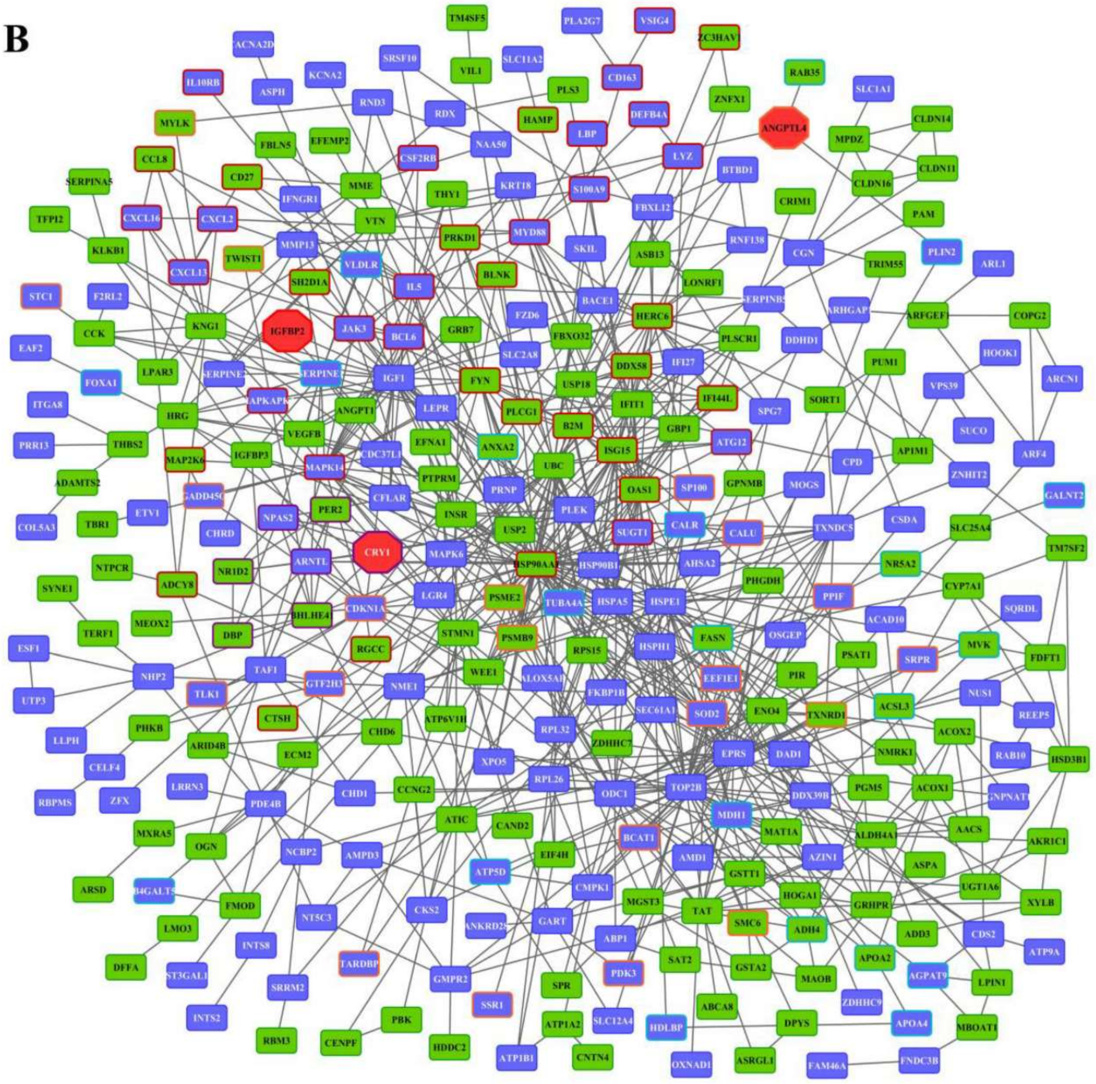

Supplementary Figure S10: Gene/protein interaction networks in the liver of diclofenac treated animals. **Panel A:** Low-dose treatment. A total of 153 DEGs were used to construct a PPI network of which 92 interacted among 217 PPIs. **Panel B:** High-dose treatment. A total of 488 DEGs were used to construct a PPI network of which 325 engaged in 885 PPIs. The purple and green color highlights up- and down- regulated DEGs. The red hexagon denotes master regulator molecules of the network. Immune and inflammation response genes are marked by red colored boxes, whereas circadian rhythm, stress response genes and lipid metabolic genes are highlighted in maroon, orange and blue colored boxes, respectively. The protein interaction networks were constructed using STRING version 10.5. Zoom in for better readability of individual genes.

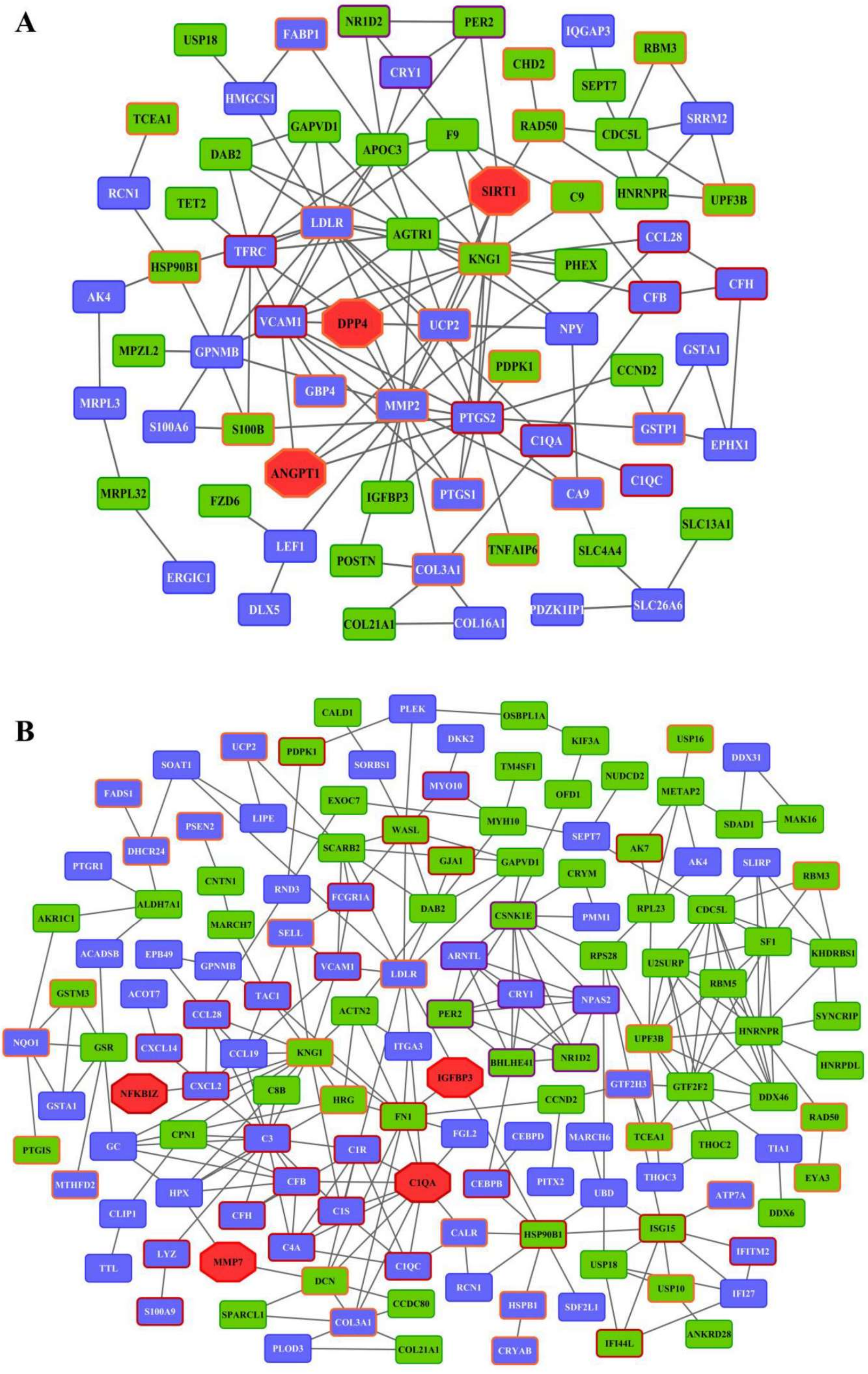
A
USP18
FABP1
NR1D2
PER2
IQGAP3
RBM3
CRY1
CHD2
SEPT7
HMGCS1
TCEA1
GAPVD1
F9
RAD50
CDC5L
SRRM2
DAB2
APOC3
SIRT1
C9
HNRNPR
UPF3B
RCN1
TET2
LDLR
AGTR1
CCL28
TFRC
KNG1
PHEX
HSP90B1
CFH
AK4
CFB
VCAM1
DPP4
UCP2
NPY
MPZL2
GPNMB
GSTA1
PDPK1
CCND2
GBP4
MRPL3
MMP2
PTGS2
S100A6
S100B
GSTP1
C1QA
EPHX1
ANGPT1
C1QC
MRPL32
CA9
FZD6
IGFBP3
PTGS1
SLC13A1
LEF1
TNFAIP6
SLC4A4
POSTN
COL3A1
ERGIC1
DLX5
PDZK1IP1
SLC26A6
COL21A1
COL16A1
B
CALD1
PLEK
OSBPL1A
DKK2
KIF3A
USP16
UCP2
PDPK1
SORBS1
DDX31
SOAT1
TM4SF1
NUDCD2
MYO10
OFD1
METAP2
FADS1
LIPE
EXOC7
PSEN2
MYH10
SDAD1
MAK16
WASL
SEPT7
DHCR24
SCARB2
AK7
PTGR1
GJA1
GAPVD1
AK4
SLIRP
RBM3
CNTN1
CRYM
RND3
FCGR1A
ALDH7A1
AKR1C1
DAB2
RPL23
CDC5L
MARCH7
CSNK1E
PMM1
SELL
SF1
ACADSB
EPB49
ARNTL
RPS28
KHDRBS1
GPNMB
VCAM1
LDLR
U2SURP
RBM5
GSTM3
ACOT7
TAC1
CRY1
NPAS2
CCL28
ACTN2
PER2
SYNCRIP
NQO1
GSR
CXCL14
UPF3B
HNRNPR
ITGA3
KNG1
CCL19
NR1D2
BHLHE41
IGFBP3
HNRNPDL
NFKBIZ
CXCL2
GTF2H3
GTF2F2
C8B
HRG
CCND2
DDX46
GSTA1
FN1
RAD50
CPN1
GC
C3
C1R
FGL2
CEBPD
MARCH6
TCEA1
THOC2
TIA1
PTGIS
C1QA
MTHFD2
HPX
CFB
CEBPB
PITX2
THOC3
EYA3
CLIP1
CFH
C1S
UBD
ATP7A
ISG15
CALR
HSP90B1
DDX6
C4A
LYZ
MMP7
IFITM2
TTL
C1QC
USP18
DCN
IFI27
RCN1
USP10
CCDC80
S100A9
HSPB1
SDF2L1
SPARCL1
ANKRD28
COL3A1
PLOD3
COL21A1
CRYAB
IFI44L

Supplementary Figure S11: Gene/protein interaction networks in the kidney of diclofenac treated animals. **Panel A:** Low-dose treatment. Out of 144 DEGs a network that consists of 76 DEGs was constructed and interacted among 105 PPIs. **Panel B:** High-dose treatment. Out of 286 DEGs a network that consists of 180 DEGs was constructed and interacted among 412 PPIs The purple and green color highlights up- and down- regulated DEGs. The red hexagon denotes master regulator molecules of the network. Immune and inflammation response genes were marked by red colored boxes, whereas circadian rhythm, stress response genes and lipid metabolic genes are highlighted in maroon, orange and blue colored boxes, respectively. The protein interaction networks were constructed using STRING version 10.5. Zoom in for better readability of individual genes.

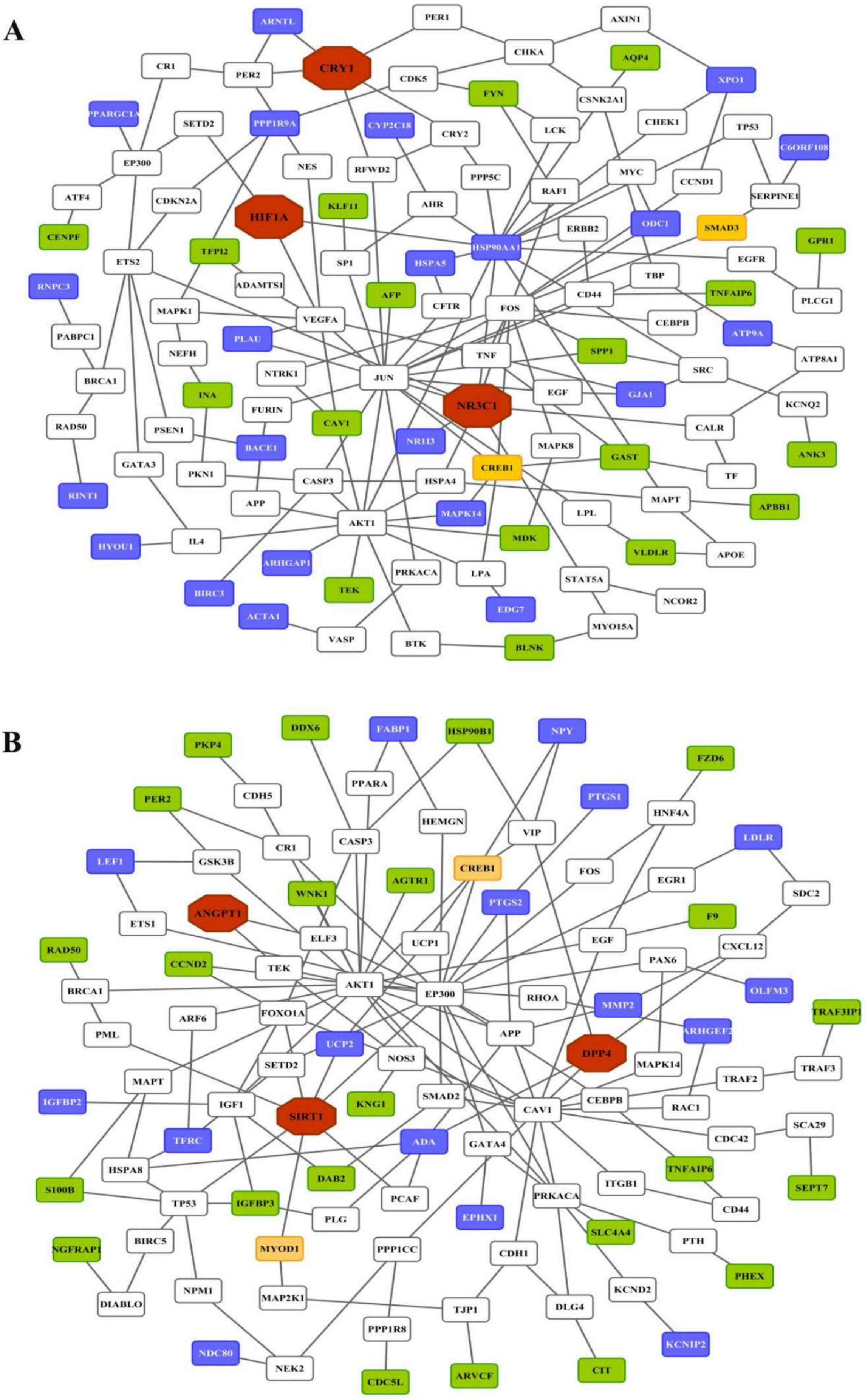
A
PER1
AXIN1
ARNTL
CRY1
CHKA
AQP4
CR1
PER2
CDK5
XPO1
FYN
CSNK2A1
PPARGC1A
SETD2
PPP1R9A
CYP2C18
CRY2
LCK
CHEK1
TP53
C6ORF108
EP300
NES
RFWD2
PPP5C
MYC
CCND1
ATF4
CDKN2A
AHR
RAF1
SERPINE1
HIF1A
KLF11
CENPF
ERBB2
ODC1
SMAD3
GPR1
TFPI2
HSP90AA1
ETS2
SP1
HSPA5
EGFR
RNPC3
ADAMTS1
AFP
TBP
TNFAIP6
PLCG1
MAPK1
VEGFA
CFTR
FOS
CD44
CEBPB
ATP9A
PABPC1
PLAU
SPP1
NEFH
TNF
ATP8A1
NTRK1
JUN
SRC
BRCA1
INA
EGF
GJA1
NR3C1
KCNQ2
FURIN
CAV1
RAD50
PSEN1
CALR
NR1I3
MAPK8
ANK3
BACE1
GATA3
GAST
CREB1
PKN1
CASP3
HSPA4
TF
RINT1
MAPT
APP
APBB1
MAPK14
LPL
AKT1
MDK
IL4
HYOU1
VLDLR
APOE
ARHGAP1
PRKACA
LPA
STAT5A
BIRC3
TEK
NCOR2
EDG7
ACTA1
MYO15A
VASP
BTK
BLNK
B
DDX6
FABP1
HSP90B1
NPY
PKP4
FZD6
PPARA
CDH5
PER2
PTGS1
HEMGN
HNF4A
CASP3
VIP
CR1
LDLR
LEF1
GSK3B
CREB1
FOS
EGR1
AGTR1
SDC2
WNK1
PTGS2
ANGPT1
ETS1
F9
ELF3
UCP1
EGF
CXCL12
RAD50
PAX6
CCND2
TEK
AKT1
OLFM3
BRCA1
EP300
RHOA
MMP2
TRAF3IP1
ARF6
FOXO1A
PML
APP
ARHGEF2
UCP2
DPP4
NOS3
SETD2
MAPK14
TRAF3
MAPT
TRAF2
SMAD2
CEBPB
IGFBP2
IGF1
KNG1
CAV1
RAC1
SIRT1
SCA29
TFRC
CDC42
ADA
GATA4
HSPA8
TNFAIP6
ITGB1
SEPT7
DAB2
S100B
PCAF
PRKACA
TP53
IGFBP3
CD44
PLG
EPHX1
SLC4A4
PTH
NGFRAP1
BIRC5
MYOD1
PPP1CC
CDH1
PHEX
KCND2
NPM1
MAP2K1
DIABLO
TJP1
DLG4
PPP1R8
KCNIP2
NDC80
NEK2
CIT
ARVCF
CDC5L

Supplementary Figure S12: Master regulatory gene networks in liver and kidney of low-dose diclofenac treated animals. Based on interaction information available in the GeneWays database the master regulatory gene networks were constructed and fused using the GeneXplain platform. The red, violet, green, white and yellow colored nodes represent the genes coding for master regulators, up-, down regulated DEGs, connecting genes and enriched transcription factors, respectively. **Panel A**: Master regulatory network in liver of low-dose diclofenac treatment: The network comprised 114 genes of which 43 were significantly regulated. **Panel B**: Master regulatory network in kidney of low-dose diclofenac treatment: The network comprised 103 genes of which 43 genes were significantly regulated.

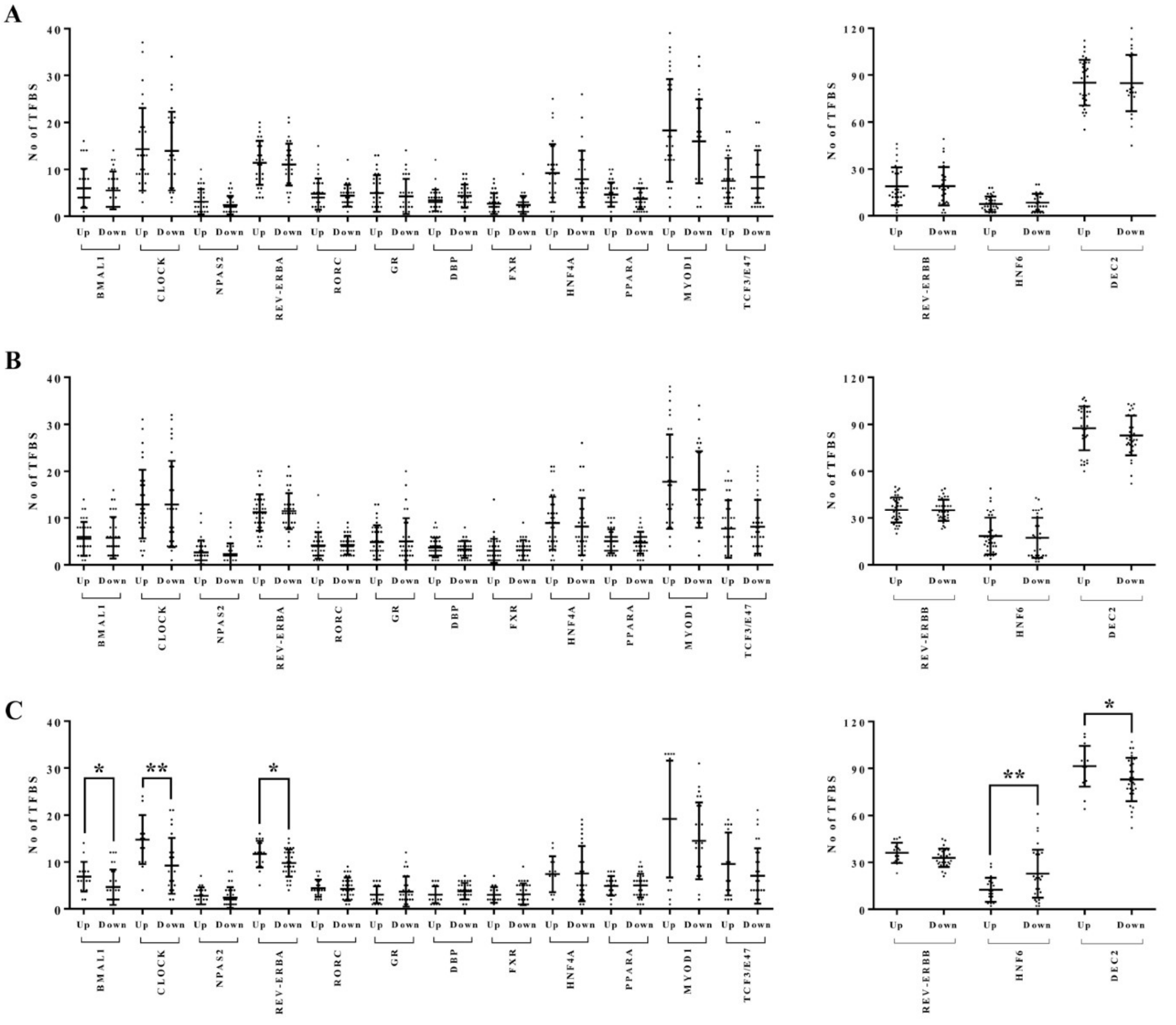

Supplementary Figure S13: **Enriched transcription factor binding sites for liver clock regulated genes**. Shown are TFBS in promoters of significantly regulated genes of the core

circadian transcription factors BMAL1, CLOCK, NPAS2, REV-ERBA, REV-ERBß, RORC in addition to GR, DBP, FXR, HNF4A, HNF6, PPARA and DEC2 which participate in liver clock oscillations. **Panel A**: TFBS in promoters of DEGs coding for immune and inflammation. **Panel B**: TFBS in promoters of DEGs coding for stress response genes. **Panel C**: TFBS in promoters of DEGs coding for metabolic processes. *$p<0.05$, **$p<0.01$.

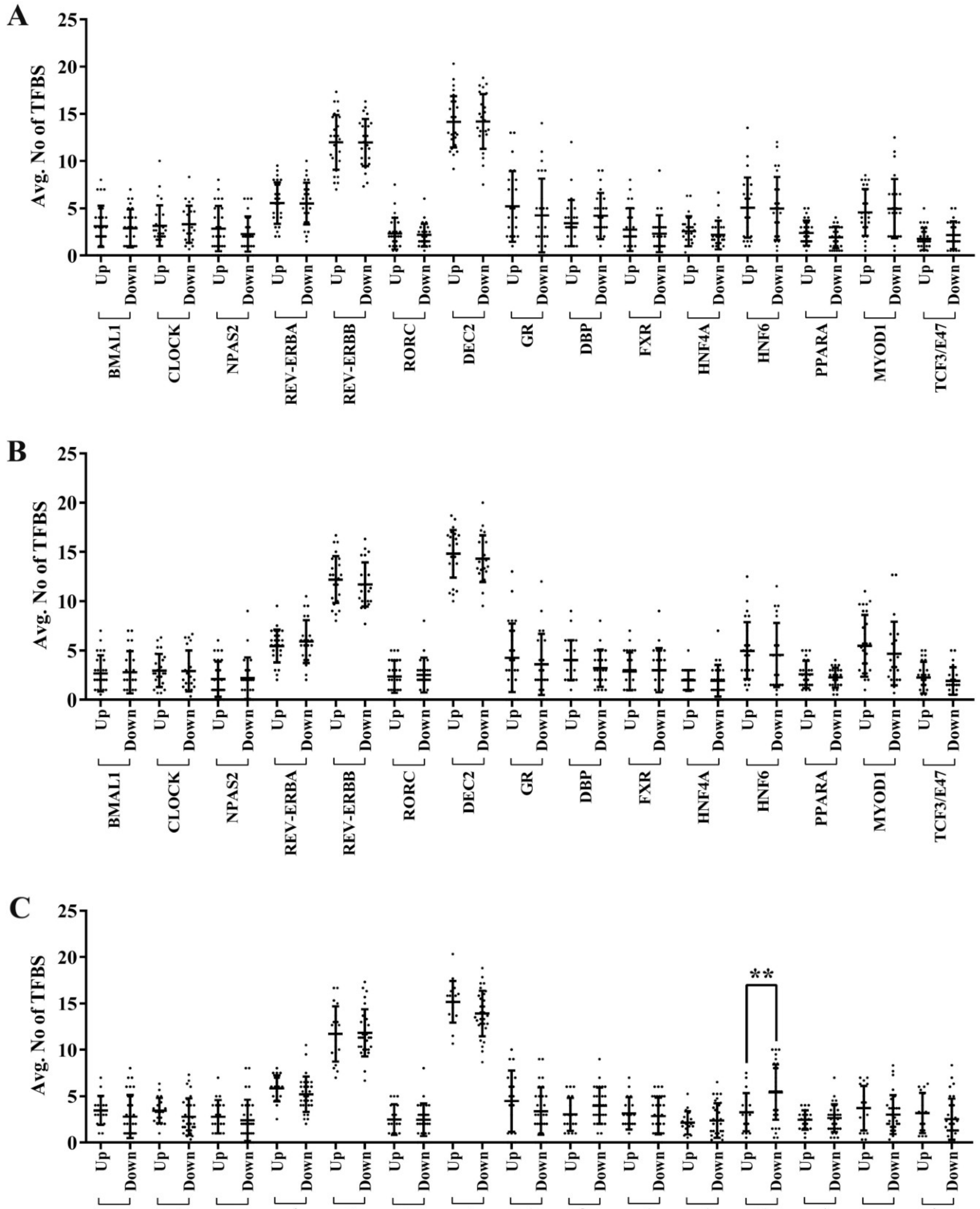

Supplementary Figure S14: **Enriched TFBS in association with liver clock genes**. The enriched TFBS at gene specific promoters of significantly regulated DEGs coding for immune, stress, inflammation and metabolism with the length of -1000 to +100 base pairs relative to TSS were interrogated using the GeneXplain software version 6.0. Each dot illustrates an average

number of TFBS at the promoter region of the genes. **Panel A:** Average number of TFBS for immune and inflammation coding genes. **Panel B:** Average number of TFBS at promoters of stress response genes. **Panel C:** Average number of TFBS at promoters of DEGs coding for metabolic processes. **p<0.01.

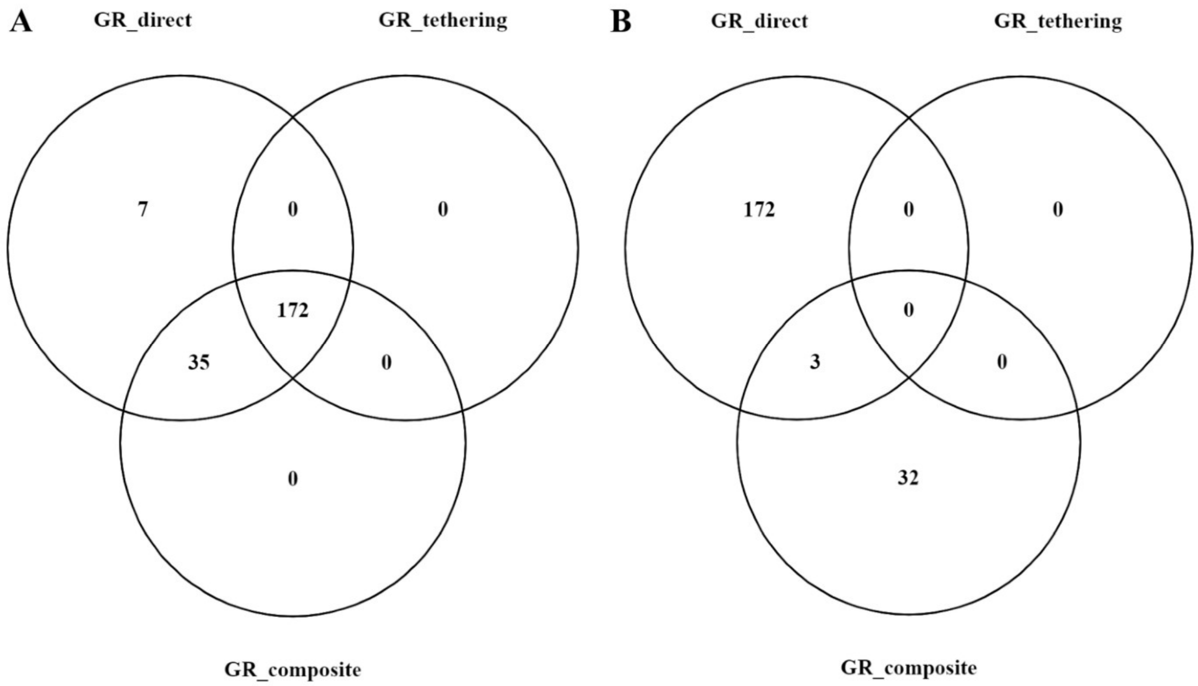

Supplementary Figure S15: **Comparison of proposed GR signaling models in hepatic DEGs**. The direct, tethering or composite models of GR activity was proposed by Oakley and Cidlowski [50] that results in either activated or repressed transcriptional responses. The proposed models were interrogated at gene specific promoters of hepatic DEGs using the GeneXplain software version 6.0. **Panel A**: Venn diagram illustrates the number of up-regulated genes associated with direct, tethering or composite model of glucocorticoid receptor activity. **Panel B**: Venn diagram illustrates the number of down-regulated genes associated with glucocorticoid receptor activity.

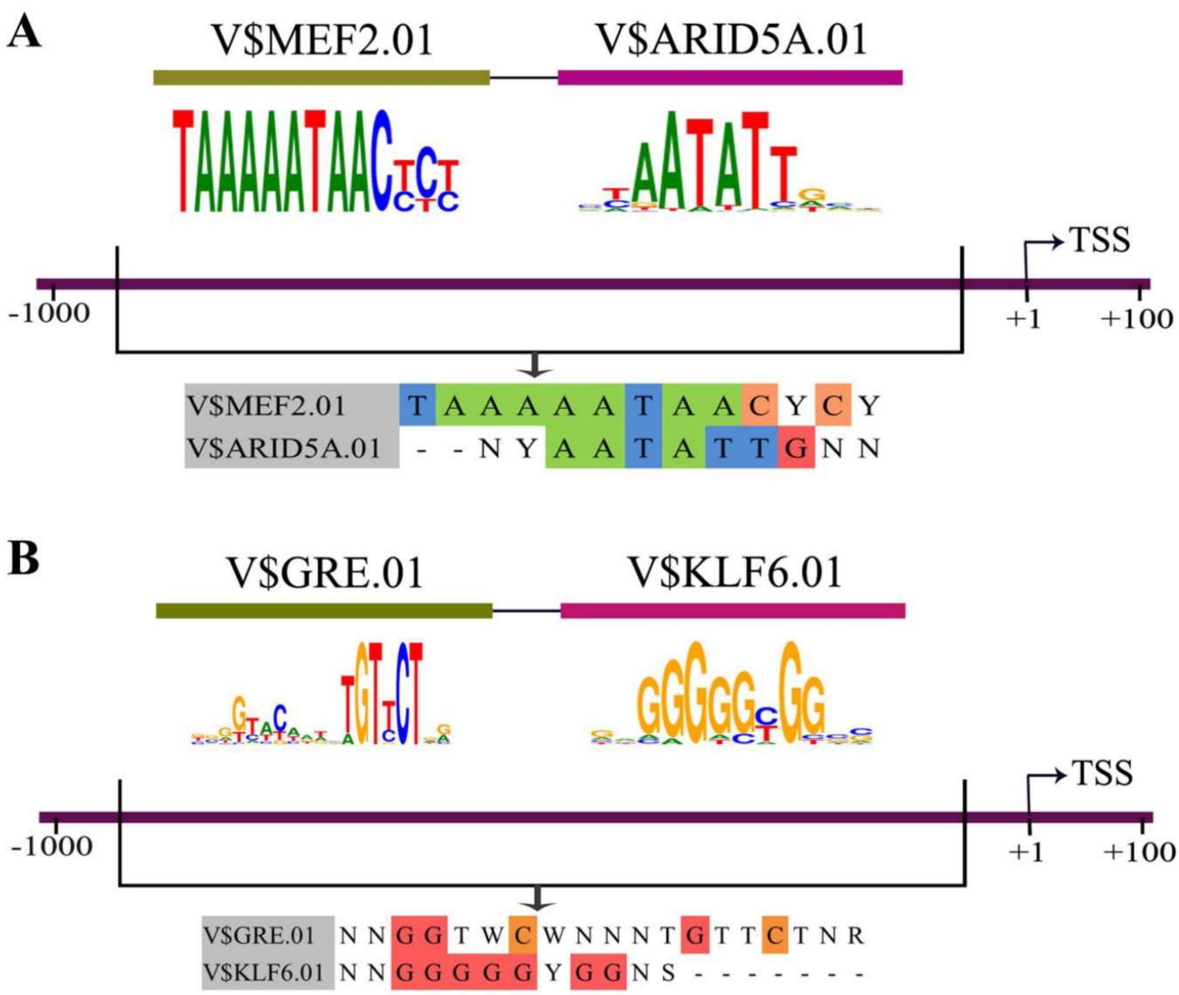

Supplementary Figure S16: Composite modules search to predict gene regulation in liver in response to diclofenac treatment. DEGs coding for immune, stress, inflammation, hypoxia, cytokine and acute-phase responses were interrogated using the Genomatix FrameWorker tool. Transcription factor binding sites at gene specific promoters with the length of -1000 to +100 base pairs relative to TSS were analyzed. The sequence alignment defines the overlapping regions of consensus sequences specific for TFBS. **Panel A**: Composite modules of co-bound transcription factors at promoters of low-dose diclofenac induced DEGs. **Panel B**: Composite module of regulated DEGs after high-dose diclofenac treatment in liver.

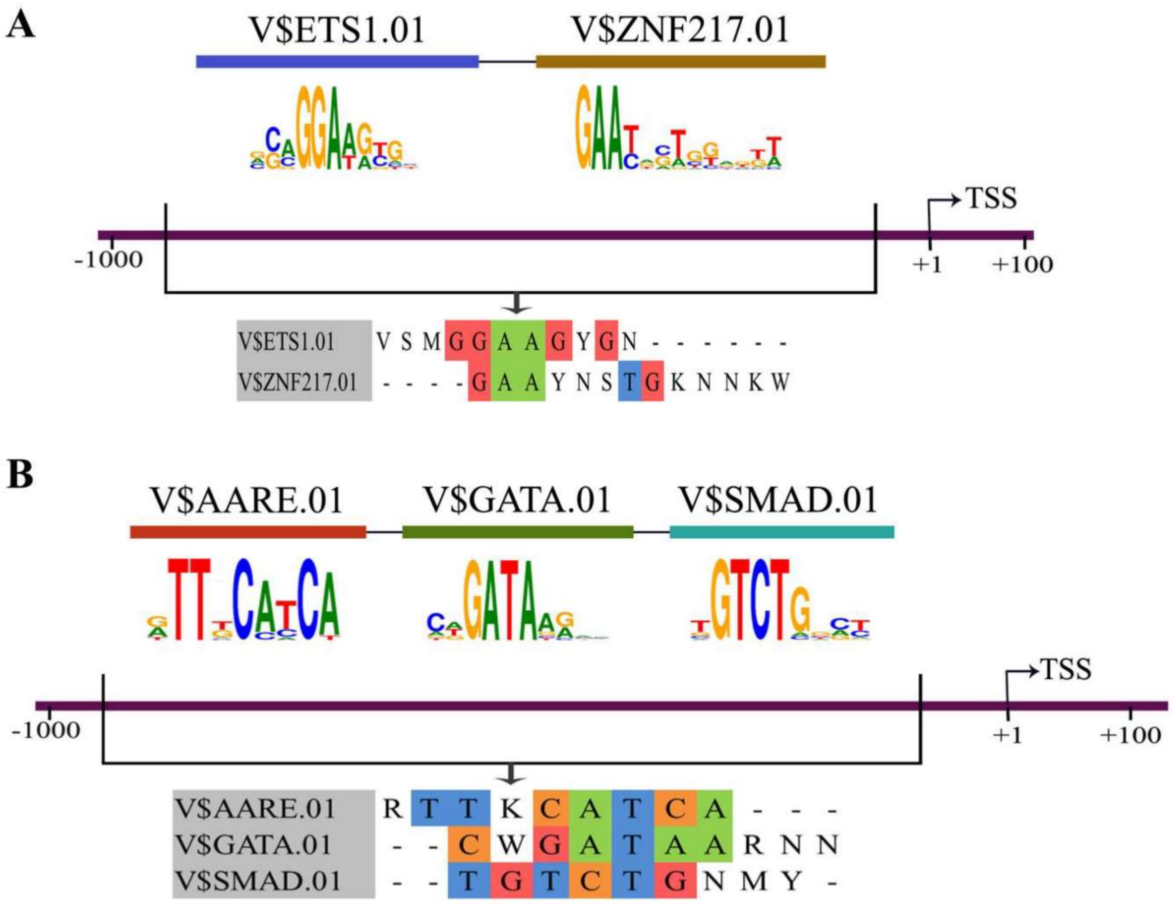

Supplementary Figure S17: Composite modules search to predict gene regulation in kidney in response to diclofenac treatment. Cis-regulatory modules at gene specific promoters of DEGs with the length of -1000 to +100 base pairs relative to TSS were interrogated using the Genomatix FrameWorker tool. The sequence alignment defines the overlapping regions of consensus sequences specific for TFBS. **Panel A**: Composite module of low-dose regulated DEGs after diclofenac treatment. **Panel B**: Composite module of high-dose regulated DEGs after diclofenac treatment.

Supplementary Table S1: Drug metabolism genes and solute carriers regulated in liver and kidney after diclofenac treatment.

Supplementary Table S2: Enriched biological processes in kidney. The functional properties of DEGs were analyzed using the GeneXplain platform and the *p*-value <0.05 considered as significant.

Supplementary Table S3: Over-represented transcription factor binding sites in liver and kidney DEGs after low- and high-dose diclofenac treatment.

Supplementary Table S4: Composite modules of enriched liver clock and glucocorticoid receptor (GR) regulated genes in response to diclofenac treatment.

Co-occupancy of circadian transcriptional regulators and glucocorticoid receptor was evaluated at the gene specific promoters of hepatic DEGs with the length of -1000 to +100 base pairs relative to TSS. The default filtering criteria of the GeneXplain platform were applied to determine statistically significant composite modules.

Supplementary Table S5: Glucocorticoid receptor binding in hepatic DEGs.

The direct, tethering or composite models of GR activity was proposed by Oakley and Cidlowski (PMID: 24084075) that results in either activated or repressed transcriptional responses. The proposed models were interrogated at gene specific promoters of hepatic DEGs using the GeneXplain software.

Supplementary Table S6: Primer sequences used in real-time RT-PCR analyses.
