# Supplementary material for: Diclofenac Disrupts the Circadian Clock and through Complex Cross-Talks Aggravates Immune-Mediated Liver Injury—A Repeated Dose Study in Minipigs for 28 Days
Source: Int J Mol Sci. 2023 Jan 11;24(2):1445. doi: 10.3390/ijms24021445 (PMC9863319; doi:10.3390/ijms24021445)
Supplement: Supplementary file 1 [file ijms-24-01445-s001.zip › Supplementary Table S1.pdf]

Supplementary Table S1: Drug metabolism genes and solute carriers regulated in liver and kidney after diclofenac treatment.

Probe ID	Gene symbol	Description	Fold change (average)±SD
**Low-dose_liver**			
**Phase I**			
Ssc.208.1.S1_at	CYP1A1	Cytochrome P450 family 1 subfamily A member 1	4.86±3.71
Ssc.203.1.S1_s_at	CYP3A22	Cytochrome P450 3A22	-6.5±4.01
Ssc.204.1.S1_at	CYP3A29	Cytochrome P450 3A29	-6.43±0.75
Ssc.206.1.S1_at	CYP2C32	Cytochrome P450 2C32	-1.88±1.14
Ssc.203.1.S1_s_at	CYP3A39	Cytochrome P450 3A39	-6.52±2.58
Ssc.30334.1.A1_at	CYP3A46	Cytochrome P450 3A46	-1.95±1.42
Ssc.2224.1.S1_at	CYP2C19	Cytochrome P450 family 2 subfamily C member 19	-1.59±0.2
**Phase III**			
Ssc.3451.1.S1_at	SLC11A2	Solute carrier family 11 (proton-coupled divalent metal ion transporters), member 2	1.68±0.33
Ssc.8004.1.A1_at	SLC16A12	Solute carrier family 16, member 12	1.56±0.14
Ssc.7570.1.A1_at	SLC1A1	Solute carrier family 1 (neuronal/epithelial high affinity glutamate transporter, system Xag), member 1	1.53±0.23
Ssc.17849.1.A1_at	SLC30A10	Solute carrier family 30, member 10	2.08±0.68
Ssc.2516.2.S1_a_at	SLC5A6	Solute carrier family 26, member 3	2.21±0.43
Ssc.18227.1.S1_a_at	SLC50A1	Solute carrier family 50 (sugar efflux transporter), member 1	1.54±0.27
Ssc.30277.1.A1_at	SLC26A3	Solute carrier family 26, member 3	-1.58±0.34
**High-dose_liver**			
**Phase I**			
Ssc.203.1.S1_s_at	CYP3A22	Cytochrome P450 3A22	-6.65±1.95
Ssc.16124.1.S1_a_at	CYP2C42	Cytochrome P450 C42	-2.05±0.27
Ssc.204.1.S1_at	CYP3A29	Cytochrome P450 3A29	-1.57±0.28
Ssc.203.1.S1_at	CYP3A39	Cytochrome P450 3A39	-6.78±1.82
Ssc.30334.1.A1_at	CYP3A46	Cytochrome P450 3A46	-1.9±0.34
SscAffx.21.1.S1_at	CYP7A1	Cytochrome P450 7A1	-3.72±0.15
Ssc.2224.1.S1_at	CYP2C19	Cytochrome P450 family 2 subfamily C member 19	-1.51±0.28
**Phase II**			
Ssc.16377.1.A1_at	GSTA2	Glutathione S-transferase alpha 2	-2.63±3.33
Ssc.15549.1.A1_at	GSTT1	Glutathione S-transferase theta-1-like	-1.57±0.32
Ssc.10955.1.S1_at	MGST3	Microsomal glutathione S-transferase 3	-1.7±0.36
Ssc.21161.1.S1_at	UGT1A6	UDP glucuronosyltransferase 1 family, polypeptide A6	-2.17±0.44
**Phase III**			

Ssc.3451.1.S1_at	SLC11A2	Solute carrier family 11 (proton-coupled divalent metal ion transporters), member 2	1.97±0.4
Ssc.4097.1.S1_at	SLC12A4	Solute carrier family 12 (potassium/chloride transporters), member 4	1.56±0.03
Ssc.7570.1.A1_at	SLC1A1	Solute carrier family 1 (neuronal/epithelial high affinity glutamate transporter, system Xag), member 1	1.72±0.31
Ssc.12400.1.S1_at	SLC2A8	Solute carrier family 2 (facilitated glucose transporter), member 8	1.52±0.14
Ssc.17849.1.A1_at	SLC30A10	Solute carrier family 30, member 10	2.21±0.45
Ssc.28782.2.S1_at	SLC38A1	Solute carrier family 38, member 1	2.16±0.24
Ssc.5284.1.S1_at	SLC25A4	Solute carrier family 25 (mitochondrial carrier; adenine nucleotide translocator), member 4	-1.51±0.35
Ssc.25468.1.S1_at	SLC45A2	Solute carrier family 45, member 2	-1.52±0.1
**Low dose_kidney**			
**Phase II**			
Ssc.16377.1.A1_s_at	GSTA1	Glutathione S-transferase alpha 1	3.23±1.40
Ssc.12999.1.S1_at	GSTP1	Glutathione S-transferase pi 1	1.52±0.19
**Phase III**			
Ssc.2879.1.S1_at	SLC25A25	Solute carrier family 25 (mitochondrial carrier; phosphate carrier), member 25	1.68±0.27
Ssc.828.1.S1_at	SLC26A6	Solute carrier family 26, member 6	1.52±0.18
Ssc.30166.1.A1_at	SLC13A1	Solute carrier family 13 (sodium/sulfate symporter), member 1	-1.50±0.2
Ssc.3909.1.A1_at	SLC16A9	Solute carrier family 16, member 9	-1.71±0.48
Ssc.17240.1.S1_at	SLC4A4	Solute carrier family 4, sodium bicarbonate cotransporter, member 4	-2.34±0.16
**High dose_kidney**			
**Phase I**			
Ssc.204.1.S1_at	CYP3A29	Cytochrome P450 3A29	-1.62±0.67
**Phase II**			
Ssc.8516.1.A1_at	GSTA1	Glutathione S-transferase alpha 1	4.60±1.27
Ssc.12273.1.A1_at	GSTM3	Glutathione S-Transferase Mu 3	-2.59±0.31
**Phase III**			
Ssc.4483.1.A1_at	SLC19A3	Solute carrier family 19 (thiamine transporter), member 3	2.07±0.52
Ssc.2879.1.S1_at	SLC25A25	Solute carrier family 25 (mitochondrial carrier; phosphate carrier), member 25	2.19±0.57
Ssc.5887.1.A1_at	SLC37A2	Solute carrier family 37 (glucose-6-phosphate transporter), member 2	1.54±0.11
Ssc.8710.1.A1_at	SLC4A7	Solute carrier family 4, sodium bicarbonate cotransporter, member 7	1.51±0.16
Ssc.1611.1.S1_at	SLC6A19	Solute carrier family 6 (neutral amino acid transporter), member 19	1.68±0.3
Ssc.24556.1.S1_at	SLC35F5	Solute carrier family 35, member F5	-1.53±0.08
Ssc.23758.2.A1_at	SLC46A3	Solute carrier family 46, member 3	-1.55±0.35
Ssc.17240.1.S1_at	SLC4A4	Solute carrier family 4, sodium bicarbonate cotransporter, member 4	-2.33±0.42
