# Supplementary material for: Diclofenac Disrupts the Circadian Clock and through Complex Cross-Talks Aggravates Immune-Mediated Liver Injury—A Repeated Dose Study in Minipigs for 28 Days
Source: Int J Mol Sci. 2023 Jan 11;24(2):1445. doi: 10.3390/ijms24021445 (PMC9863319; doi:10.3390/ijms24021445)
Supplement: Supplementary file 1 [file ijms-24-01445-s001.zip › Supplementary Table S6.pdf]

Supplementary Table S6: Primer sequences used in real-time qRT-PCR analyses

Probeset ID	Gene symbol	Forward (5'-3')	Reverse (3'-5')
	18s rRNA_v1	ccgtcggcatgtattagctc	accgtctcctacctggttga
Ssc.30843.1.A1_at	ADAMDEC1	ttctgcttgcctgactcgta	aggtgagccatgcagacttc
Ssc.18933.1.A1_at	ADH4	tggatgcactggtgtctcat	tcatggctcattgttcatca
Ssc.4891.1.A1_at	AGPAT9	cgactcaagcgggagtaaga	gtcgcttacgctacggagtc
Ssc.6943.1.A1_at	ANGPT1	tgatattttggatgcagttttca	catgtgtattgttttaagggcttg
Ssc.8980.1.A1_at	ANGPTL4	actcttcacctgcctgagga	gcacccacatacaacagcac
Ssc.1039.1.S1_at	APOC3	gacacctcccttctggacaa	ggactccttcacgctggtta
Ssc.833.1.S1_at	C1QA	cttccaggtggtgtccaagt	tggatccagaccttgtctcc
Ssc.17026.1.Sa_at	C4A	gcctctttgagacgagcatc	ctaagtgaaggcggcaagag
Ssc.10460.1.S1_at	CFB	aaggaaagctgcctgtgaga	agtgttggggtcagcatagg
Ssc.864.1.S1_at	CFH	cagtctatcctccgggaaca	gcattttggtggttctgacc
Ssc.16157.1.S1_at	CRP	taggaaccatgagggtgagg	ggggtcaaatcggaactaca
Ssc.2033.1.S1_at	CRY1	ttaggacaaaatttttcttgaaatc	aaatttgggtcaaaacaagca
Ssc.19692.1.S1_at	CXCL2	cagggagggagttctctcaa	atgtccaagggaaacgtcaa
Ssc.208.1.S1_at	CYP1A1	ctcccatctacatgcccact	agagcctggtccaaagaatg
Ssc.203.1.S1_s_at	CYP3A22 / CYP3A39	cgccctgaaaggttcagtaa	ggggcgtctgtgtttcttta
Ssc.203.1.S1_at	CYP3A22 / CYP3A39	cctgaaactaagctctcaagga	caaggtctctgatgctctgg
SscAffx.21.1.S1_at	CYP7A1	caattggcaaacgtttttctt	gcgtgatgtgtccttcaaaa
Ssc.21145.1.S1_at	DEFB1	gctgactgtctgcctcctct	acttggccttgccactgtaa
Ssc.16236.1.S1_at	DPP4	acatgggtcttccaactcca	aaatccactccagcatccac
Ssc.30467.1.A1_at	ELOVL2	tgatgagcagaccagcactt	ccgccccaatcatgatacta
Ssc.13778.1.S1_at	FCGR1A (IgG)	caagtgcaaggtcaacaacg	cgttgctcttccactcaaca
Ssc.16377.1.A1_s_at	GSTA1	tgttgaagagccatggacaa	ttcagcagagggaagttgg
Ssc.390.1.A1_at	HIF1A	tggggcatggtaaaagaaag	gtaatgctcccctcatccaa
Ssc.47.1.S1_at	IGFBP2	catggcctgtacaacctcaa	tggctgtggtttactgcatc
Ssc.15588.1.S2_at	IGFBP3	agcccctgtaccgtaatcct	ggcagaacgtctgaagatcc
Ssc.286.1.S1_s_at	IRG6 (RSAD2)	caccatgcgggaattctact	tggaaaatgcctgacaatga
Ssc.14340.1.S1_at	LITAF	ccggtggtatgtttggaaag	aaaccaactctggcttgtgg
Ssc.670.1.S1_at	LYZ	aaggagcagaatgccagatg	cttcatggtcagcaaaatgc
Ssc.548.1.S1_a_at	MMP7	tggccattctttaggtctgg	tgaatgtgtgttctccctgaa
Ssc.600.1.S1_s_at	MT1A	actgctcctgccccacag	acagcagctgcacttgtcc
Ssc.9372.1.A1_at	NFKBIZ	ggaaacatgctttgctggtt	ccaaatacattccttgggaca
Ssc.13870.1.A1_at	NR1D2	tcaagtatcctttttcctttcca	tgcttcggatcaaatcaaga
Ssc.15928.1.A1_at	NR3C1	ccaggtcacttcccatcact	gtctcagccagactccaagc
Ssc.6323.1.S1_at	PLIN2	tctgatggcctcctcagttc	tctcaggttgctgggtctct
Ssc.8177.1.A2_at	PLP1	agaagcttccaggctgcata	taggaggtctccagggaagg
Ssc.14490.1.S1_at	PTGR1	catgaacacatcacggaagg	tggactctcgtgtcgtcttg
Ssc.2381.1.A1_at	S100A9	caaatgtcgcagatggaatg	caggtcttccaggatgtggt
Ssc.7493.1.A1_at	SIRT1	cgcagagaagcatttattttcc	ctcttctccggcagtaatgg
